# Supplementary material for: Correlative single-molecule and structured illumination microscopy of fast dynamics at the plasma membrane
Source: Nat Commun. 2024 Jul 10;15:5813. doi: 10.1038/s41467-024-49876-9 (PMC11236984; doi:10.1038/s41467-024-49876-9)
Supplement: Supplementary file 1 — Supplementary Information [file 41467_2024_49876_MOESM1_ESM.pdf]

Supplementary Information

Correlative single-molecule and structured illumination microscopy of fast dynamics at the plasma membrane

Hauke Winkelmann, Christian P. Richter, Jasper Eising, Jacob Piehler* & Rainer Kurre*

*Correspondence should be addressed to R.K. (rainer.kurre@uos.de) or J.P. (piehler@uos.de)

This PDF file includes:

Supplementary Tables 1 to 3

Supplementary Figures 1 to 15

Supplementary Tables

Supplementary Table 1: Fitting results for intensity distributions from reHaloTagF PAINT experiments (Supplementary Fig. 4) under different illumination conditions.

			Fitting Gaussian							
**Mode**	**Area**	**# Locs**	**Peak 1**	**Error* (Peak 1)**	**Std 1**	**Error* (Std 1)**	**Peak 2**	**Error* (Peak 1)**	**Std 2**	**Error* (Std 2)**
1-beam	**A1**	130,792	49.4	1.1	15.1	1.2	171.4	1.9	51.3	2.0
Gaussian	**A2**	136,932	41.6	1.2	11.8	1.3	133.3	1.3	39.5	1.5
TIRF	**A3**	134,227	36.1	1.4	9.7	1.4	110.7	0.8	30.8	0.9
	**A4**	135,099	30.6	1.5	5.9	3.6	93.0	0.8	28.9	0.8
	**A5**	130,453	76.7	0.6	26.0	0.6	N/A	N/A	N/A	N/A
	**A6**	108,093	63.5	1.7	27.4	1.7	N/A	N/A	N/A	N/A
	**QV**	251,066	69.6	14.3	32.5	19.7	122.8	137.9	49.2	52.2
	**FoV**	830,809	44.4	1.1	12.1	1.2	146.2	1.8	53.3	2.0
1-beam	**A1**	184,884	44.2	1.3	13.1	1.3	152.8	1.4	36.9	1.4
flat-field	**A2**	198,020	42.2	1.1	12.0	1.1	138.2	1.0	37.4	1.1
TIRF	**A3**	193,731	41.7	1.4	11.8	1.4	137.3	1.4	38.0	1.5
	**A4**	194,115	40.3	1.2	11.2	1.3	134.7	1.2	39.0	1.3
	**A5**	193,534	38.0	2.0	9.0	2.3	107.2	1.6	38.9	1.8
	**A6**	165,053	39.8	1.2	10.3	1.6	99.1	2.6	42.3	2.6
	**QV**	361,164	40.8	0.9	11.6	0.9	129.5	1.2	43.7	1.4
	**FoV**	1,217,946	43.2	1.1	12.6	1.2	145.4	1.2	37.7	1.2
Gaussian	**A1**	83,392	49.2	0.7	14.2	0.7	157.4	1.0	46.7	1.1
SI-TIRF	**A2**	91,404	40.4	0.8	10.5	0.8	122.5	0.5	33.1	0.6
	**A3**	94,844	34.2	1.2	8.1	1.1	97.6	0.4	26.1	0.5
	**A4**	96,447	29.9	1.0	5.0	7.9	79.9	0.4	21.9	0.4
	**A5**	90,991	68.3	0.3	17.8	0.3	N/A	N/A	N/A	N/A
	**A6**	85,403	59.5	0.4	16.3	0.4	N/A	N/A	N/A	N/A
	**QV**	163,821	63.3	1.1	21.0	2.5	109.4	17.3	41.3	8.5
	**FoV**	595,733	43.9	1.2	11.0	1.3	132.6	1.3	46.7	1.5
flat-field	**A1**	158,048	44.8	1.3	12.7	1.3	156.2	1.1	29.4	1.1
SI-TIRF	**A2**	154,854	42.8	1.1	12.1	1.2	147.0	1.0	31.3	1.0
	**A3**	161,424	43.0	1.4	12.3	1.5	143.4	1.4	36.7	1.5
	**A4**	161,557	41.9	1.6	11.5	1.6	139.7	1.7	36.1	1.8
	**A5**	164,335	41.6	1.6	12.0	1.6	140.2	1.2	31.8	1.2
	**A6**	153,567	42.9	1.0	12.4	1.0	147.3	0.8	29.3	0.8
	**QV**	296,888	42.9	1.1	12.8	1.1	145.4	1.0	34.0	1.1
	**FoV**	1,069,537	44.0	1.2	12.5	1.2	151.3	1.0	31.2	1.0

*error calculations are based on 95% confidence interval derived from Gaussian fits.

Supplementary Table 2: Description of imaging and emitter parameters of simulations.

Imaging parameter	Description
**Exposure time Δt [ms]:**	Fixed exposure time of each simulated frame and frame rate of simulation (typical: 5-50 ms, default: 25 ms)
**Imaging width and height [pxl]:**	Resolution of simulated imaging frames (typical: 64-512)
**Pixel size Δr [nm]:**	Pixel size of final simulated images (default: 100 nm)
**Activation cycles:**	sptPALM: cycles of photoactivation; for PAINT: total number of frames; for SPT: set to 1.
**Frames per cycle:**	sptPALM: number of frames per photoactivation cycle; for PAINT: Set to 1; for SPT: total number of frames.
**Emission wavelength λ_{em} [nm]:**	Wavelength of single-emitter peak fluorescence in units of nanometer.
**Numerical aperture NA:**	Numerical aperture of objective lens.
**Quantum efficiency QE:**	Quantum efficiency of camera detector to calculate photons from photoelectrons.
**Electron conversion ecf [e^-/count]:**	Photoelectron conversion factor to translate digital counts into photoelectrons.
**Camera offset [counts]:**	Camera offset in digital counts measured by averaging dark frames.
**Camera noise [counts]:**	Camera noise in digital counts measured by standard deviation of dark frames.
**Illumination profile:**	Evanescent field with Gaussian intensity profile defined by standard deviation or a flat-top illumination profile.
**Penetration depth d_p of evanescent field [nm]:**	The evanescent field is modeled by a single exponential decaying function.
Emitter parameters	
**Emitter intensity S_e [photons]:**	Integrated signal of a single-emitter in units of photons per frame.
**Background intensity S_bg [photons]:**	Averaged background intensity on single pixel level in units of photons.
**Particle density [1/µm^2]:**	Mean particle (monomers, dimers, …) density per frame.
**Lifetime τ_e [frames]:**	The mean lifetime (binding/unbinding, bleaching) of single emitter modeled by single exponential decay.
**Mean axial position z_0 [nm]:**	The averaged axial position of each single emitter equals focal plane above cover slip.
**Oligomerization state:**	Set to 1 for monomers, 2 for dimers, 3 for trimers, ….
**Fraction of oligomers:**	Number of particles based on given particle density and imaging area are split into monomers and oligomers by specified fraction.
**Diffusion constant of monomers D_m [µm^2/s]:**	Diffusion constant to model two-dimensional random walk of monomers by mean displacement $\bar{d}_\mathrm{m} = \sqrt{2D_\mathrm{m}\Delta t}$.
**Diffusion constant of oligomers D_o [µm^2/s]:**	Diffusion constant to model two-dimensional random walk of oligomers by mean displacement $\bar{d}_\mathrm{o} = \sqrt{2D_\mathrm{o}\Delta t}$.
**Standard deviation σ_z of axial position [nm]:**	Variation of axial position modelled by normal distribution with mean z_0 and standard deviation σ_z.

Supplementary Table 3: Conditions of all experiments and simulations.

Figure (Movie)	Sample (T [°C])	Fluorescent label	Excitation Wavelength [nm]	Image pixels (x, y)	Exposure time [ms]	Power density [W/cm^2]
**1b-c (S1)**	Dye solution in PBS (25)	Fluorescein, Texas Red & Atto 655	488, 560, 642	512 x 512 (EMCCD) 256 x 256 (QV channels)	50	5
**1d-g (S2)**	Immobilized reHaloTags on tris-NTA chip (25)	reHaloTag/MaP555	560	256 x 256 (EMCCD)	25	100
**2c-d, 2g (S3, S4)**	HeLa (25) transfected w/ ALFA-mXFP-TpoR	Atto 643-EN	642	256 x 256 (EMCCD)	25	200
**2e-f, 2h (S3, S5)**	HeLa (25) transfected w/ ALFA-mXFP-TpoR + tdALFAnb	Atto 643-EN	642	256 x 256 (EMCCD)	25	200
**3b-i (S6), 3f (S7)**	HeLa (25) transfected w/ ALFA-mXFP-TpoR + tdALFAnb	Cy3B-EN, Atto 643-EN	560, 642	512 x 256 (EMCCD) 256 x 256 (QV channels)	25	100
**4b-d (S8)**	HeLa (25) transfected w/ mXFP-TpoR	Atto 643-EN	642	256 x 256 (EMCCD)	20	100
**4e-f (S9)**	HeLa (25) transfected w/ mXFP-TpoR	Cy3B-EN, Atto 643-EN	560, 642	512 x 256 (EMCCD) 256 x 256 (QV channels)	32	100
**4g-i (S10)**	HeLa (25) transfected w/ mXFP-TpoR	Atto 643-EN	642	256 x 256 (EMCCD)	32	100
**5, S13 (S13-16)**	HeLa (25) co-transfected w/ mXFP-TpoR and LifeAct-HaloTag	LifeAct-HaloTag/JFX549, Atto 643-EN	560, 642	256x256 (EMCCD) 300x 300 (CMOS)	32 (EMCCD), 10.62 (CMOS)	100
**6a-d, S14a-f (S17)**	HeLa (25) co-transfected w/ ALFA-mXFP-TpoR and StayGold-tdmFYVE and Jak2-tdmCherry + 10 nM TPO	StayGold-tdmFYVE, Atto 643-EN	488, 642	256x256 (EMCCD) 300x 300 (CMOS)	32 (EMCCD), 10.62 (CMOS)	100
**6e-g (S18-20)**	HeLa (25) co-transfected w/ ALFA-mXFP-TpoR and LifeAct-StayGold + 10 nM TPO	LifeAct-StayGold, Atto 643-EN	488, 642	256x256 (EMCCD) 300x 300 (CMOS)	32 (EMCCD), 10.62 (CMOS)	100
**S2c-f**	Dye solution in PBS (25)	Texas Red	560	512 x 512 (EMCCD)	50	5
**S3**	Immobilized reHaloTags on tris-NTA chip (25)	reHaloTag/MaP555	560	512 x 512 (EMCCD)	50	75

Figure (Movie)	Sample (T [°C])	Fluorescent label	Excitation Wavelength [nm]	Image pixels (x, y)	Exposure time [ms]	Power density [W/cm^2]
**S4b-d (S2)**	Simulation of reHaloTag PAINTing. Main parameters: Exposure time: 50 ms, resolution: 256x256, pixel size: 100nm, activation cycle: 4000, frames per cycle: 1, Detection wavelength: 580 nm, Numerical aperture: 1.5, quantum efficiency: 0.95, photon conversion factor: 0.015 e-/cnt, camera offset: 170 cnts, camera noise: 18 cnts, illumination profile: flat-top, penetration depth evanescent field: 120 nm, Emitter intensity: 100 ph, BG intensity: 1 ph, particle density: 0.01 µm^-2, lifetime: 10 frames, fraction of oligomers: 0, diffusion monomer: 0 µm^2/s, mean z position: 0 nm, std z position: 0 nm.					
**S4g**	Simulation of single receptor diffusion (monomers and dimers) with different axial z position variation. Main parameters: Exposure time: 25 ms, resolution: 256x256, pixel size: 100nm, activation cycle: 1, frames per cycle: 200, Detection wavelength: 680 nm, Numerical aperture: 1.5, quantum efficiency: 0.92, photon conversion factor: 0.015 e-/cnt, camera offset: 170 cnts, camera noise: 18 cnts, illumination profile: flat-top, penetration depth evanescent field: 120 nm, Emitter intensity: 100 ph, BG intensity: 1 ph, particle density: 0.1 µm^-2, lifetime: 2000 frames, oligomerization state: 2, fraction of oligomers: 0 or 0.2, diffusion monomer: 0.13 µm^2/s, diffusion dimer: 0.1 µm^2/s. mean z position: 30 nm, std z position: 0 nm, 10 nm, or 20 nm.					
**S5a-e**	Gattaquant PAINT 40RY (25)	Cy3B, Atto 655	560, 642	512 x 256 (EMCCD) 256 x 256 (QV channels)	200	250
**S5f-g**	HeLa (25) transfected w/ farnesyl-GFP	farnesyl-GFP	488	256 x 256 (EMCCD)	32	N/A
**S6c-d, S6g**	HeLa (25) transfected w/ ALFA-mXFP-TpoR and Jak2ΔTK-mEGFP	Atto 643-EN	642	256 x 256 (EMCCD)	25	200
**S6e-f, S6h**	HeLa (25) transfected w/ ALFA-mXFP-TpoR and Jak2ΔTK-mEGFP + 10nM Tpo	Atto 643-EN	642	256 x 256 (EMCCD)	25	200
**S6j-l**	Simulation of single receptor diffusion (monomers and dimers) to match experiment shown in S6e-f. Main parameters: Exposure time: 25 ms, resolution: 256x256, pixel size: 100nm, activation cycle: 1, frames per cycle: 200, Detection wavelength: 680 nm, Numerical aperture: 1.5, quantum efficiency: 0.92, photon conversion factor: 0.015 e-/cnt, camera offset: 170 cnts, camera noise: 18 cnts, illumination profile: flat-top, penetration depth evanescent field: 120 nm, Emitter intensity: 400 ph, BG intensity: 1 ph, particle density: 0.1 µm^-2, lifetime: 2.000 frames, oligomerization state: 2, fraction of oligomers: 0.2, diffusion monomer: 0.13 µm^2/s, diffusion dimer: 0.1 µm^2/s. mean z position: 30 nm, std z position: 0 nm (S6j) and 25 nm (S6k-l).					
**S7d-e**	HeLa (25) transfected w/ ALFA-mXFP-TpoR + tdALFAnb	Cy3B-EN, Atto 643-EN	560, 642	512 x 256 (EMCCD) 256 x 256 (QV channels)	25	100
**S8a-c**	HeLa (25) transfected w/ mXFP-TpoR	Atto 643	642	512 x 512 (EMCCD)	32	200
**S8d-f**	HeLa (25) transfected w/ mXFP-TpoR	Cy3B-EN, Atto 643-EN	560, 642	512 x 256 (EMCCD) 256 x 256 (QV channels)	32	100
**S9**	TetraSpeck, 100 nm (25)	N/A	488, 560, 642	600 x 600 (CMOS)	10.62	10

Figure (Movie)	Sample (T [°C])	Fluorescent label	Excitation Wavelength [nm]	Image pixels (x, y)	Exposure time [ms]	Power density [W/cm^2]
**S10**	TetraSpeck, 100 nm (25)	N/A	488, 560, 642	600 x 600 (CMOS) 300 x 300 (QV channels)	10.62	10
**S11**	HeLa (25) transfected w/ LiveAct-StayGold	LiveAct-StayGold	488	600 x 600 (CMOS)	10.62	50
**S12 (S11)**	HeLa (25) transfected w/ TOM20-meGFP	TOM20-meGFP, Abberior Live Orange, SPY650-tubulin	488, 560, 642	600 x 600 (CMOS) 300 x 300 (QV channels)	10.62	50
**S13 (S12)**	HeLa (25) transfected w/ TOM20-halo	TOM20-HaloTag/JFX549+JFX646	560, 642	256x256 (EMCCD) 300x 300 (CMOS)	32 (EMCCD), 10.62 (CMOS)	50
**S15g-i**	Simulation of single receptor diffusion (monomers and dimers) to match experiment shown in Figure 6e-g and S14a-f. Main parameters: Exposure time: 33 ms, resolution: 256x256, pixel size: 100nm, activation cycle: 1, frames per cycle: 300, Detection wavelength: 680 nm, Numerical aperture: 1.5, quantum efficiency: 0.92, photon conversion factor: 0.015 e-/cnt, camera offset: 170 cnts, camera noise: 18 cnts, illumination profile: flat-top, penetration depth evanescent field: 120 nm, Emitter intensity: 110 ph, BG intensity: 1 ph, particle density: 0.6 µm^-2, lifetime: 20.000 frames, oligomerization state: 2, fraction of oligomers: 0.13, diffusion monomer: 0.16 µm^2/s, diffusion dimer: 0.1 µm^2/s. mean z position: 30 nm, std z position: 25 nm.					

Supplementary Figures

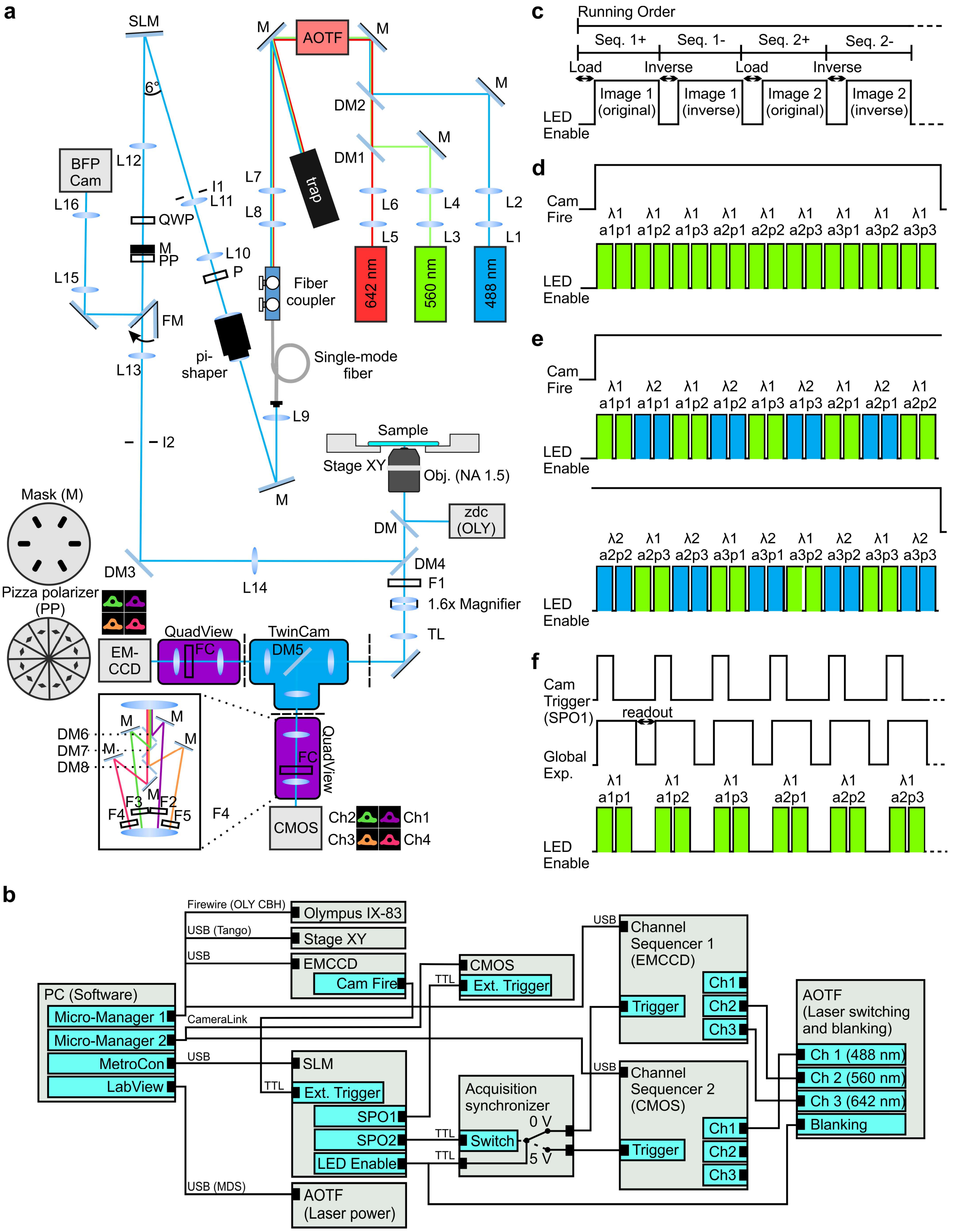

Supplementary Fig. 1: Microscope and hardware synchronization design for (combined) single-molecule imaging (SMI) and super-resolution SIM. a Technical drawing of microscope setup (details in method section). L#: lens, M: mirror, AOTF: acousto-optical tunable filter, I: iris, P: polarizer, FM: flip mirror, SLM: spatial light modulator, QWP: quarter-wave plate: M: mask, PP: pizza polarizer, DM: dichroic/polychroic mirror, F#: bandpass filter, TL: tube lens: FC: filter cube, Ch#: fluorescence channel. **b** Diagram of

software control and hardware-based fast triggering for combined single-molecule imaging using EMCCD and SIM using CMOS camera. (details in method section). Connections shown allow for 2-color SMI (560 nm & 642 nm) and 1-color SIM (488 nm). Other configurations require reprogramming of the two channel sequencers and correct physical connection with the AOTF channels. The acquisition synchronizer is controlled by the SLM (SPO2) to send correct trigger signals ('LED enable') to the sequencers. **c** Diagram showing architecture of running orders programmed on the SLM. A running order links a sequence file (timing of image display) with a binary image to be displayed. Each image needs to be inverted and displayed again to guarantee DC balancing. The 'LED enable' TTL signal was used for laser blanking and as laser channel sequence trigger. **d** Diagram for 1-color SMI imaging with homogenous illumination. Nine different binary images are sequentially loaded to the SLM during a single exposure of the EMCCD camera ('Cam Fire' signal). Each image corresponds to a specific wavelength (λ#, lambda), pattern orientation (a#, angle) and pattern phase (p#, phase). **e** Diagram for simultaneous 2-color SMI for live-cell single-molecule tracking. Three-color SMI follows the same principle. **f** SIM requires external triggering of CMOS camera to synchronize global exposure with laser illumination and correct image display on SLM. Diagram shows acquisition of first six images for 1-color SIM. Multi-color SIM is realized by acquisition of nine SIM frames for each color one after another starting with longest wavelength.

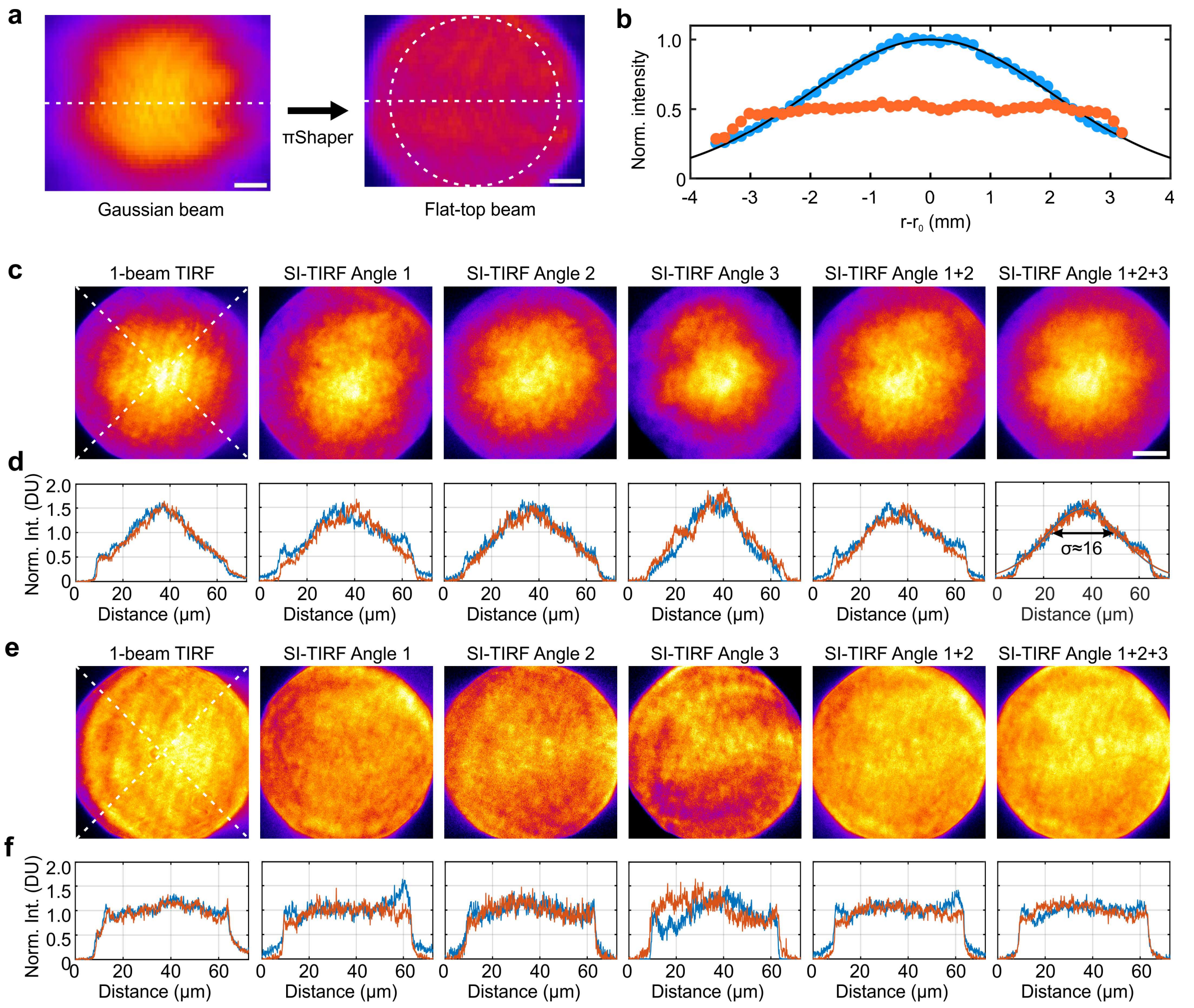

Supplementary Fig. 2: Beam shaping performance and comparison of all available illumination modes. **a** Intensity profile before and after πShaper measured by the inspection camera. Scale bar: 1 mm. Dashed circle indicates beam area illuminating SLM. **b** Line profiles along dashed lines of beam profiles in **a**. Blue circles: Gaussian profile of input beam. Black line: Gaussian fit. Orange profile illustrates clear flat-top profile from πShaper's output. **c-f** Comparison of homogeneity for a single-beam TIRF (1-beam TIRF) versus a single-angle and multi-angular structured illumination TIRF (SI-TIRF). Images of dye solution (TexasRed) and corresponding diagonal line profiles without πShaper (**c**, **d**) and with πShaper (**e**, **f**). Scale bars: 10 µm. **c** and **e** are showing full camera chip of EMCCD camera and maximum circular field of illumination of the system. **d**, **f** Diagonal line profiles from bottom left to top right (blue) and from top left to bottom right corner (orange). All line profiles were normalized to the mean intensity of distance range 10-60 µm. Source data are provided as a Source Data file.

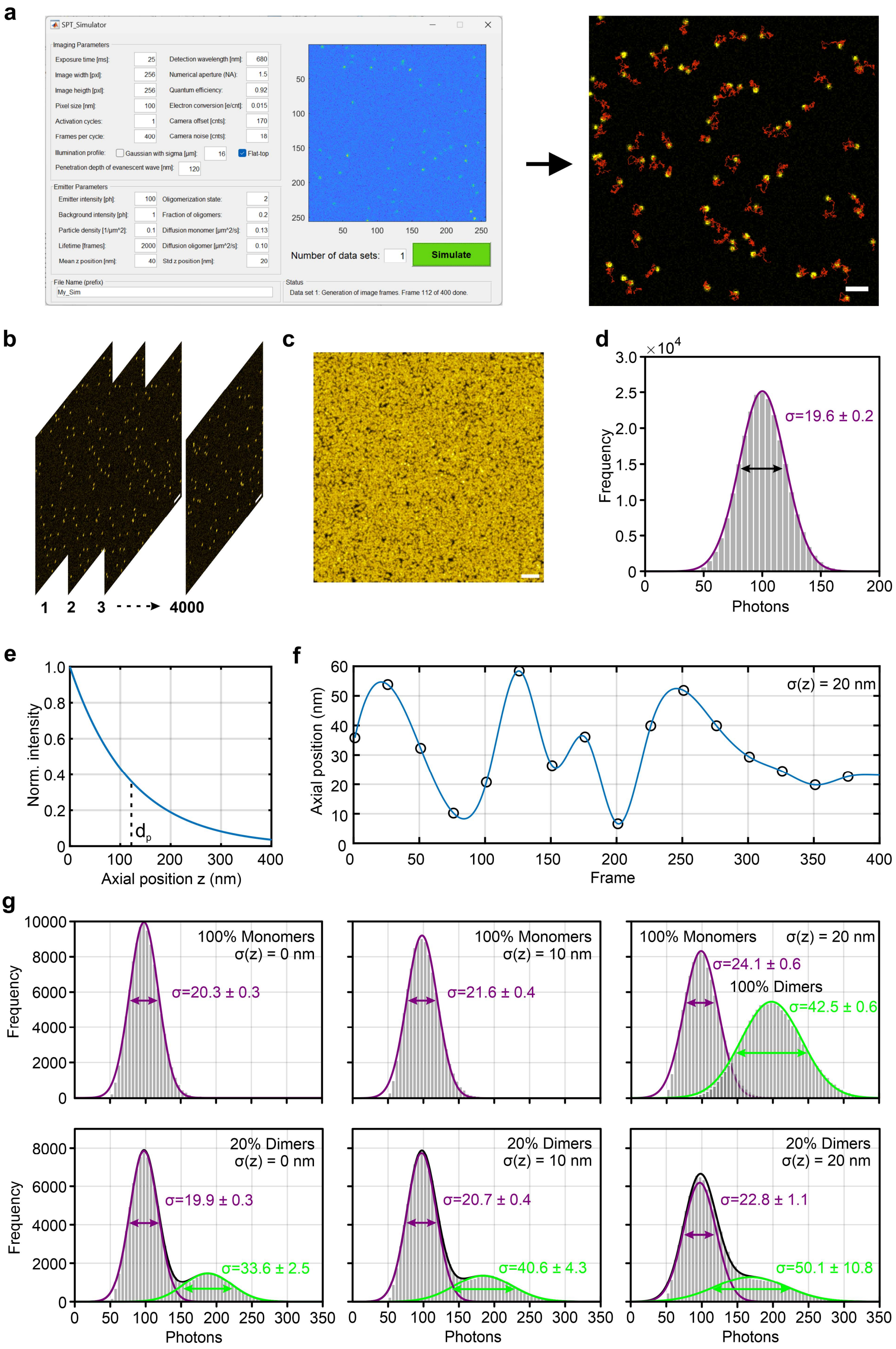

Supplementary Fig. 3: Modelling intensity distributions of PAINT and live-cell single-molecule dimerization assays. **a** Left: Graphical user interface of simulation software SPT_Simulator for modeling PAINT and single-molecule tracking data sets (details in methods section). Right: Example of a modeled single-molecule image frame (yellow) with overlaid trajectories (red). Scale bar: 2 µm. **b-d** Modeling of reHaloTag PAINT experiments with a mean single-emitter intensity of 100 photons. Simulation is based on 4.000 frames with a mean binding time of 10 frames and an initial particle density of 0.01 µm^{-2}. **c-d** Maximum intensity projection (**c**) as well as single-molecule intensity distribution with Gaussian fit (**d**) of ≈ 2.5×10^{5} simulated particles. Standard deviation σ of fit is indicated. **e** Modeled axial

excitation intensity of evanescent field by single exponential function with penetration depth d_p (here: 120 nm). **f** Mimicking plasma membrane ripples of diffusing particles by simulating additional variation of axial position on single trajectory level. Black circles: Nodes of normally distributed random axial positions with a standard deviation of $\sigma = 20$ nm. Distance between nodes (here: 25 frames) is inversely proportional to the mean displacement (details in method section). Final axial variation (blue line) is modeled by a spline function based on the random axial position at the depicted nodes. **g** Intensity distributions of modeled single-molecule trajectories with increasing axial position variation (left to right: $\sigma = 0$, 10, 20 nm) and different dimerization levels (0% (top) vs. 20% (bottom)). Each histogram (n ≈ $1x10^5$) is based on 10 data sets of 200 frames with a mean particle density of 0.1 μm^{-2}. Monomers and Dimers were modeled with a diffusion constant of 0.13 $\mu m^2/s$ and 0.10 $\mu m^2/s$, respectively. Lifetime of single fluorophores was set to 2.000 frames. Further simulation parameters are listed in Supplementary Table 3. Each histogram was fitted with mono- or bimodal Gaussian functions and standard deviations of peaks are indicated. Source data are provided as a Source Data file.

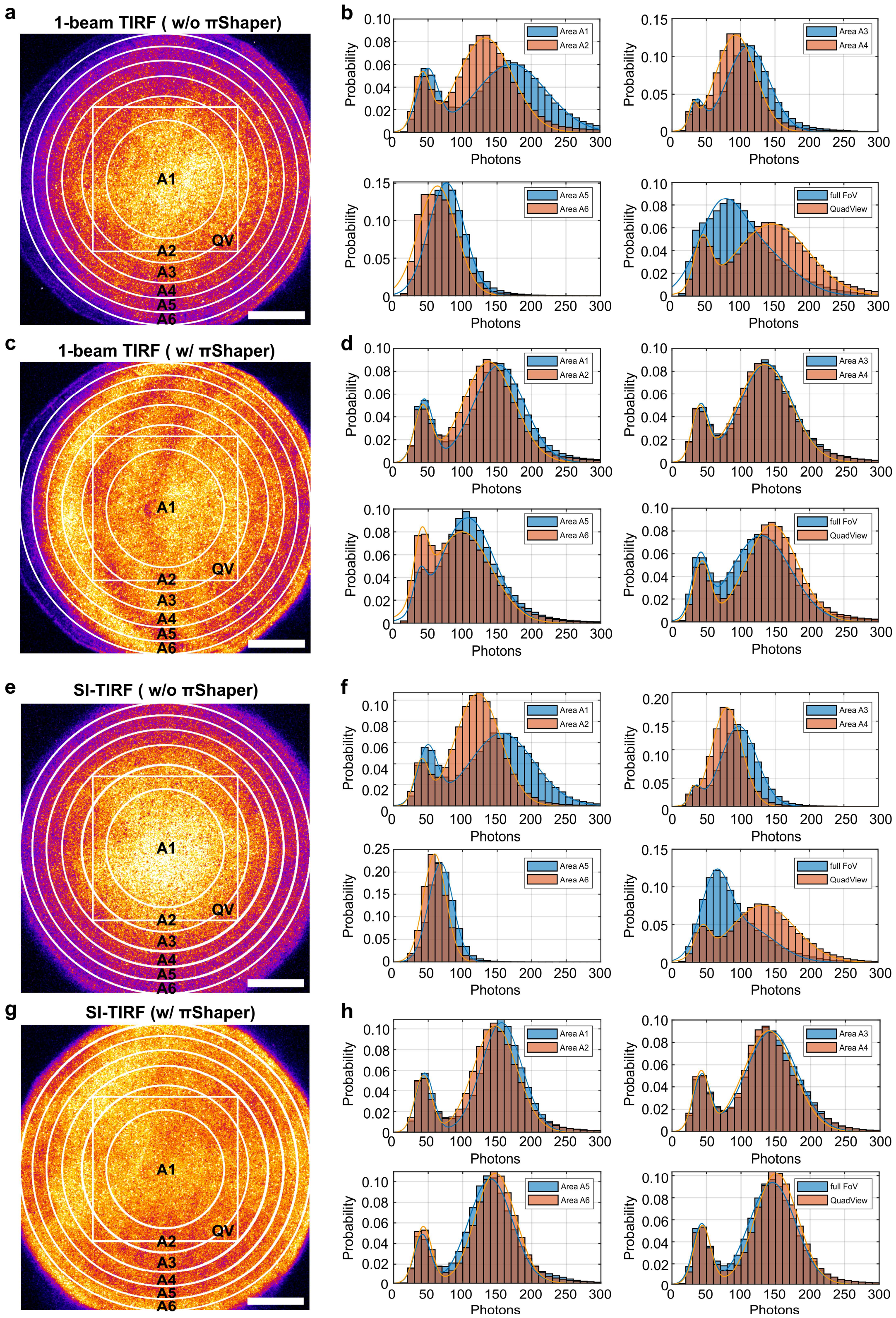

Supplementary Fig. 4: Benchmarking of SI-based TIRFM versus traditional TIRFM by spatial single-molecule intensity-analysis. a, **c**, **e**, **g** Maximum intensity projections of reHaloTagF PAINTing with 50-150 pM MaP555-HTL for 1-beam TIRFM (**a**, **c**) and 3-angular SI-TIRFM (**e**, **g**). Field of illumination was split into 6 equally-sized centered circular areas (A1, A2, …, A6) and the image splitter (QuadView, QV) and for each area and illumination mode single-molecule intensity histograms were plotted (**b**, **d**, **f**, **h**). Statistics and fitting results are summarized in Supplementary Table 1. Source data are provided as a Source Data file.

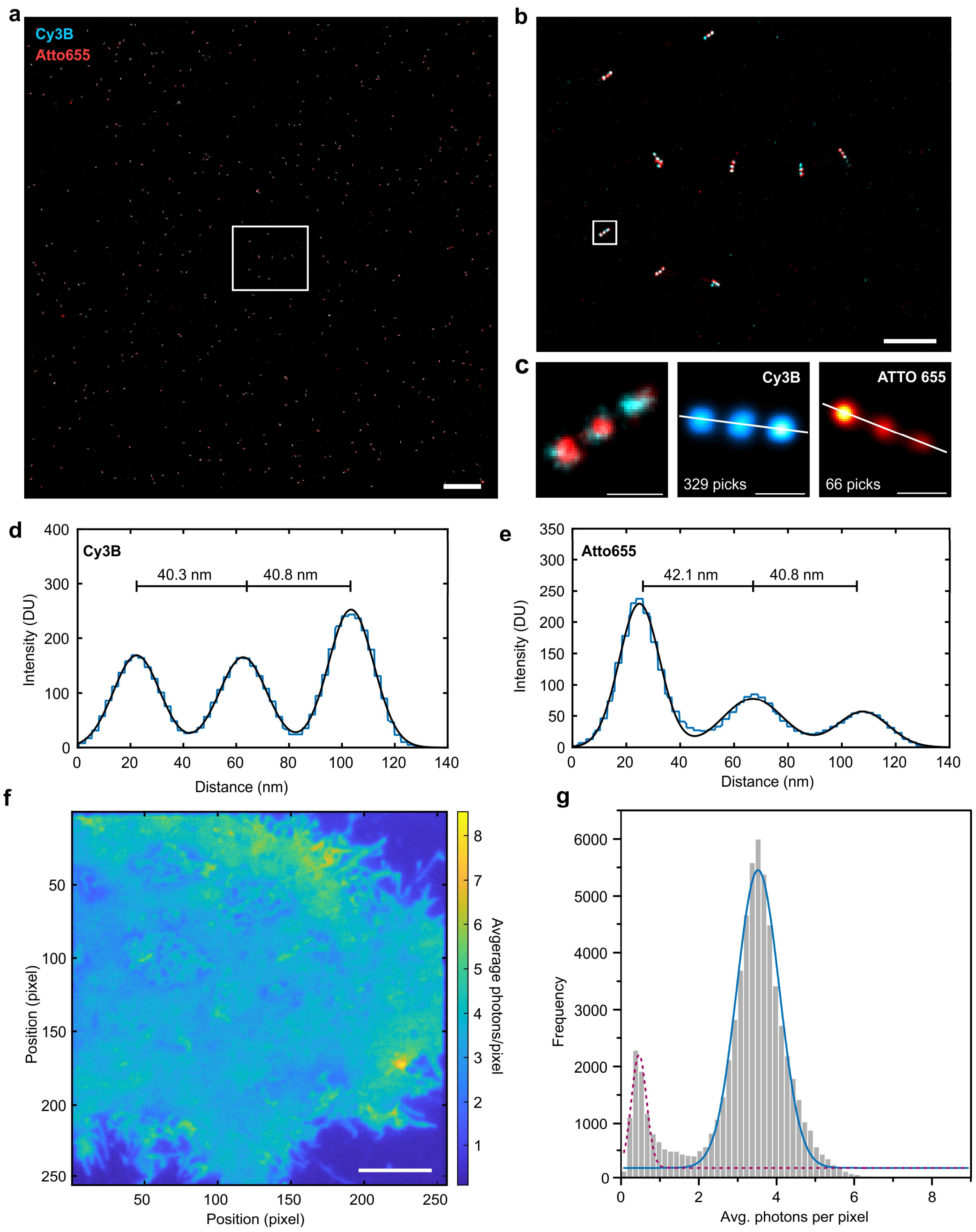

Supplementary Fig. 5: Homogenous TIR illumination for single-molecule super-resolution and stained plasma membrane in live cells. **a-e** Dual-color DNA PAINT of DNA origamis (Gattaquant Nanorulers PAINT 40RY). **a** Overview image and zoom into highlighted region **b** as well as a single nanoruler and averaged structures for the Cy3B and ATTO 655 channels **c**. Number of picked structures for averaging are indicated. **d, e** Intensity cross-sections from averaged nanorulers in **c**. Indicated mean distances of each target on the origami was determined by Gaussian fitting using three populations. **f, g** TIRF imaging of HeLa cells expressing farnesyl-GFP for labeling of the plasma membrane. **f** Average intensity projection from 200 frames. Scale bar: 5 µm. **g** Corresponding pixel intensity histogram. Source data are provided as a Source Data file.

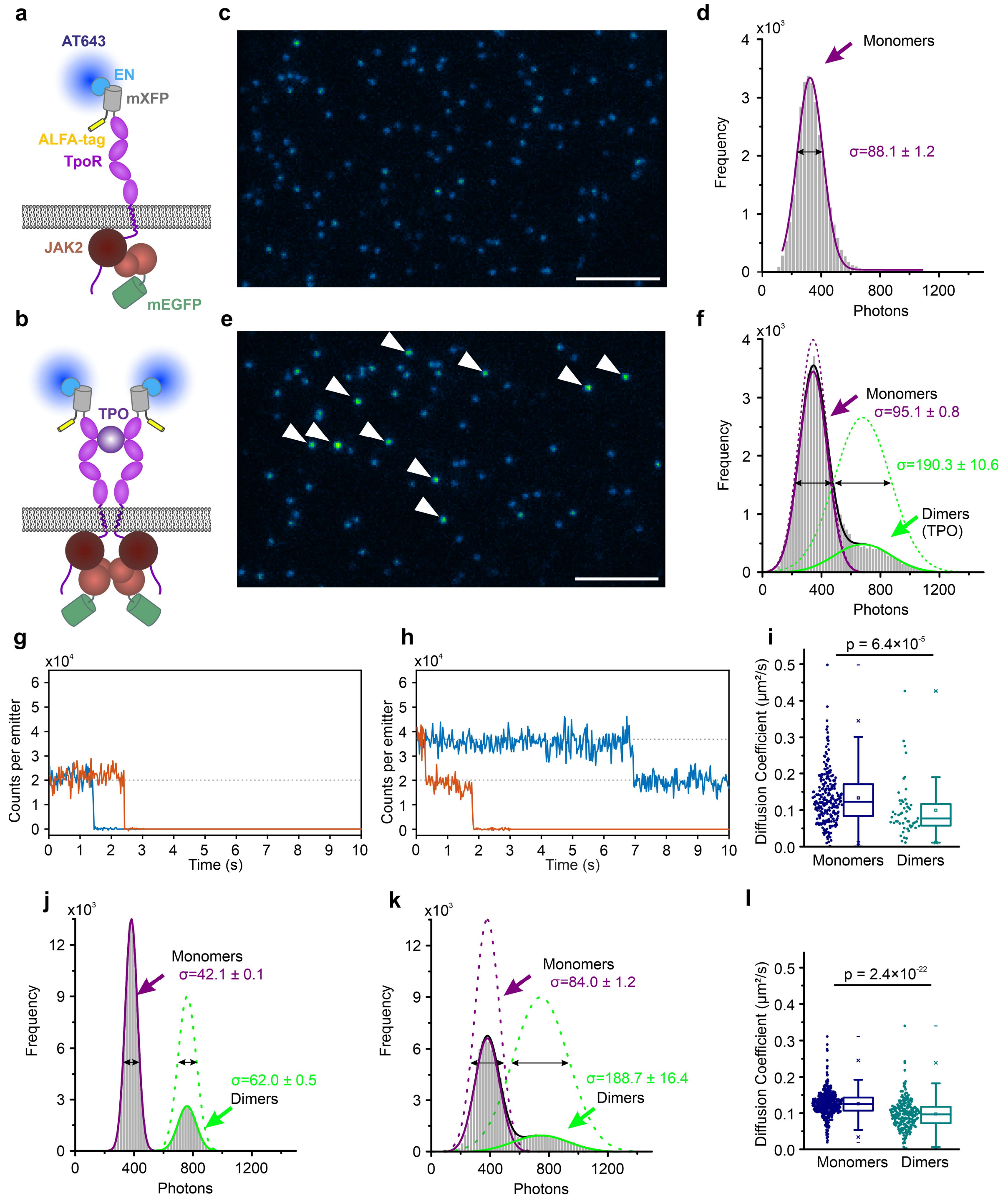

Supplementary Fig. 6: Intensity analysis of ligand-induced TpoR dimerization. a Thrombopoietin receptor (TpoR) N-terminally fused to XFP and ALFA-tag (ALFA-mXFP-TpoR) and JAK2ΔTK C-terminally fused to mEGFP were co-expressed in HeLa cells and labeled using anti-GFP nanobodies (EN) conjugated with ATTO 643. **b** Dimerization of ALFA-mXFP-TpoR by its ligand thrombopoietin (TPO) in the presence of JAK2ΔTK-mEGFP. **c, d** Representative frame from time-lapse single-molecule imaging of monomeric, ^{AT643}EN-labeled ALFA-mXFP-TpoR in a HeLa cell (**c**) and intensity distribution analysis from 11 cells with 3.04×10^4 localizations (**d**). **e, f** Representative frame from time-lapse single-molecule imaging of ^{AT643}EN-labeled mXFP-TpoR in the presence of TPO (**e**) and intensity distribution analysis from 5 cells with 5.21×10^4 localizations (**f**). Scale bar in **c** and **e**: 5 µm. Dimers in **e** are highlighted by white arrows. Histograms were fitted with Gaussian or bimodal Gaussian, standard deviation σ is indicated. **g, h** Representative photobleaching events observed for ^{AT643}EN-labeled ALFA-mXFP-TpoR in the absence (**g**) and presence (**h**) of TPO. **i** Diffusion

coefficient distribution derived from mean-squared displacement analysis. Single-molecule signals were split by an intensity threshold into monomers (< 625 photons) and dimers (> 625 photons) before tracking (monomers: 257 trajectories, dimers: 53 trajectories). Box plot indicates data distribution of the second and third quartiles (box), median (line), mean (square), and 1.5x interquartile range (whiskers). **j-l** Simulation of the dimerization assay based on 20% dimerization and 10 independent data sets (each 200 frames) to match experimental results in **f** (simulation parameters are listed in Supplementary Table 1). **j** Intensity distribution of simulations without an additional intensity variation based on axially modulated single-molecule trajectories showing shot-noise only limited distributions ($n=9.21 \times 10^4$ localizations). **k** Intensity distribution of simulations with additional signal variation based on axial position variation with a standard deviation of 25 nm ($n=8.99 \times 10^4$ localizations). **l** Corresponding diffusion coefficients for monomers and dimers based on the same processing as for **i** (monomers: 483 trajectories, dimers: 238 trajectories). Box plot indicates data distribution of the second and third quartiles (box), median (line), mean (square), and 1.5x interquartile range (whiskers). Statistics for **i** and **l** were performed using two-sample Kolmogorov–Smirnov test. Source data are provided as a Source Data file.

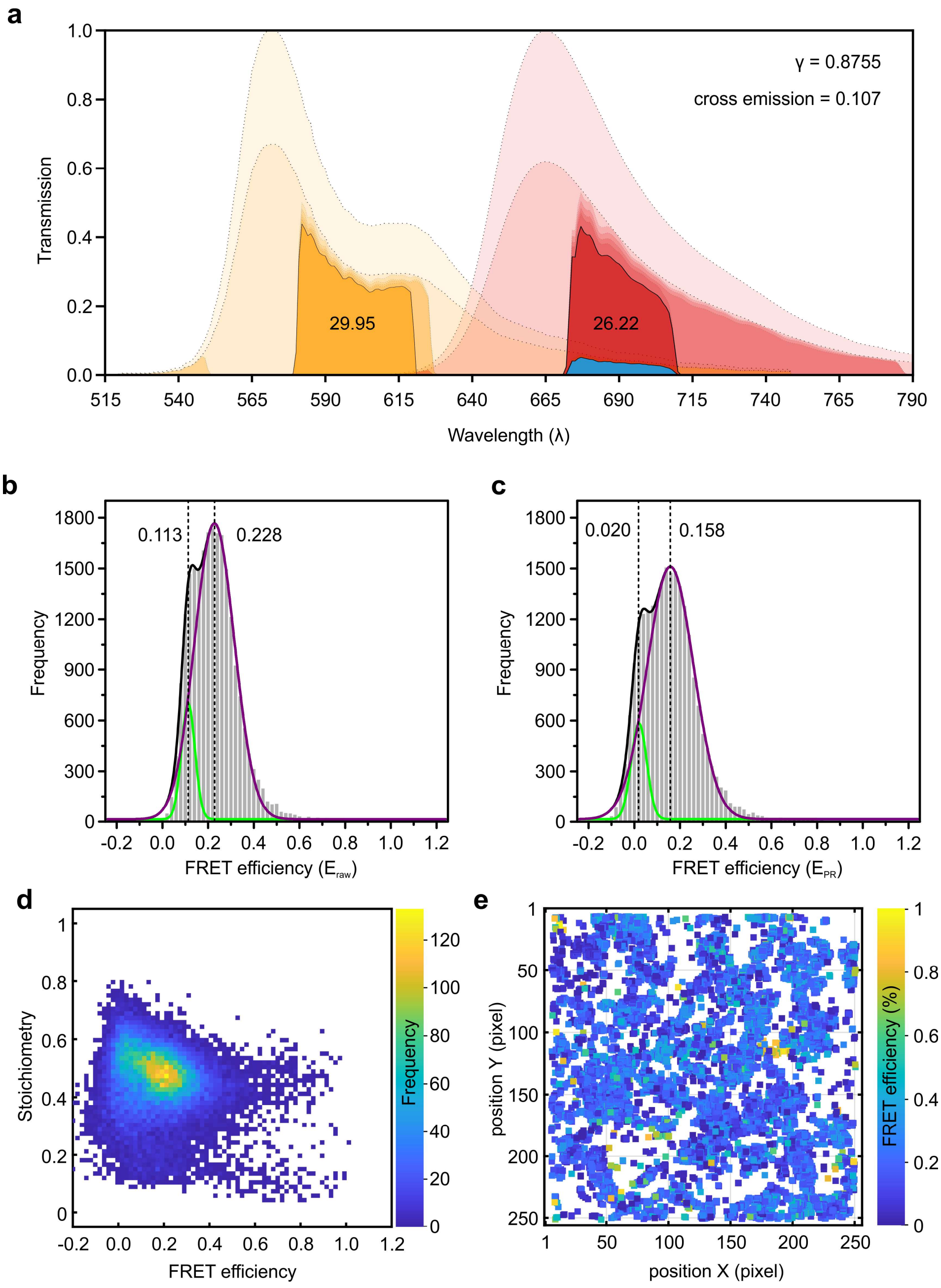

Supplementary Fig. 7: Quantifying smFRET efficiencies. a Calculation of the correction parameters for determining smFRET efficiencies taking the emission spectra of donor (orange) and acceptor (red) into account as well as the transmission characteristics of the emission filters. **b, c** FRET efficiency histogram lacking correction of cross-emission from the donor into the acceptor channel **b** and cross-emission corrected proximity ratio E_{PR} **c**. **d** Plot of the smFRET efficiency vs. the complex stoichiometry for all co-localized donor and acceptor signals. **e** Pooled FRET efficiency map of individual TpoR dimers diffusing in the plasma membrane showing homogeneity in space and time. Histograms were fitted with a bimodal Gaussian. Source data are provided as a Source Data file.

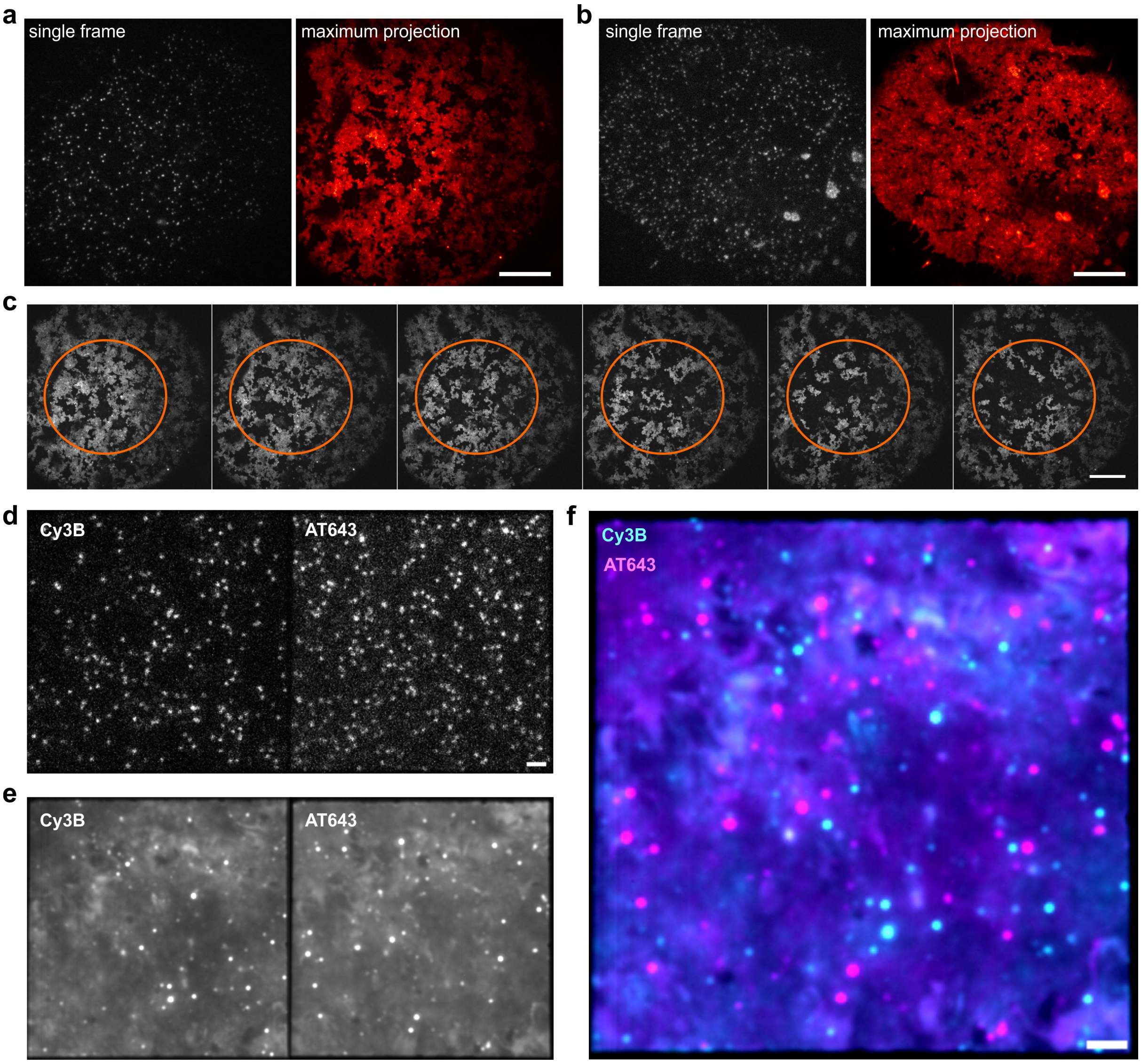

Supplementary Fig. 8: Long-term single and dual-color tracking and localization microscopy (TALM). a, b Single-molecule imaging of mXFP-TpoR labeled with ^{AT643}EN upon Gaussian **a** and flat-top illumination **b**. A single frame (left) and a maximum intensity projection from 1,000 consecutive frames (right) are shown in each panel. **c** Biased photobleaching upon illumination with a Gaussian beam profile, images sequentially show a maximum projection of 500 consecutive frames, of total 3,000 frames. **d-f** Dual-color TALM at high molecule density. Single frames **d** and average intensity projection of 9,000 consecutive frames **e**. **f** Overlay of the average intensity projections of both channels shown in **e**. Scale bar: 2 µm.

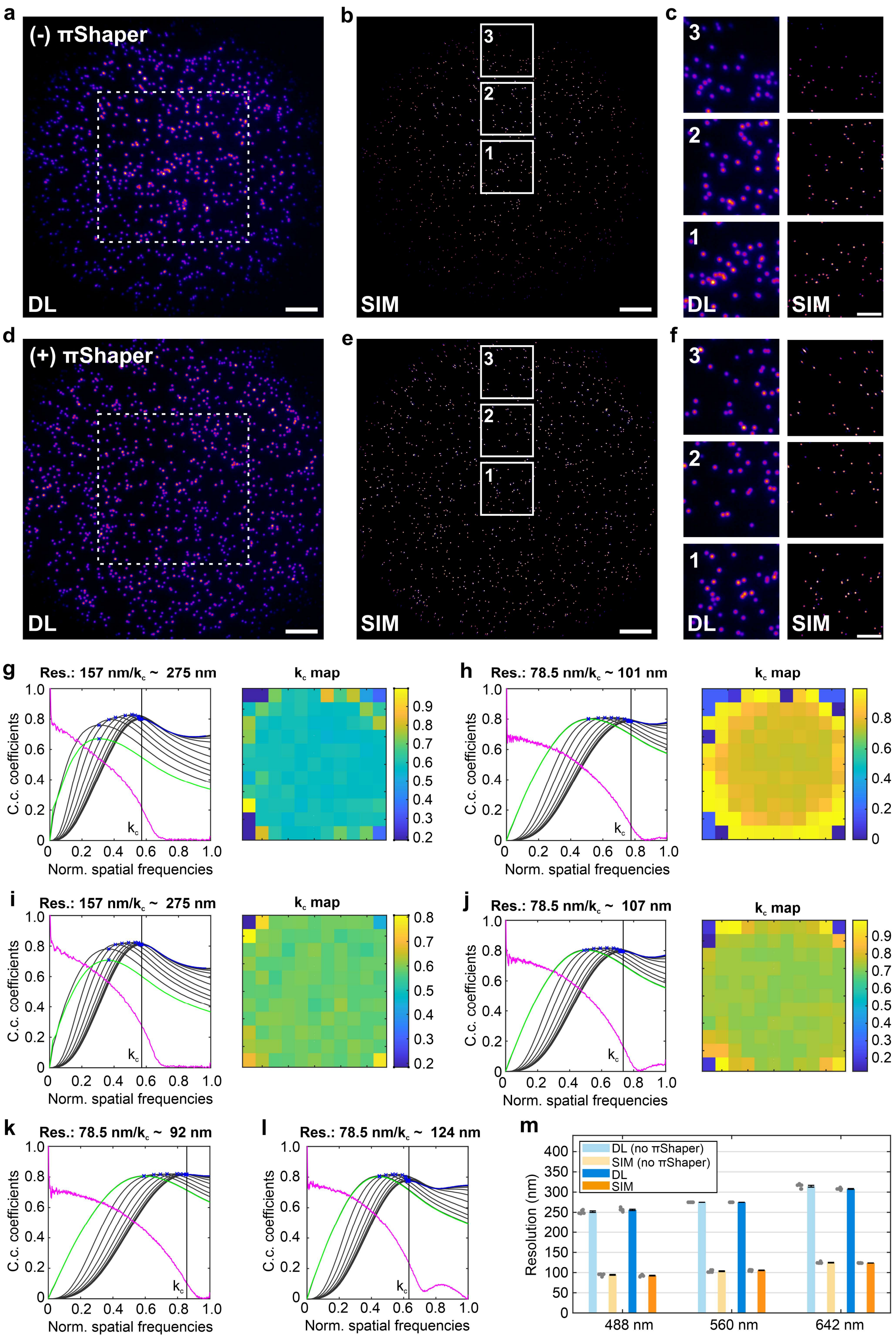

Supplementary Fig. 9: Beam shaping by πShaper does not degrade SIM super-resolution performance. a-f Comparison of pseudo diffraction limited (DL) and SIM images of 100 nm TetraSpec^{TM} microspheres without (**a-c**) and with πShaper (**d-f**) in the 560 nm channel. Dashed area in **a** and **d** shows field of view using image splitters. ROIs 1-3 in **b** and **e** are highlighted in **c** and **f** demonstrating SIM reconstructions from the center to the edge of

field of illumination. Scale bars: 2 µm in **a**-**b** and **d**-**e**, 1 µm in **c** and **f**. **g-j** Overall (left) and 11x11 tiled (right) decorrelation analysis of images in **a**-**c** without (**g**, **h**) and images in **d**-**f** with πShaper (**i**, **j**). Green line, decorrelation functions before high-pass filtering; magenta line, radial average of log of absolute value of Fourier transform; black lines, high-pass filtered decorrelation functions; blue crosses, all local maxima. Vertical black line, cut-off spatial frequency k_c, C.c., cross-correlation (details in methods section). **k-l** Decorrelation analysis of SIM images of the 488 nm channel (**k**) and 642 nm channel (**l**) of same TetraSpec^{TM} microsphere sample. **m** Comparison of spatial resolution with and without piShaper for DL and SIM reconstructions. Averaged resolution (bar plots) is based on decorrelation analysis on six independent sample positions. Scatter plots of individual estimates are added to each bar plot (grey dots). Error bars are representing standard error for each condition. Source data are provided as a Source Data file.

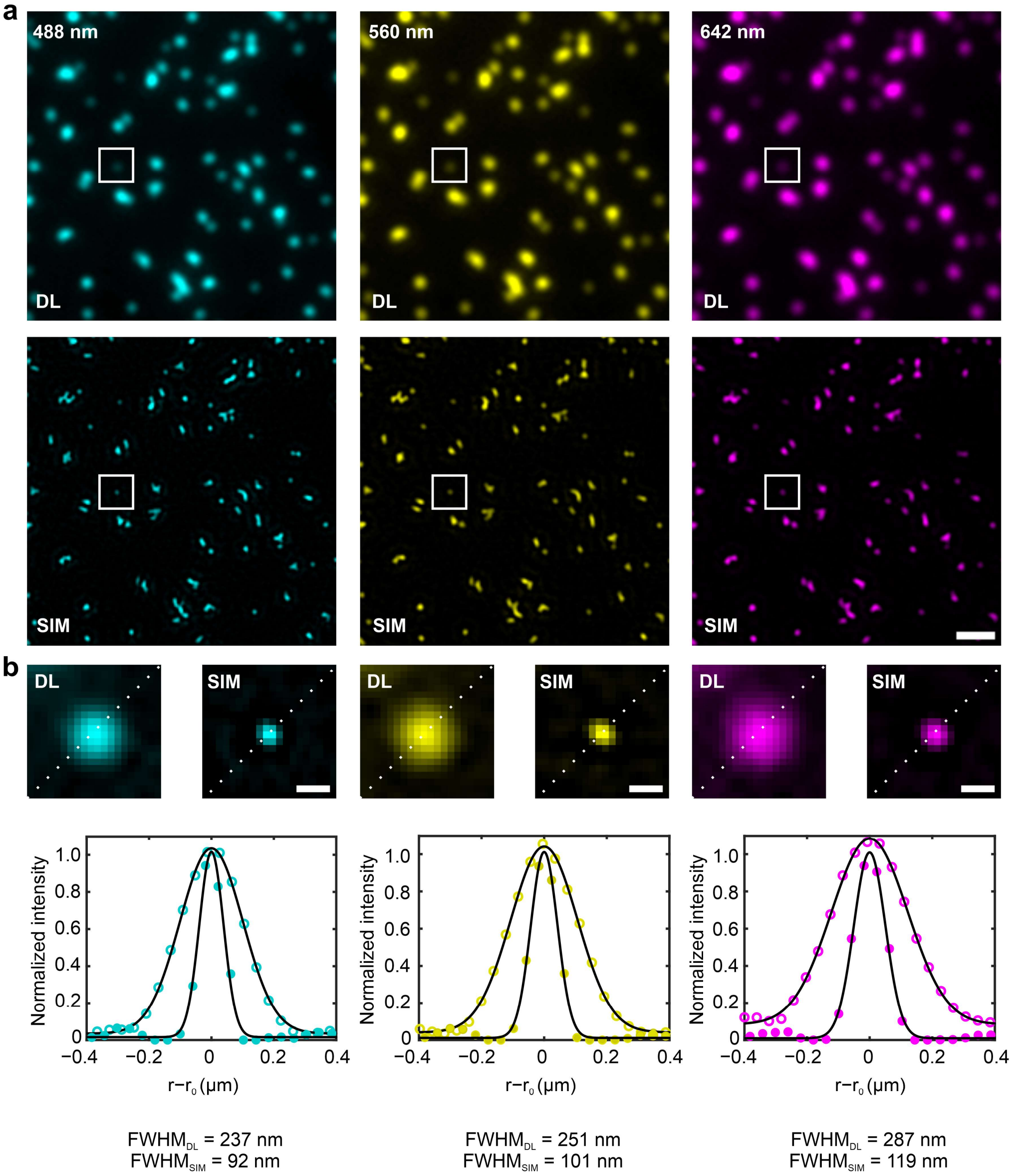

Supplementary Fig. 10: Effective PSF estimation by three-color SIM imaging of single fluorescent nanoparticles. a Diffraction-limited (DL) images (top) and deconvolved SIM images (bottom) of 100 nm TetraSpec™ microspheres in the three channels. DL images are synthetic images derived from averaging 9 SIM images. Scale bar: 1 µm. **b** Top: Zoomed image from ROI highlighted in **a** showing an individual nanoparticle by diffraction-limited images (DL) and after SIM deconvolution. Scale bar: 200 nm. Bottom: cross-sections and resolution determined as the FWHM (Gaussian fit) of the intensity distributions for all three channels. Open circles: DL cross-sections, closed circles: cross-sections from SIM reconstructions. Black lines: Gaussian fits. Source data are provided as a Source Data file.

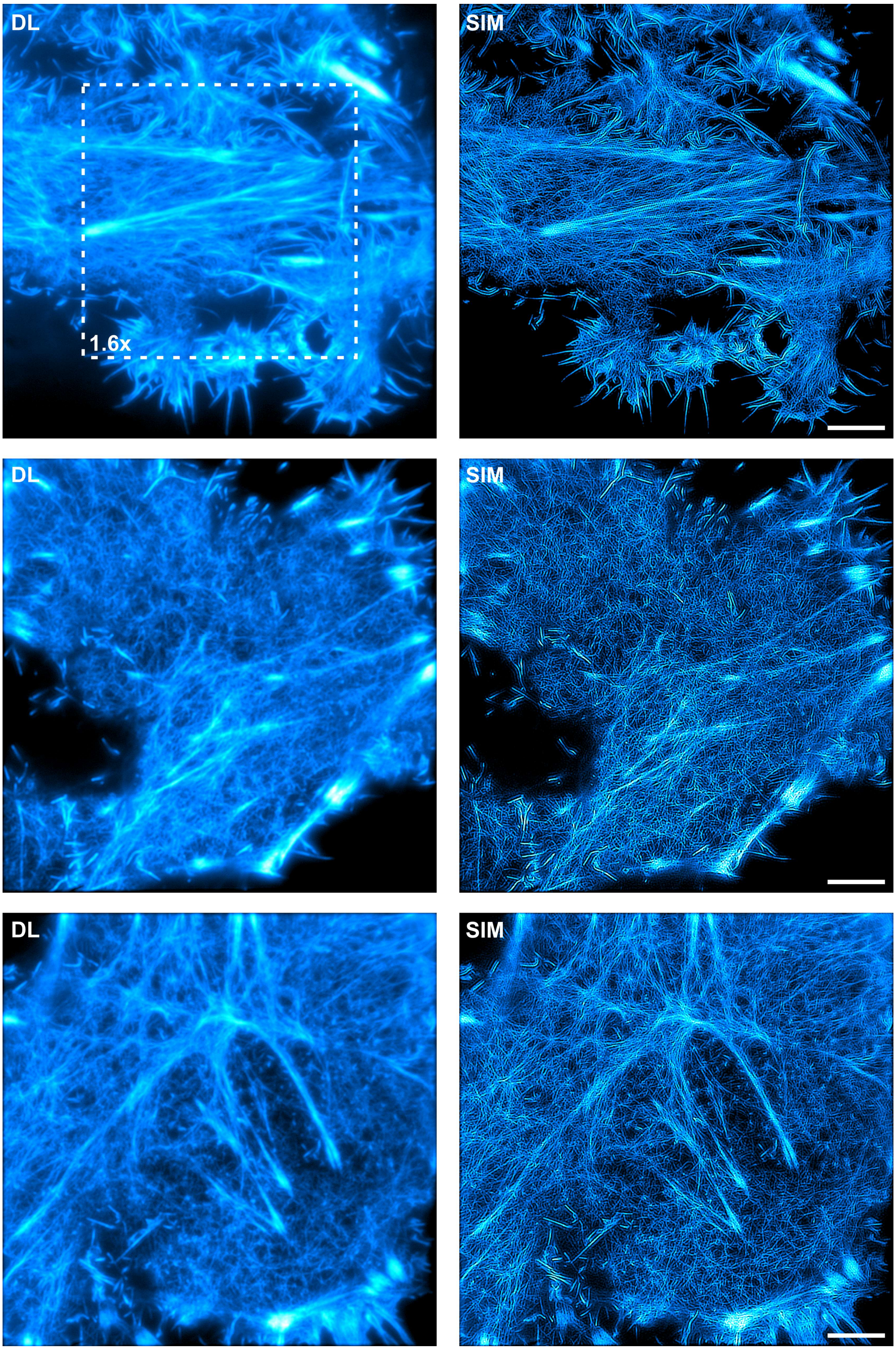

Supplementary Fig. 11: TIRF-SIM of the actin cortex at maximum field of view. Live HeLa-cells expressing LifeAct-SG were imaged without additional 1.6x magnification which is only required for single-molecule imaging. Smaller field of view including 1.6x magnification is indicated (dashed square). Diffraction limited pseudo-TIRF image (left) versus SIM reconstruction (right). All images were processed with gamma correction (parameter = 0.5) and unsharp masking (radius = 1 pxl, mask weight = 0.6). Three representative cells are shown. Scale bar: 5 µm.

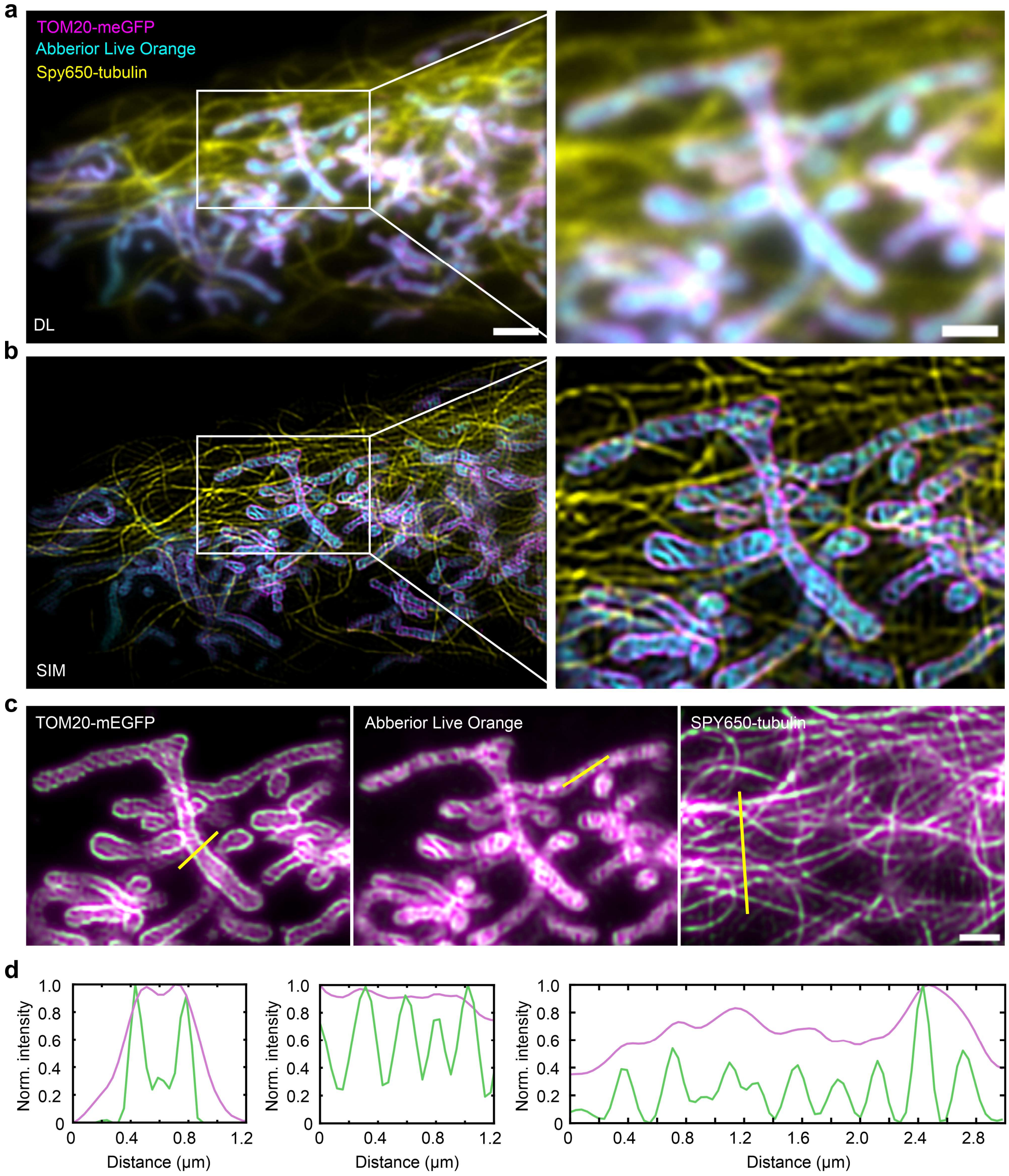

Supplementary Fig. 12: Fast three-color GI-SIM of HeLa-cells expressing TOM20-meGFP and labeled with Abberior Mito Orange and SPY650-tubulin. a, b Diffraction-limited (**a**) and SIM-deconvolved (**b**) three-color images showing an overview (left) and a zoomed ROI (right) highlighted by a white rectangle. Scale bars: 2 µm (left images) and 1 µm (right images). **c, d** Individual channels from right images of **a** (magenta) and **b** (green) and intensity cross-sections (**d**) along the indicated yellow lines in **c**. Source data are provided as a Source Data file.

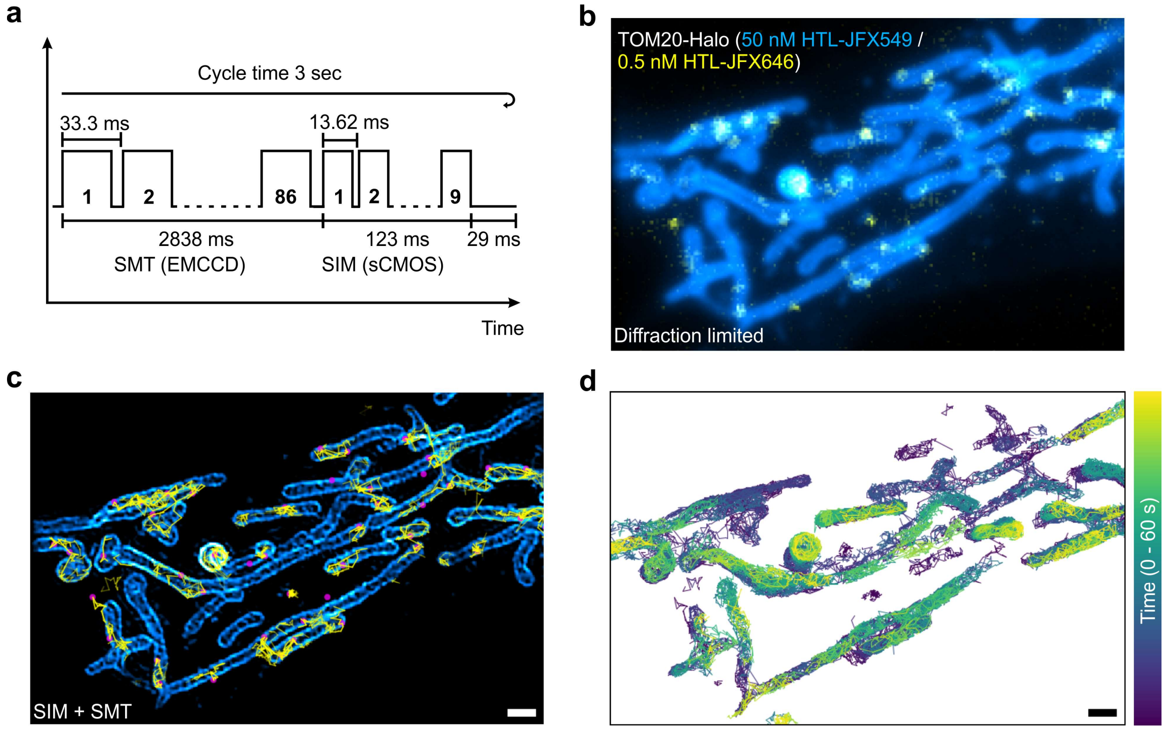

Supplementary Fig. 13: Combination of GI-SIM and SMT imaging in live mitochondria. **a** Diagram showing acquisition cycle of single-molecule imaging (86 frames) at 30 frames per second and subsequent acquisition of 9 SIM images with a total cycle time of 3 seconds. **b, c** Simultaneous SIM and SMT of TOM20-HaloTag in live cells by bulk labeling using 50 nM HTL-JFX549 (SIM channel) and substoichiometric labeling at 0.5 nM using HTL-JFX646 (SMT channel). Diffraction limited representation (**b**) versus processed images (**c**) using SIM reconstruction (cyan) and single-molecule localization and tracking. Magenta dots: single localizations, yellow lines: trajectories. **d** Color-coded trajectories of 5,400 consecutive frames. Scale bar: 1 µm.

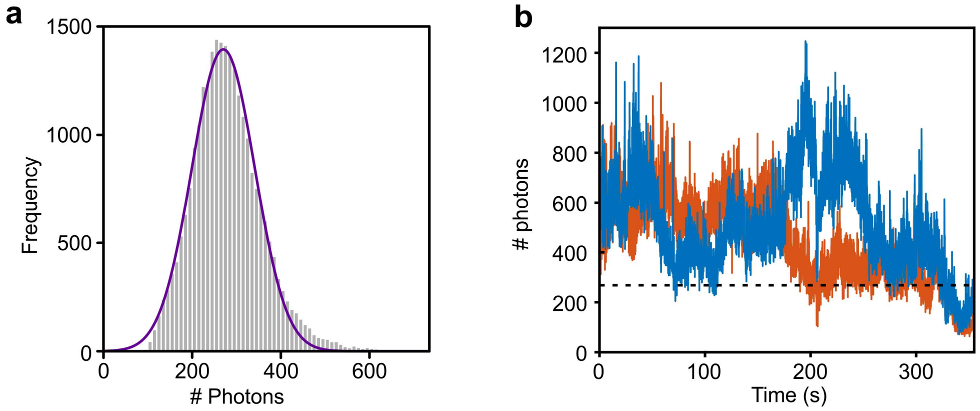

Supplementary Fig. 14: Temporal intensity-analysis of quasi-immobile TpoR signals during endocytosis. a Monomodal intensity distribution of mobile TpoR fraction representing monomeric intensity level as reference (cp. Supplementary Movie S16). After single-molecule localization and cluster-immobility filtering, mobile fraction was filtered for $\mathrm{SNR} > 22$ dB and tracked. Intensity histogram from trajectories longer than 10 frames was fitted with a single Gaussian function resulting in a peak at (270 ± 2) photons (n = 25.633 localizations). **b** Single-particle intensity analysis of immobile TpoR signals for two endocytosis events shown in Fig. 5j (orange: ROI 1, blue: ROI 2). Here, immobile TpoR in endosomes were identified by cluster/immobility filtering to obtain single-particle intensities in units of photons. Dashed line indicates intensity level of monomeric TpoR from **a**. Source data are provided as a Source Data file.

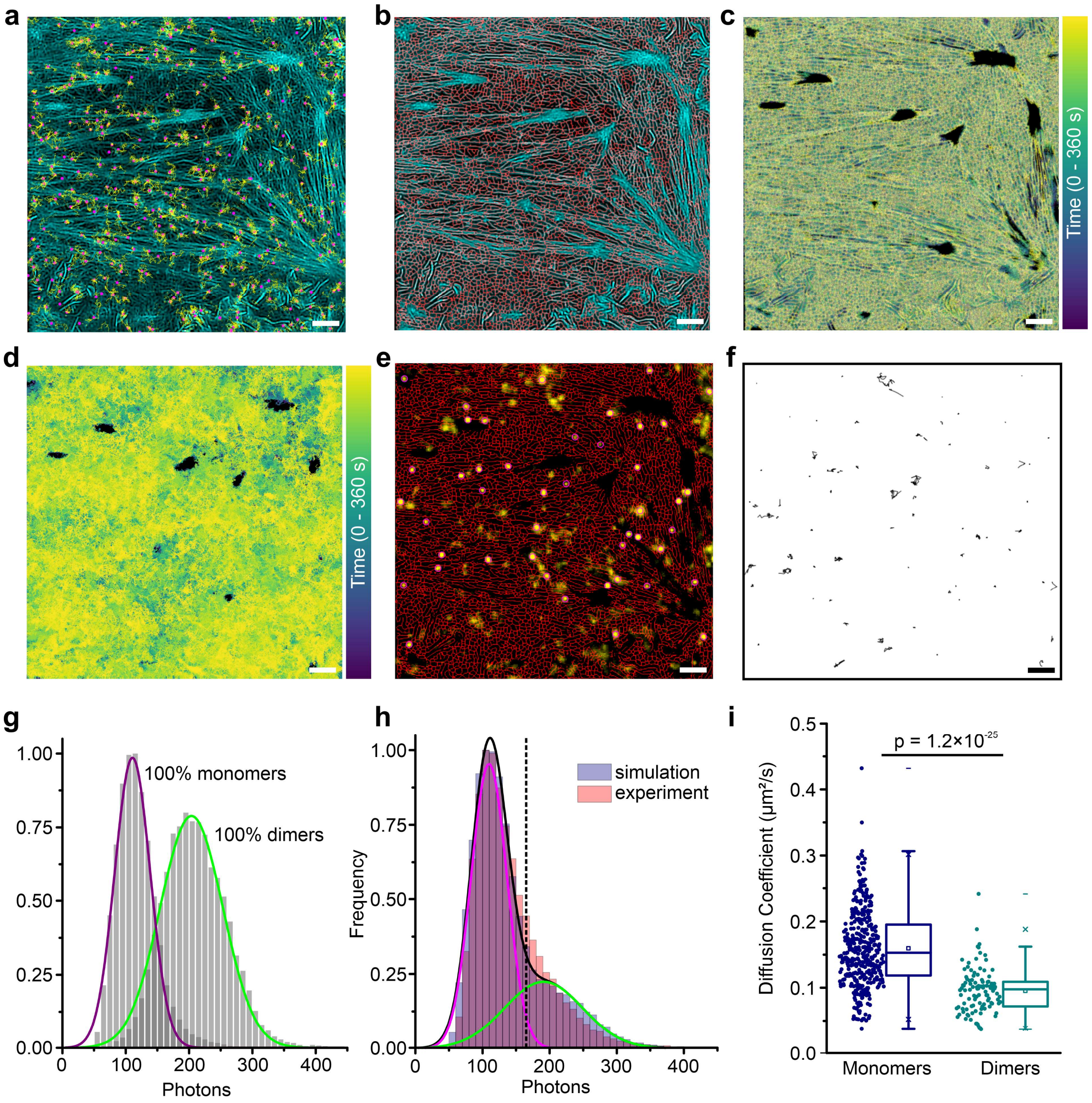

Supplementary Fig. 15: Correlative TIRF-SIM and SMT of actin and TpoR dynamics after stimulation with thrombopoietin. a Simultaneous SIM imaging of LifeAct-SG (cyan) and SMI of TpoR in the presence of TPO (yellow trajectories). A gamma correction (parameter=0.5) as well as unsharp masking (radius = 1 pxl, mask weight = 0.6) was applied to actin channel. **b** WEKA segmentation (red) of the LifeAct-SG SIM image (cyan) shown in **a**. Scale bar: 2 µm. **c** Color-coded overlay of segmented meshwork structure from 120 consecutive SIM images. Scale bar: 2 µm. **d** TpoR trajectories from 10,800 consecutive frames (360 s) color-coded for time (n = 56.353 trajectories). Scale bar: 2 µm. **e** Segmentation of actin cortex (red) overlaid with slow moving fraction of TpoR channel (mean intensity projection of TpoR from 90 consecutive frames). Magenta circles mark tracked TpoR signals by TrackMate. Scale bar: 2 µm. **f** Trajectory map of slow-moving TpoR signals with a trajectory length > 30 s (n = 81 trajectories). Scale bar: 2 µm. **g-h** Simulation of TpoR diffusion (300 frames) matching the experimental result shown in Figure **6 e-f**. **g** Intensity distribution of modeled 100% monomers (5.84×10^4 localizations) or dimers (1.93×10^4 localizations) with a matching particle density of 0.6 µm^{-2} (monomers) or 0.08 µm^{-2} (dimers), respectively. **h** Intensity distributions of modeled 13% dimerization level (5.92×10^4 localizations) versus experimental intensity distribution from Figure **6f** (3.43×10^4 localizations). Dashed line indicates intensity threshold to classify monomers (< 165 photons) and dimers (> 165 photons) for diffusion analysis in **i**. **i** Distributions of diffusion coefficients on single-trajectory level for filtered monomers (395 trajectories) and dimers (108 trajectories) obtained from simulated images. Modelled diffusion coefficients: Monomers: 0.16 µm^2/s, dimers: 0.10

μm^2/s. Further simulation parameters are listed in Supplementary Table 1. Box plot indicates data distribution of the second and third quartiles (box), median (line), mean (square), and 1.5x interquartile range (whiskers). Statistics for **i** were performed using two-sample Kolmogorov–Smirnov test. Source data are provided as a Source Data file.
