# Supplementary material for: Correlative single-molecule and structured illumination microscopy of fast dynamics at the plasma membrane
Source: Nat Commun. 2024 Jul 10;15:5813. doi: 10.1038/s41467-024-49876-9 (PMC11236984; doi:10.1038/s41467-024-49876-9)
Supplement: Supplementary file 2 — Reporting Summary [file 41467_2024_49876_MOESM2_ESM.pdf]

nature portfolio

Corresponding author(s): Jacob Piehler & Rainer Kurre

Last updated by author(s): Jun 18, 2024

Reporting Summary

Nature Portfolio wishes to improve the reproducibility of the work that we publish. This form provides structure for consistency and transparency in reporting. For further information on Nature Portfolio policies, see our Editorial Policies and the Editorial Policy Checklist.

Statistics

For all statistical analyses, confirm that the following items are present in the figure legend, table legend, main text, or Methods section.

n/a	Confirmed	
☐	☒	The exact sample size (*n*) for each experimental group/condition, given as a discrete number and unit of measurement
☐	☒	A statement on whether measurements were taken from distinct samples or whether the same sample was measured repeatedly
☒	☐	The statistical test(s) used AND whether they are one- or two-sided<br>*Only common tests should be described solely by name; describe more complex techniques in the Methods section.*
☒	☐	A description of all covariates tested
☒	☐	A description of any assumptions or corrections, such as tests of normality and adjustment for multiple comparisons
☐	☒	A full description of the statistical parameters including central tendency (e.g. means) or other basic estimates (e.g. regression coefficient) AND variation (e.g. standard deviation) or associated estimates of uncertainty (e.g. confidence intervals)
☐	☒	For null hypothesis testing, the test statistic (e.g. *F*, *t*, *r*) with confidence intervals, effect sizes, degrees of freedom and *P* value noted<br>*Give P values as exact values whenever suitable.*
☒	☐	For Bayesian analysis, information on the choice of priors and Markov chain Monte Carlo settings
☒	☐	For hierarchical and complex designs, identification of the appropriate level for tests and full reporting of outcomes
☐	☒	Estimates of effect sizes (e.g. Cohen's *d*, Pearson's *r*), indicating how they were calculated

Our web collection on statistics for biologists contains articles on many of the points above.

Software and code

Policy information about availability of computer code

Data collection	Micro-Manager 2.0.1 (www.micro-manager.org), MetroCon 4.1 (Forth Dimension Displays), LabView 6 (National Instruments)
Data analysis	Matlab R2013a, R2020b & R2022b (MathWorks), The Matlab R2013a software SLIMfast (Version 16h) is available via https://doi.org/10.5281/zenodo.3588413 and the Matlab R2020b simulation code SPT_Simulator V1.0.0 can be found under https://doi.org/10.5281/zenodo.11203941. The Matlab R2022b script for FRET efficiency analysis accompanied with a demo data set is provided as Supplementary Software with this paper, decorrelation analysis (Matlab code from Descloux et al., Nat. Meth., 2019), FIJI (Fiji Is Just ImageJ 2.14.0/1.54f/1.53t, Java 1.8.0_172 [64-bit]), fairSIM in FIJI (git build id: 6d03793bab), TrackMate 7.10.2. in FIJI, PICASSO 0.6.1, WEKA segmentation v3.3.4i n FIJI, OriginPro 9.0, Bitplane Imaris 9.5.

For manuscripts utilizing custom algorithms or software that are central to the research but not yet described in published literature, software must be made available to editors and reviewers. We strongly encourage code deposition in a community repository (e.g. GitHub). See the Nature Portfolio guidelines for submitting code & software for further information.

Data

Policy information about availability of data

All manuscripts must include a data availability statement. This statement should provide the following information, where applicable:

- Accession codes, unique identifiers, or web links for publicly available datasets
- A description of any restrictions on data availability
- For clinical datasets or third party data, please ensure that the statement adheres to our policy

The microscopy data generated in this study have been deposited in the OsnaData repository under accession code: https://doi.org/10.26249/FK2/V8HWWH. All plasmids from this study were deposited at Addgene (https://www.addgene.org/, IDs 222944-222949). Source data are provided with this paper.

Research involving human participants, their data, or biological material

Policy information about studies with human participants or human data. See also policy information about sex, gender (identity/presentation), and sexual orientation and race, ethnicity and racism.

Reporting on sex and gender	Not applicable
Reporting on race, ethnicity, or other socially relevant groupings	Not applicable
Population characteristics	Not applicable
Recruitment	Not applicable
Ethics oversight	Not applicable

Note that full information on the approval of the study protocol must also be provided in the manuscript.

Field-specific reporting

Please select the one below that is the best fit for your research. If you are not sure, read the appropriate sections before making your selection.

☒ Life sciences ☐ Behavioural & social sciences ☐ Ecological, evolutionary & environmental sciences

For a reference copy of the document with all sections, see nature.com/documents/nr-reporting-summary-flat.pdf

Life sciences study design

All studies must disclose on these points even when the disclosure is negative.

Sample size	The sample size of the photon histograms in Fig. 1g, Fig. 2d, f and Fig. 6f and Supplementary Figs. 3d, 3g, 4b, 4d, 4f, 4h, 5g, 6d, 6f, 6j, 6k, 14a, 15g and 15h was chosen to obtain smooth Gaussian distributions with small statistical errors, as indicated. Typical sizes are between 1E5-1E6 single molecule signals per peak for reHaloTagF-PAINT experiments (Fig. 1g and Supplementary Fig. 3d) and 5E3-5E4 for single molecule stoichiometry experiments (all other figures mentioned). For single-molecule colocalization and single-molecule FRET experiments (Fig. 3), sample sizes were chosen to cover both statistical (approx. 20,000 FRET signals) and biological variations (18 cells), with the aim of obtaining a smooth histogram of FRET efficiencies for robust Gaussian fitting (Fig. 3i and Supplementary Fig. 7b-d). In the case of the long-term single molecule tracking (Fig. 2, Fig. 5d, Supplementary Figs. 8 and 15d) and the correlative actin cortex and receptor diffusion experiments (Figs. 5 and 6, Supplementary Figs. 14 and 15a, e), representative cells are mainly shown to demonstrate the capabilities of the microscopy technique. Each experiment was repeated at least twice using independent experimental samples.
Data exclusions	In case of single-molecule stoichiometry, tracking, and FRET assays, single cells were only excluded when single-molecule density was too high for robust tracking and FRET analysis or signal-to-noise was too low. Structured illumination microscopy data showing too low signal-to-noise for robust reconstruction with low level of artefacts were also exluded. Data filtering is described in detail in the methods section.
Replication	When required, experiments were independently repeated and reproducibility was validated.
Randomization	Not applicable.
Blinding	Not applicable.

Behavioural & social sciences study design

All studies must disclose on these points even when the disclosure is negative.

Study description	*Briefly describe the study type including whether data are quantitative, qualitative, or mixed-methods (e.g. qualitative cross-sectional, quantitative experimental, mixed-methods case study).*
Research sample	*State the research sample (e.g. Harvard university undergraduates, villagers in rural India) and provide relevant demographic information (e.g. age, sex) and indicate whether the sample is representative. Provide a rationale for the study sample chosen. For studies involving existing datasets, please describe the dataset and source.*
Sampling strategy	*Describe the sampling procedure (e.g. random, snowball, stratified, convenience). Describe the statistical methods that were used to predetermine sample size OR if no sample-size calculation was performed, describe how sample sizes were chosen and provide a rationale for why these sample sizes are sufficient. For qualitative data, please indicate whether data saturation was considered, and what criteria were used to decide that no further sampling was needed.*
Data collection	*Provide details about the data collection procedure, including the instruments or devices used to record the data (e.g. pen and paper, computer, eye tracker, video or audio equipment) whether anyone was present besides the participant(s) and the researcher, and whether the researcher was blind to experimental condition and/or the study hypothesis during data collection.*
Timing	*Indicate the start and stop dates of data collection. If there is a gap between collection periods, state the dates for each sample cohort.*
Data exclusions	*If no data were excluded from the analyses, state so OR if data were excluded, provide the exact number of exclusions and the rationale behind them, indicating whether exclusion criteria were pre-established.*
Non-participation	*State how many participants dropped out/declined participation and the reason(s) given OR provide response rate OR state that no participants dropped out/declined participation.*
Randomization	*If participants were not allocated into experimental groups, state so OR describe how participants were allocated to groups, and if allocation was not random, describe how covariates were controlled.*

Ecological, evolutionary & environmental sciences study design

All studies must disclose on these points even when the disclosure is negative.

Study description	*Briefly describe the study. For quantitative data include treatment factors and interactions, design structure (e.g. factorial, nested, hierarchical), nature and number of experimental units and replicates.*
Research sample	*Describe the research sample (e.g. a group of tagged Passer domesticus, all Stenocereus thurberi within Organ Pipe Cactus National Monument), and provide a rationale for the sample choice. When relevant, describe the organism taxa, source, sex, age range and any manipulations. State what population the sample is meant to represent when applicable. For studies involving existing datasets, describe the data and its source.*
Sampling strategy	*Note the sampling procedure. Describe the statistical methods that were used to predetermine sample size OR if no sample-size calculation was performed, describe how sample sizes were chosen and provide a rationale for why these sample sizes are sufficient.*
Data collection	*Describe the data collection procedure, including who recorded the data and how.*
Timing and spatial scale	*Indicate the start and stop dates of data collection, noting the frequency and periodicity of sampling and providing a rationale for these choices. If there is a gap between collection periods, state the dates for each sample cohort. Specify the spatial scale from which the data are taken*
Data exclusions	*If no data were excluded from the analyses, state so OR if data were excluded, describe the exclusions and the rationale behind them, indicating whether exclusion criteria were pre-established.*
Reproducibility	*Describe the measures taken to verify the reproducibility of experimental findings. For each experiment, note whether any attempts to repeat the experiment failed OR state that all attempts to repeat the experiment were successful.*
Randomization	*Describe how samples/organisms/participants were allocated into groups. If allocation was not random, describe how covariates were controlled. If this is not relevant to your study, explain why.*
Blinding	*Describe the extent of blinding used during data acquisition and analysis. If blinding was not possible, describe why OR explain why blinding was not relevant to your study.*

Did the study involve field work? ☐ Yes ☒ No

Reporting for specific materials, systems and methods

We require information from authors about some types of materials, experimental systems and methods used in many studies. Here, indicate whether each material, system or method listed is relevant to your study. If you are not sure if a list item applies to your research, read the appropriate section before selecting a response.

Materials & experimental systems

n/a	Involved in the study	
☐	☒	Antibodies
☐	☒	Eukaryotic cell lines
☒	☐	Palaeontology and archaeology
☒	☐	Animals and other organisms
☒	☐	Clinical data
☒	☐	Dual use research of concern
☒	☐	Plants

Methods

n/a	Involved in the study	
☒	☐	ChIP-seq
☒	☐	Flow cytometry
☒	☐	MRI-based neuroimaging

Antibodies

Antibodies used	Nanobodies: Anti-GFP, tdALFAnb (original name: NbALFA)
Validation	Anti-GFP was validated in doi.org/10.1016/j.crmeth.2022.100165<br>NbALFA was validated in doi.org/10.1038/s41467-019-12301-7

Eukaryotic cell lines

Policy information about cell lines and Sex and Gender in Research

Cell line source(s)	HeLa cell line from DSMZ German Collection of Microorganisms and Cell Cultures GmbH (DSMZ No.: ACC 57)
Authentication	HeLa cell line (German Collection of Microorganisms and Cell Cultures GmbH) was guaranteed by the suppliers and no additional authentication was performed by the authors of this study.
Mycoplasma contamination	All cell lines have been tested negative.
Commonly misidentified lines<br>(See ICLAC register)	No commonly misidentified lines were used in this study.
