# Supplementary material for: Correlative single-molecule and structured illumination microscopy of fast dynamics at the plasma membrane
Source: Nat Commun. 2024 Jul 10;15:5813. doi: 10.1038/s41467-024-49876-9 (PMC11236984; doi:10.1038/s41467-024-49876-9)
Supplement: Supplementary file 4 — Description of additional supplementary files [file 41467_2024_49876_MOESM4_ESM.pdf]

Supplementary Movies

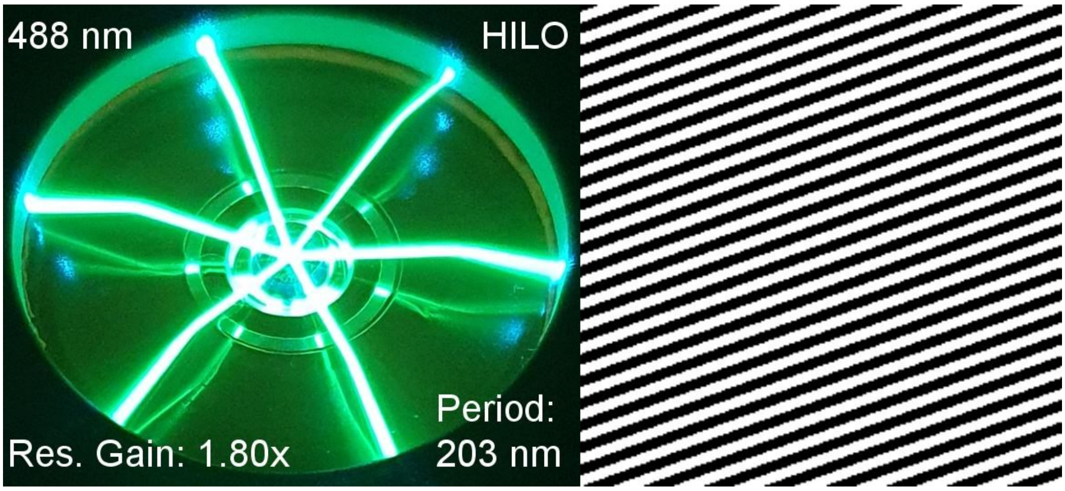

Supplementary Movie 1. Illustration of binary phase grating switching within single camera exposures and different illumination conditions. Here, we were illuminating a dye solution (volume: 1 ml) containing fluorescein, Texas Red and ATTO 655 at approx. 1-2 µM in PBS. Left side is showing pictures of our open custom-build sample chamber on the microscope stage. Depending on the grating period, laser is entering dye solution (HILO), propagating horizontally with respect to cover slip (grazing incidence at critical angle) or is totally reflected (TIR). Movie is showing all three channels (488 nm, 560 nm, and 642 nm) and referring to theoretical resolution gain factor for 2D-SIM as well the period of structured illumination in the focal plane of the objective.

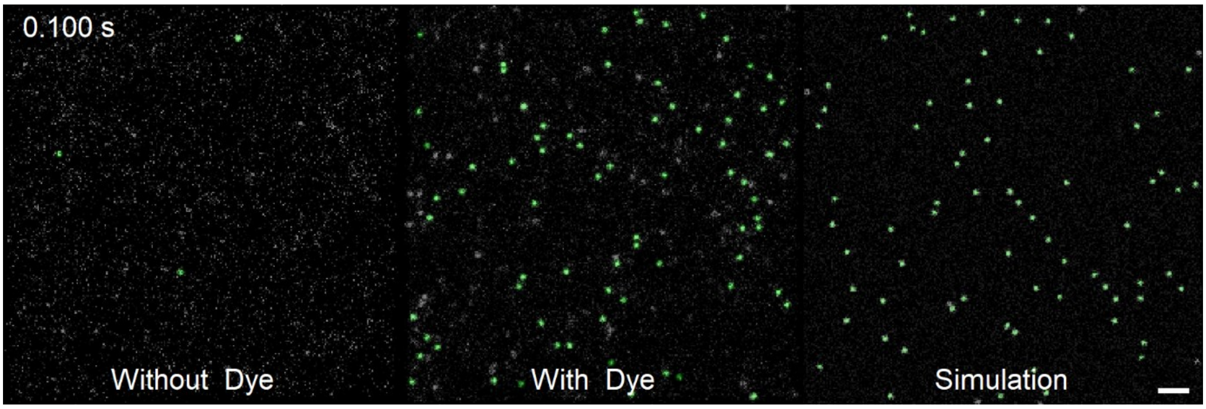

Supplementary Movie 2. PAINTing of immobilized reHaloTags by HaloTag ligands conjugated to MaP555 (HTL-MaP555). Tris-NTA surface loaded with reHaloTag in the absence (left) and presence (middle) of HTL-MaP555 in comparison to simulated data. Tracked localizations of immobile trajectories longer than 2 frames are highlighted in green. Scale bar: 2 µm.

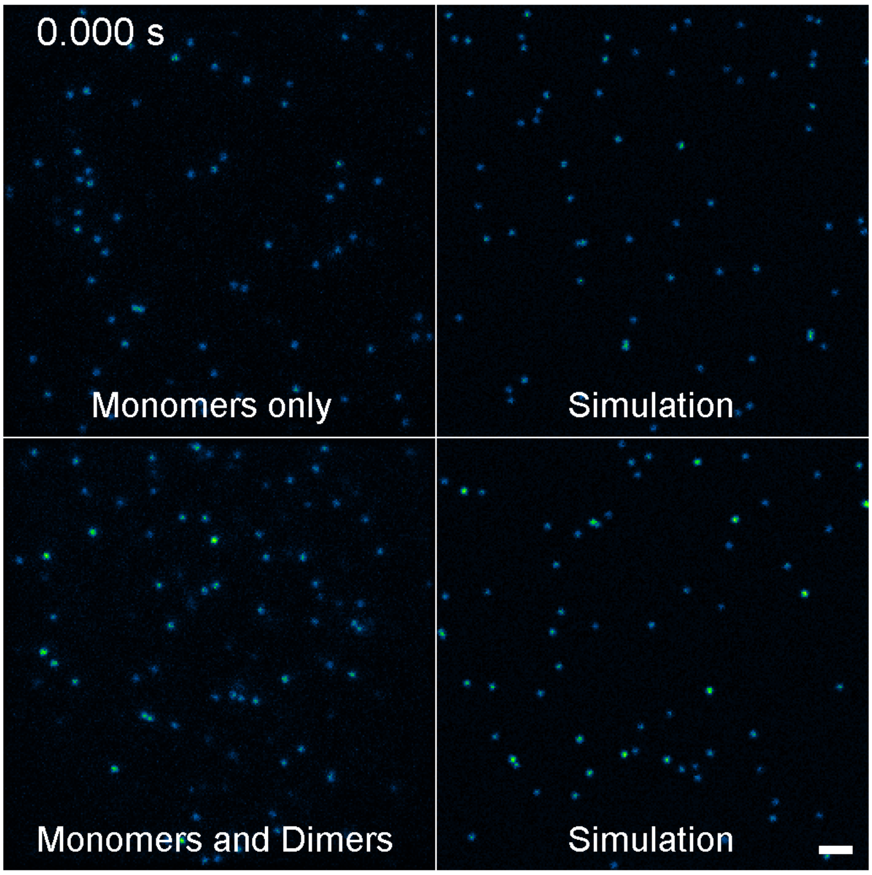

Supplementary Movie 3. Receptor stoichiometry by intensity analysis. ALFA-mXFP-TpoR labeled with ^{AT643}EN in the absence (top left) and presence (bottom left) of dimerizer tdALFAnb as well as matching simulations. Scale bar: 2 µm.

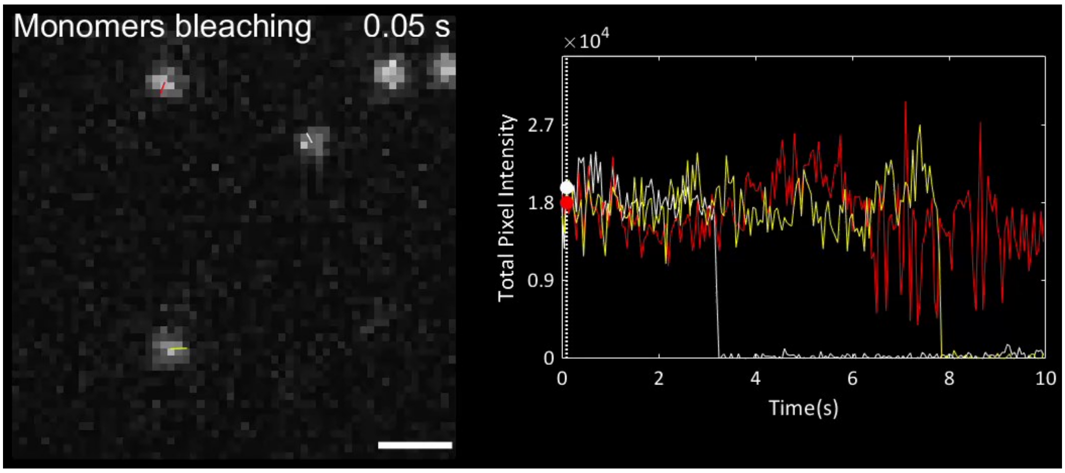

Supplementary Movie 4. Photobleaching of monomeric ^{AT643}EN-labeled ALFA-mXFP-TpoR in the absence of tdALFAnb. Scale bar: 1 µm.

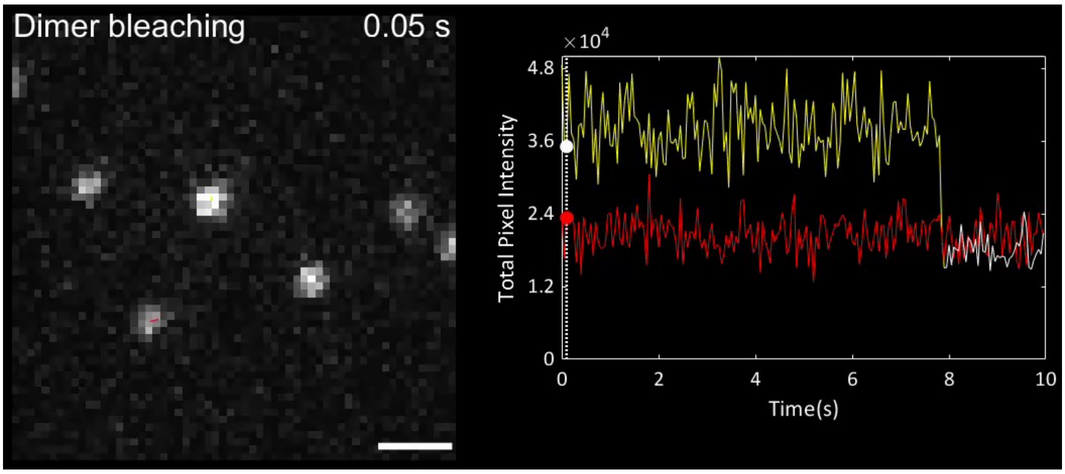

Supplementary Movie 5. Photobleaching of dimeric ^{AT643}EN-labeled ALFA-mXFP-TpoR in the presence of tdALFAnb. Scale bar: 1 µm.

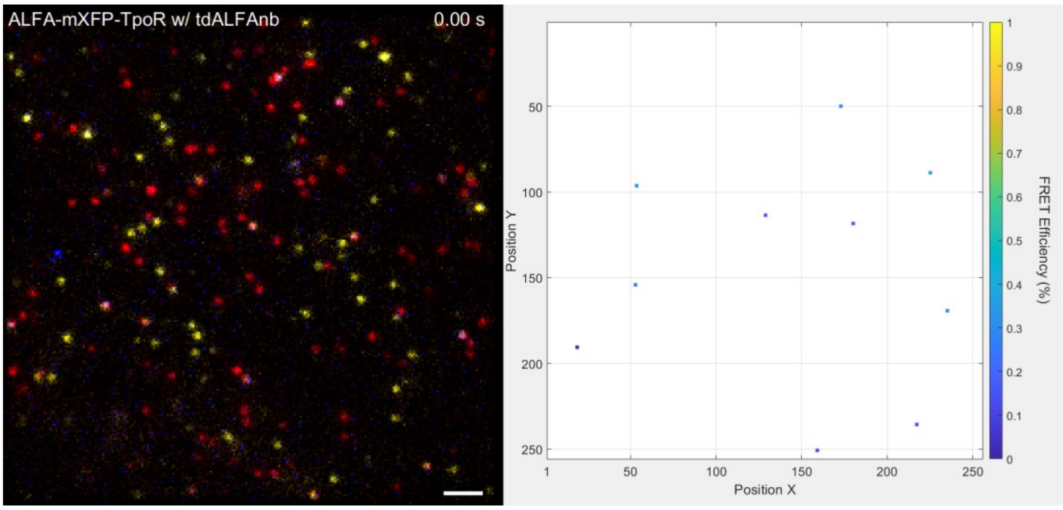

Supplementary Movie 6. Single-molecule ALEX-FRET detects TpoR dimers in the plasma membrane of live cells. Left: Overlay of raw data channels: Donor (yellow), acceptor (red) and FRET (blue). Single dimers are clearly visible as diffusing white signals. Scale bar: 2 µm. Right: Frame-by-frame detected FRET signals color-coded with its FRET efficiency.

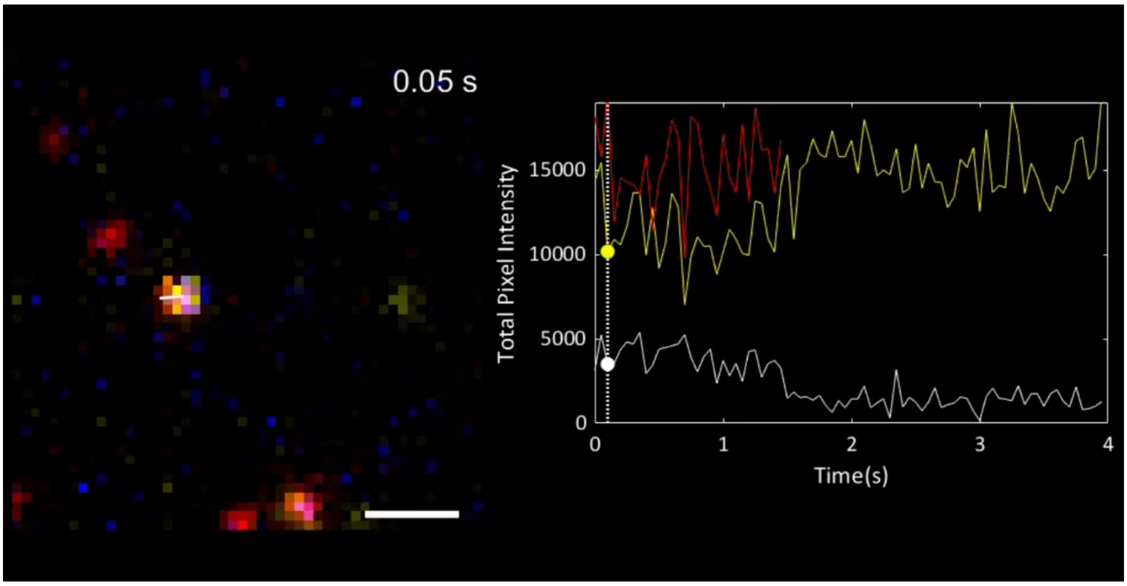

Supplementary Movie 7. Co-localization and co-tracking of donor (yellow), acceptor (red) and FRET channel (blue) on single complex level showing an acceptor bleaching event. Scale bar: 1 µm.

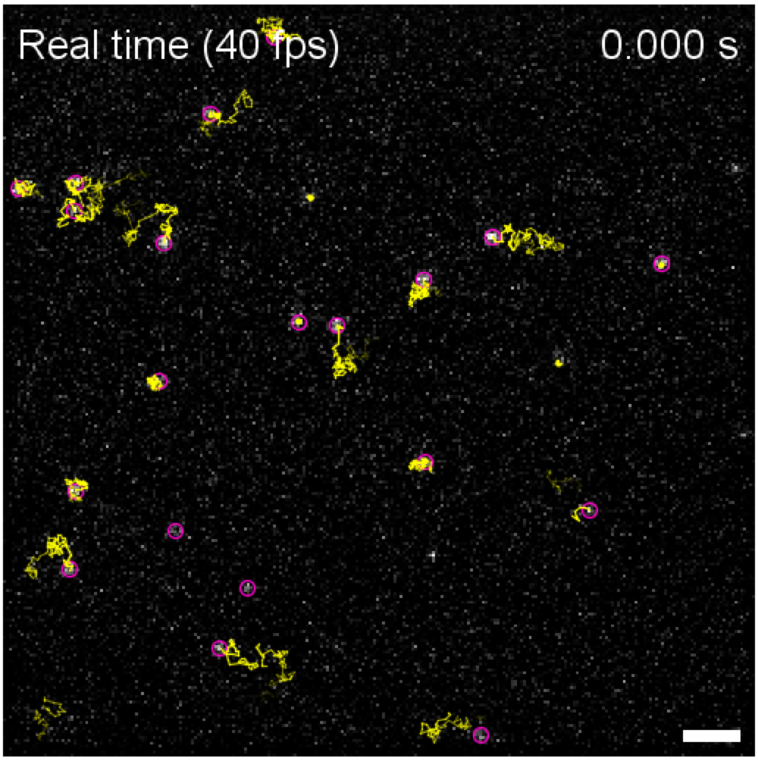

Supplementary Movie 8. Long-term single-molecule tracking at low receptor density. TpoR N-terminally fused to a non-fluorescent monomeric EGFP (mXFP) was expressed in HeLa cells and labeled with anti-GFP nanobodies conjugated to ATTO 643. A typical cell was imaged at 40 frames per second for 625 s (25,000 frames). Scale bar: 2 µm.

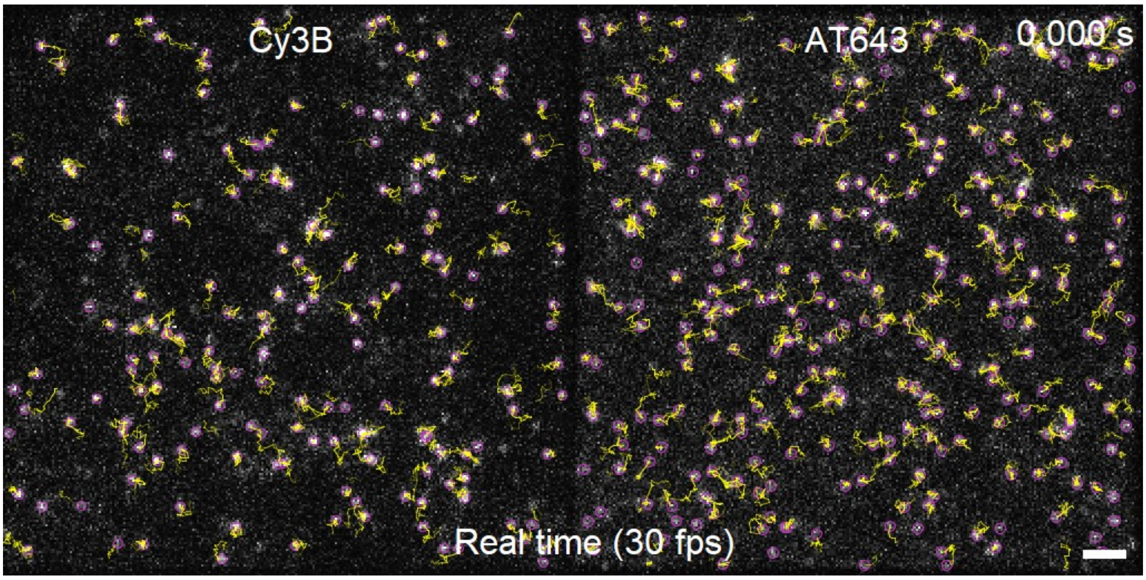

Supplementary Movie 9. Long-term dual-color single-molecule tracking at high receptor density. TpoR N-terminally fused to a non-fluorescent monomeric EGFP (mXFP) was expressed in HeLa cells and labeled at equimolar concentrations with anti-GFP nanobodies conjugated to Cy3B and ATTO 643, respectively. Simultaneous dual-color single-molecule tracking at 30 frames per second for 300 s (9,000 frames). Scale bar: 2 µm.

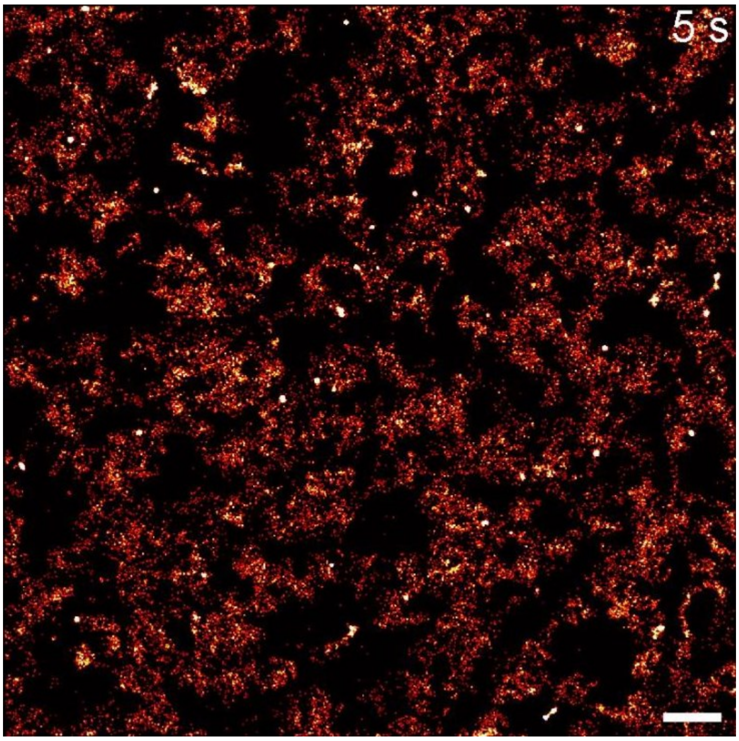

Supplementary Movie 10. Time-lapse localization map reveals receptor accessibility and endosome dynamics. TpoR N-terminally fused to a non-fluorescent monomeric EGFP (mXFP) was expressed in HeLa cells and labeled with anti-GFP nanobodies conjugated to ATTO 643. Single-molecule localizations of 150 consecutive frames (5 s) were binned and rendered with a moving window based on a time interval of 30 frames (1 s). Scale bar: 2 µm.

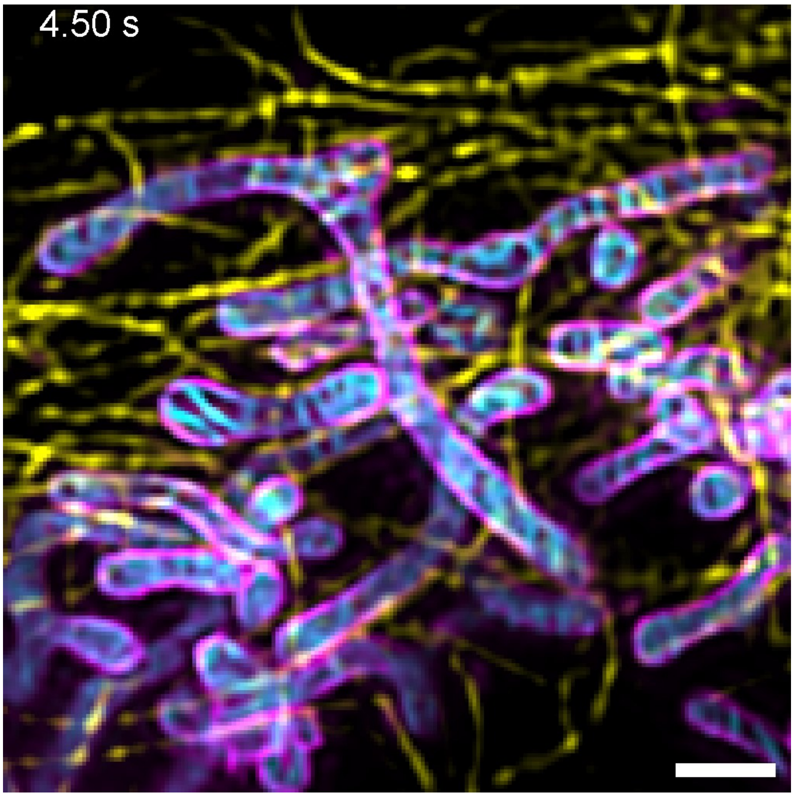

Supplementary Movie 11. Three-color SIM imaging of mitochondrial membrane dynamics within the microtubule network. GI-SIM of HeLa-cells expressing TOM20-meGFP (magenta) and labeled with Abberior Mito Orange (cyan) and SPY650-tubulin (yellow). Scale bar: 1 µm.

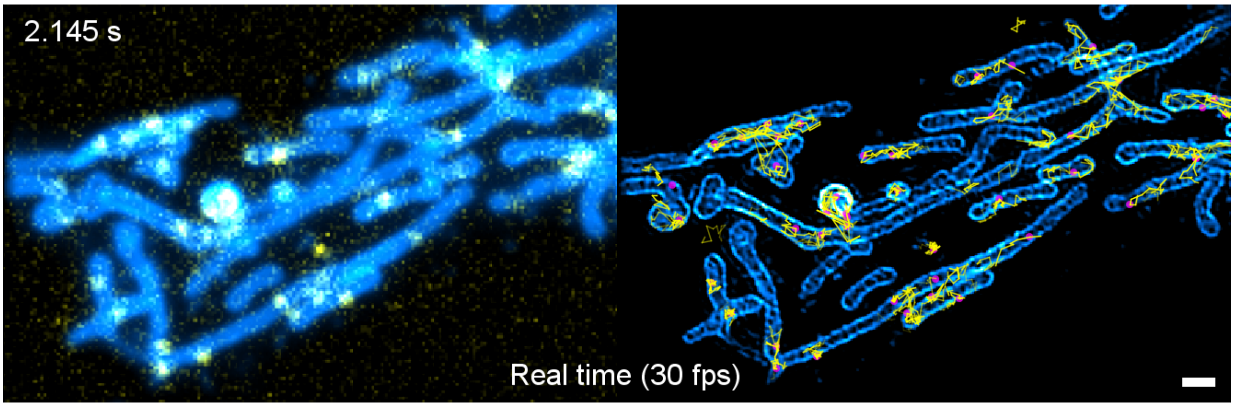

Supplementary Movie 12. Simultaneous SMT and SIM imaging of TOM20-HaloTag in live cells. Bulk labeling using 50 nM HTL-JFX549 (SIM channel) and substoichiometric labeling at 0.5 nM by HTL-JFX646 (SMT channel). By cycling between real-time single-molecule imaging at 30 Hz and short SIM acquisitions every three seconds, we achieved quasi-simultaneous SIM and SMT imaging in live cells. Diffraction limited representation (left) versus processed images showing SIM reconstruction (cyan) and single-molecule localization (magenta) and tracking (yellow) (right). Scale bar: 1 µm.

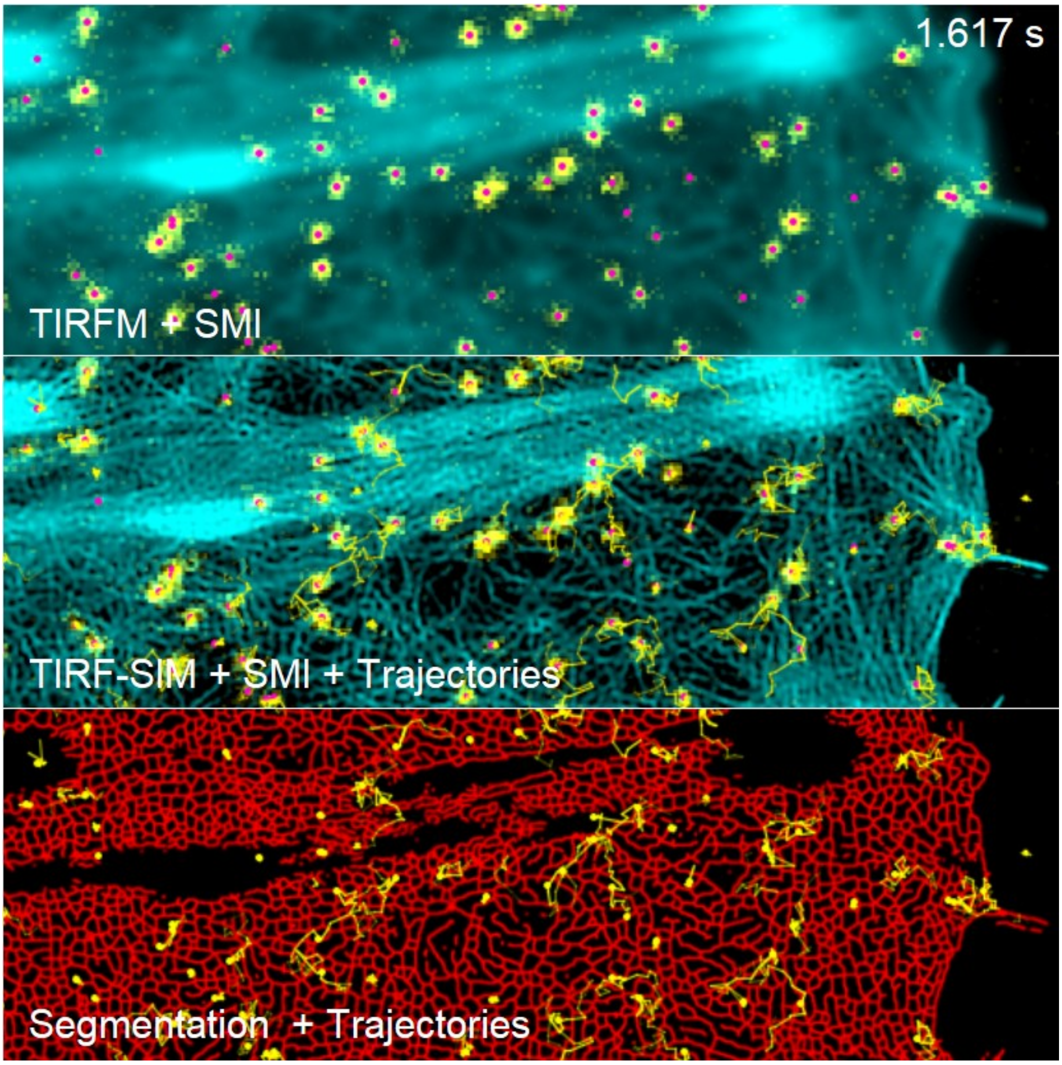

Supplementary Movie 13. Dynamics of the cortical cytoskeleton and Tpo receptors in the plasma membrane captured by TIRF structured illumination microscopy. TpoR N-terminally fused to a non-fluorescent monomeric EGFP (mXFP) was expressed in HeLa cells and labeled with anti-GFP nanobodies conjugated to ATTO 643. Actin cytoskeleton was imaged by LifeAct-HaloTag/JFX549. Cycling between real-time single-molecule imaging at 30 Hz and short SIM acquisitions every three seconds. Scale bar: 2 µm.

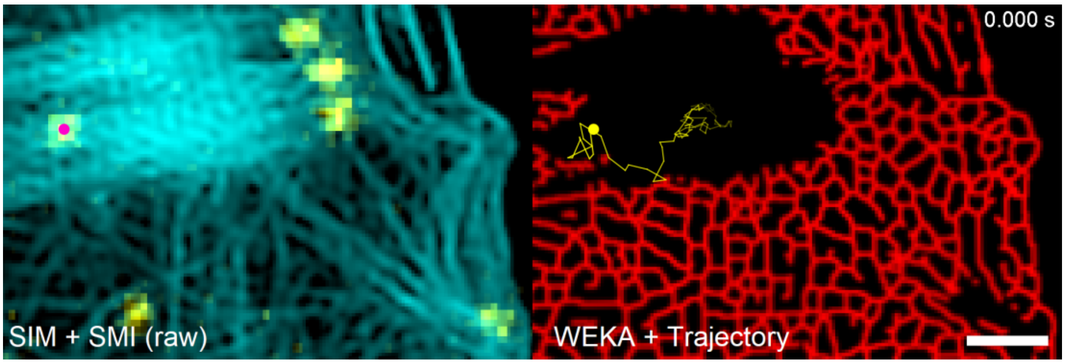

Supplementary Movie 14. Diffusion of TpoR in the context of clustered and corralled regions of the cortical cytoskeleton. Scale bar: 1 µm.

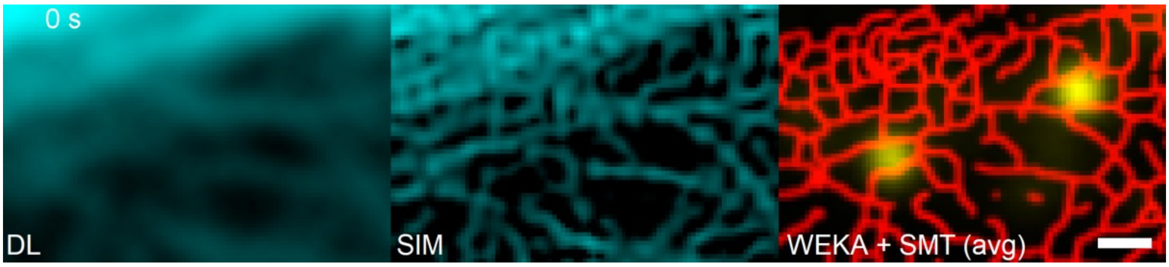

Supplementary Movie 15. Cytoskeletal dynamics during endocytosis of TpoR. Scale bar: 500 nm.

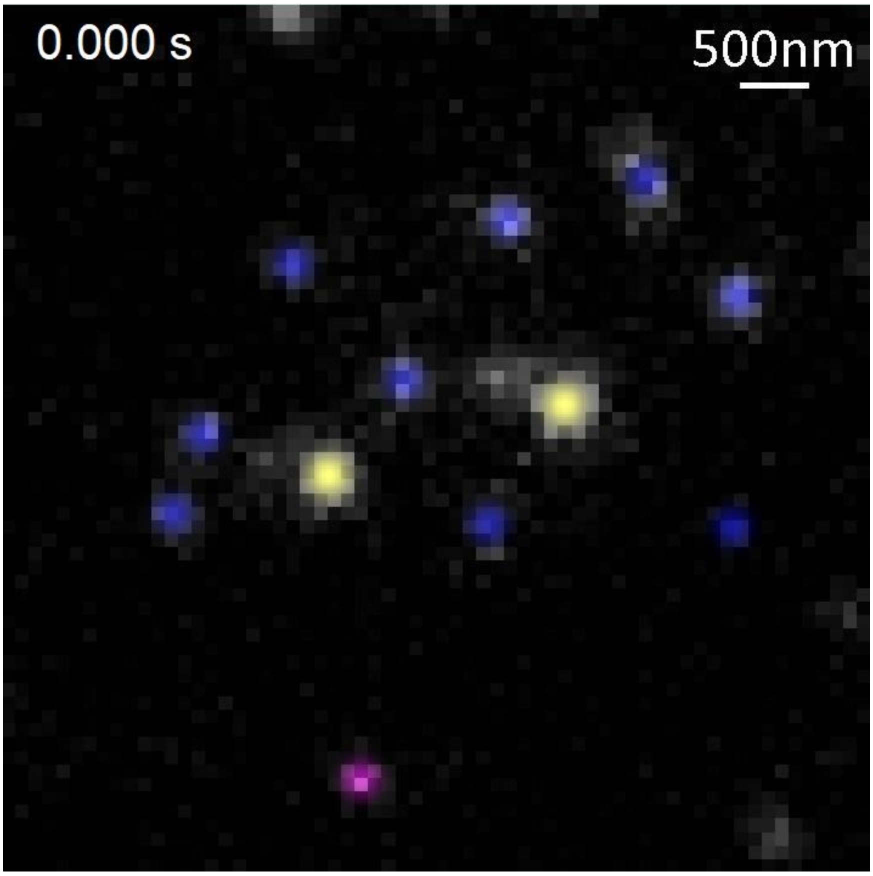

Supplementary Movie 16. Mobility and intensity classification of mobile versus endocytic TpoR signals (for ROI 3 in Fig. 5). After single-molecule localization and cluster/immobility filtering each signal was classified by immobile (red), monomeric signal (<500 photons, blue) and oligomeric signals (>500 photons, green). Thereby, immobile monomeric signals turn to magenta and immobile oligomeric signals turn to yellow. Scale bar: 500 nm.

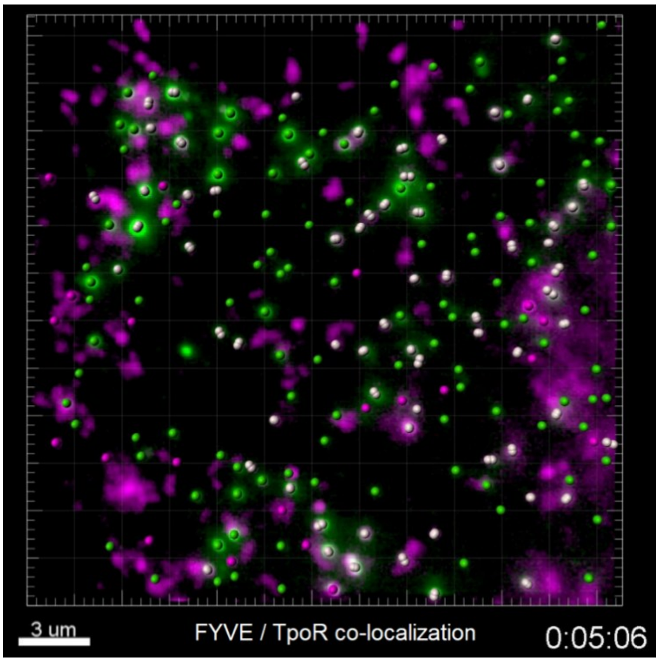

Supplementary Movie 17. Time-lapse co-localization analysis of StayGold-2xFYVE (green) and single-molecule imaging of TpoR dynamics (magenta) time-averaged over 3 seconds (90 frames of raw data) after stimulation with 10 nM thrombopoietin. Co-localized spots are highlighted in white.

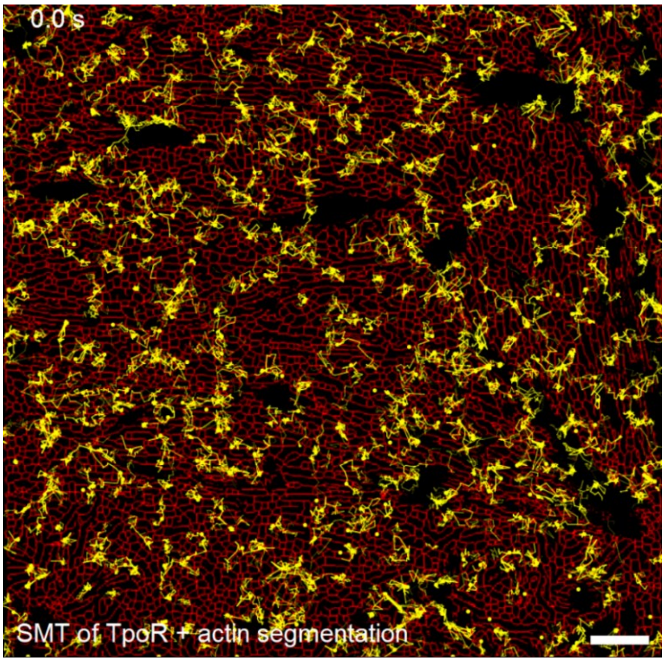

Supplementary Movie 18. Correlative single-molecule imaging (SMI) and tracking (SMT) of TpoR (yellow; raw) with super-resolution of the actin cortex (TIRFM (cyan) vs. TIRF-SIM (cyan) vs. actin segmentation (red)) after stimulation of TpoR with 10 nM of thrombopoietin in presence of JAK2-tdmCherry. Scale bar: 2 µm.

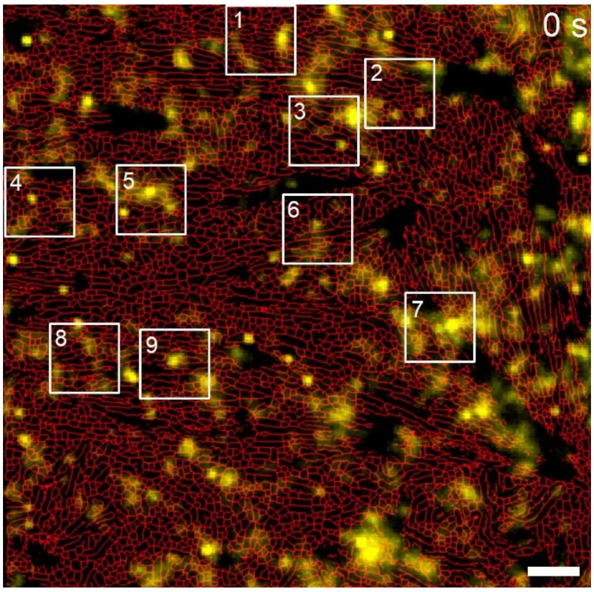

Supplementary Movie 19. Correlation of cytoskeletal dynamics (WEKA segmentation, red) and endocytosed TpoR (time-lapse mean intensity projection of TpoR signals from 90 consecutive frames, yellow) after receptor stimulation with 10 nM of thrombopoietin. Scale bar: 2 µm.

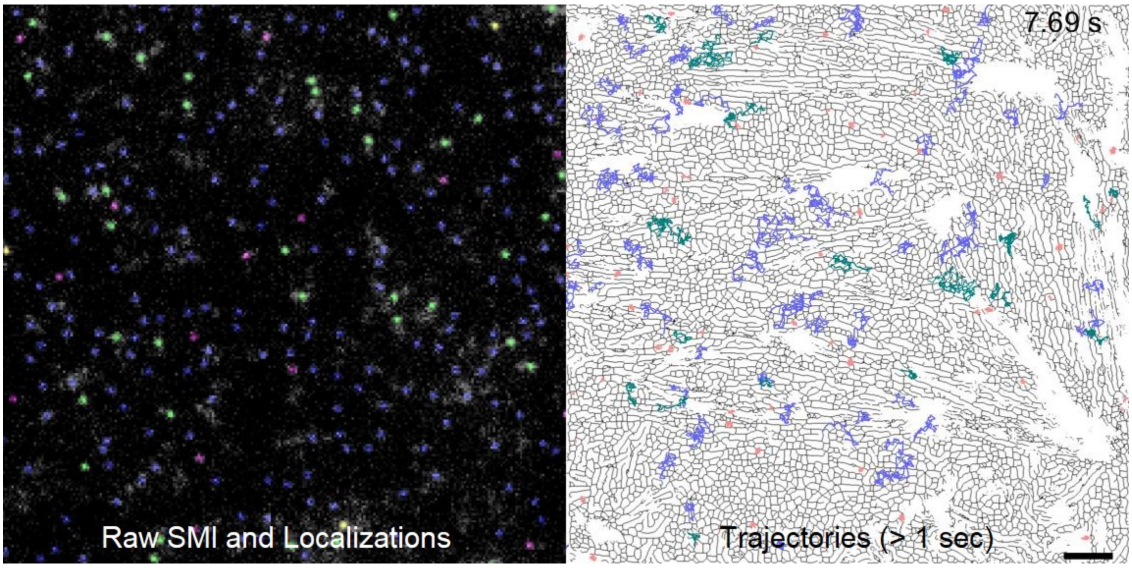

Supplementary Movie 20. Classification of mobility mode (immobile/endocytic vs. mobile) and intensity (monomer vs. oligomeric) on single-molecule level after stimulation by 10 nM thrombopoietin. Left: Each signal was classified by immobile (red), monomeric signal (<165 photons, blue) and oligomeric signals (>165 photons, green) based on single-molecule localization and cluster/immobility filtering. Immobile monomeric signals turn to magenta and immobile oligomeric signals turn to yellow. Right: All trajectories longer than 1 second were overlaid on the underlaying super-resolved actin cortex (red: immobile, blue: monomeric, green: dimeric). Movie shows first 12 seconds of the experiment. Scale bar: 2 µm.

Supplementary Software

Matlab script for FRET efficiency analysis (requires R2022b or higher). Software contains readme file with installation and usage instructions as well as a small demo data set to test software.
