# Supplementary figures and images for: Correlative single-molecule and structured illumination microscopy of fast dynamics at the plasma membrane
Source: Nat Commun. 2024 Jul 10;15:5813. doi: 10.1038/s41467-024-49876-9 (PMC11236984; doi:10.1038/s41467-024-49876-9)

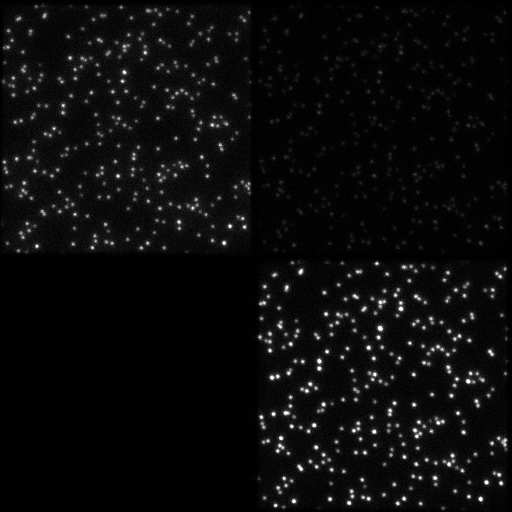

Supplement: Supplementary file 25 — Supplementary Software [file 41467_2024_49876_MOESM25_ESM.zip › FRET_efficiency_analysis/calibration/beads.tif]

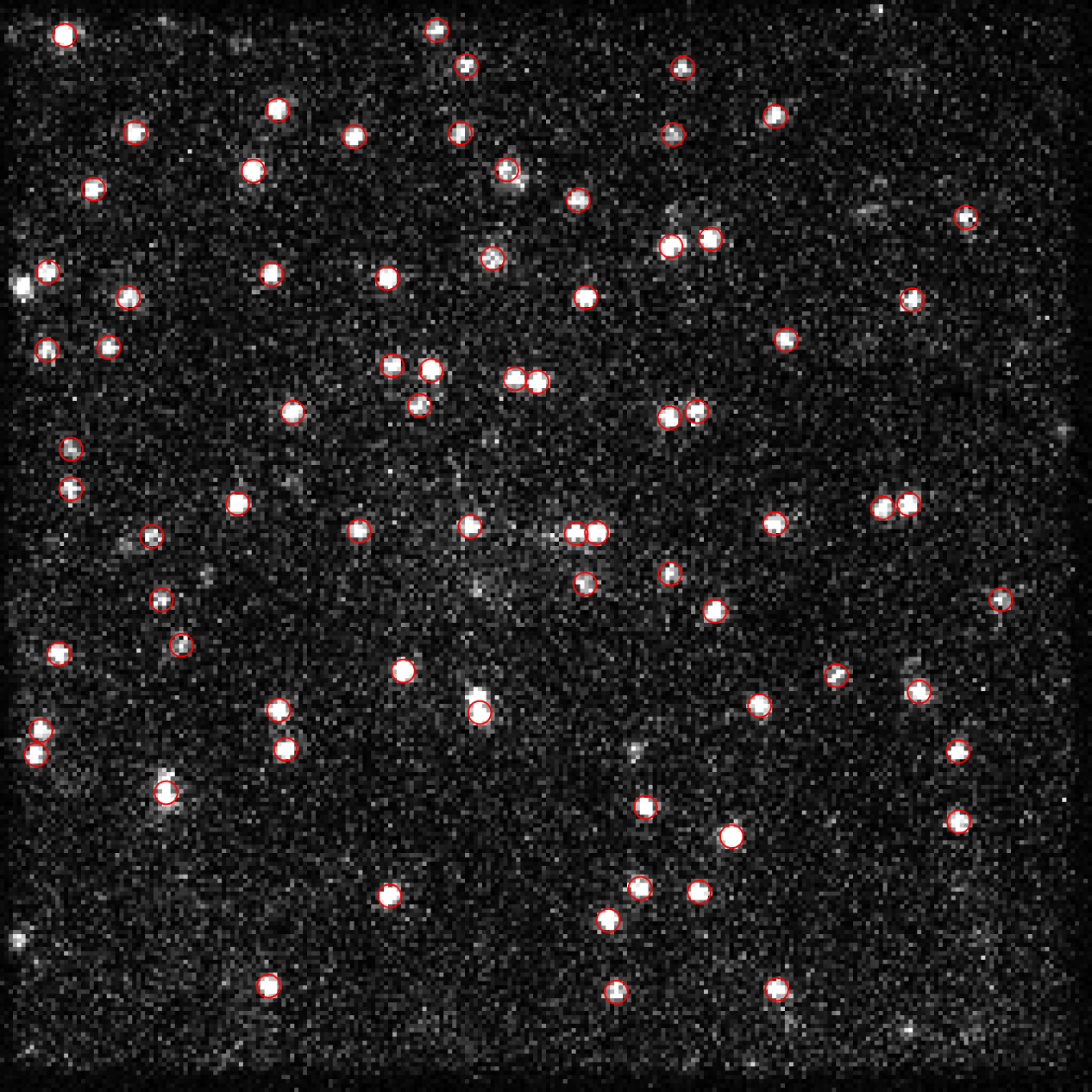

Supplement: Supplementary file 25 — Supplementary Software [file 41467_2024_49876_MOESM25_ESM.zip › FRET_efficiency_analysis/expected output/demo_cell_1/Acceptor_Localization.png]

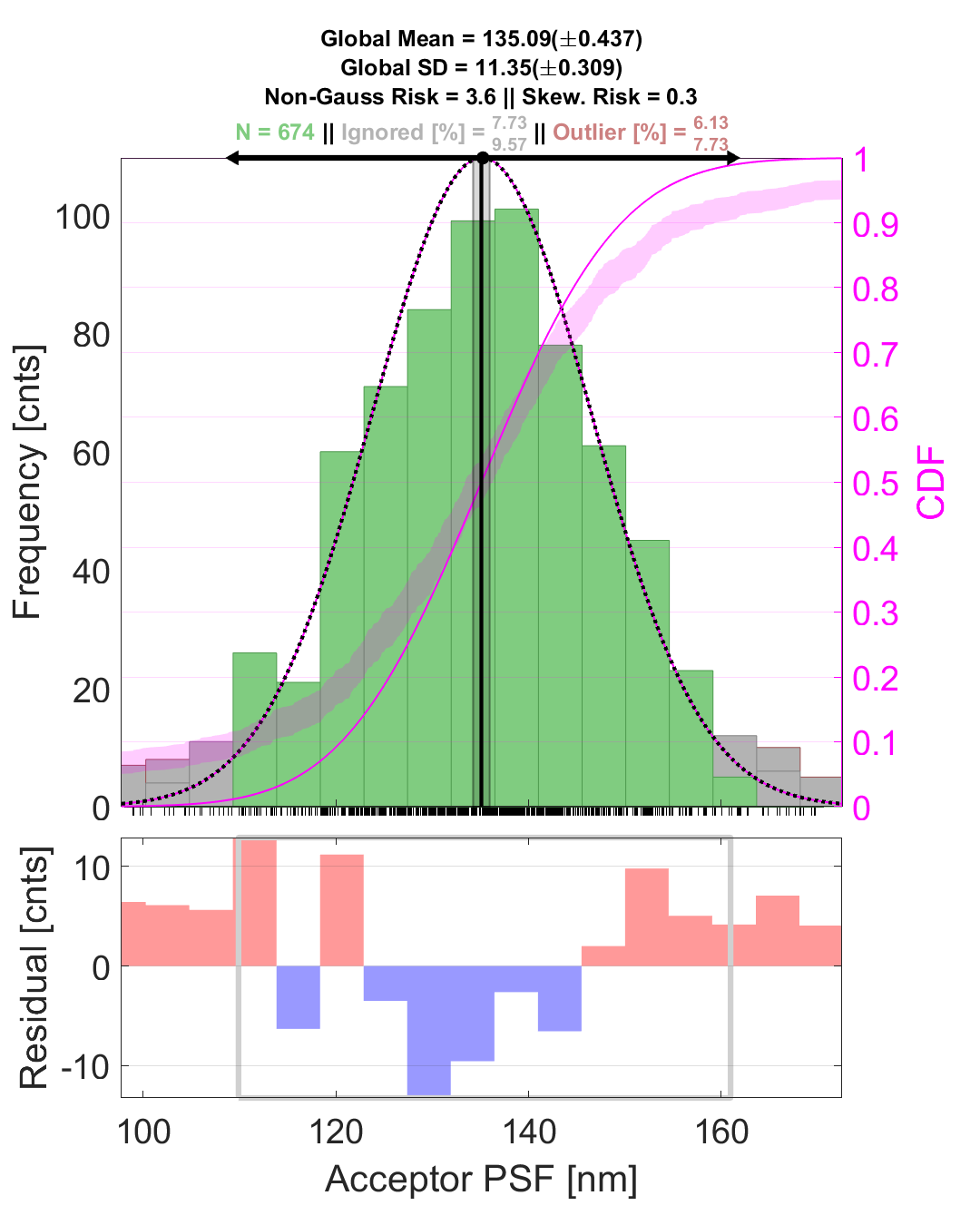

Supplement: Supplementary file 25 — Supplementary Software [file 41467_2024_49876_MOESM25_ESM.zip › FRET_efficiency_analysis/expected output/demo_cell_1/Acceptor_PSF.png]

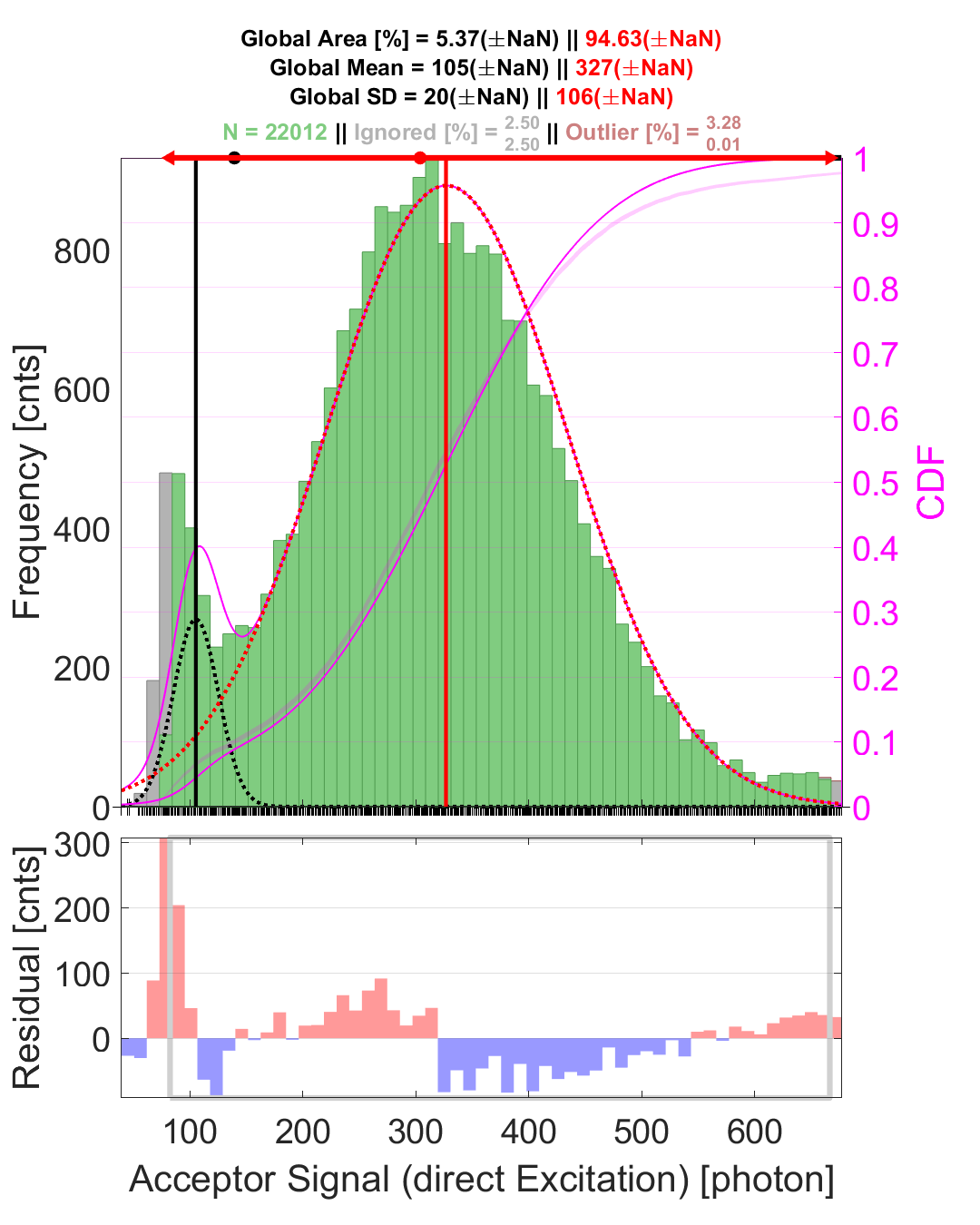

Supplement: Supplementary file 25 — Supplementary Software [file 41467_2024_49876_MOESM25_ESM.zip › FRET_efficiency_analysis/expected output/demo_cell_1/Acceptor_Signal.png]

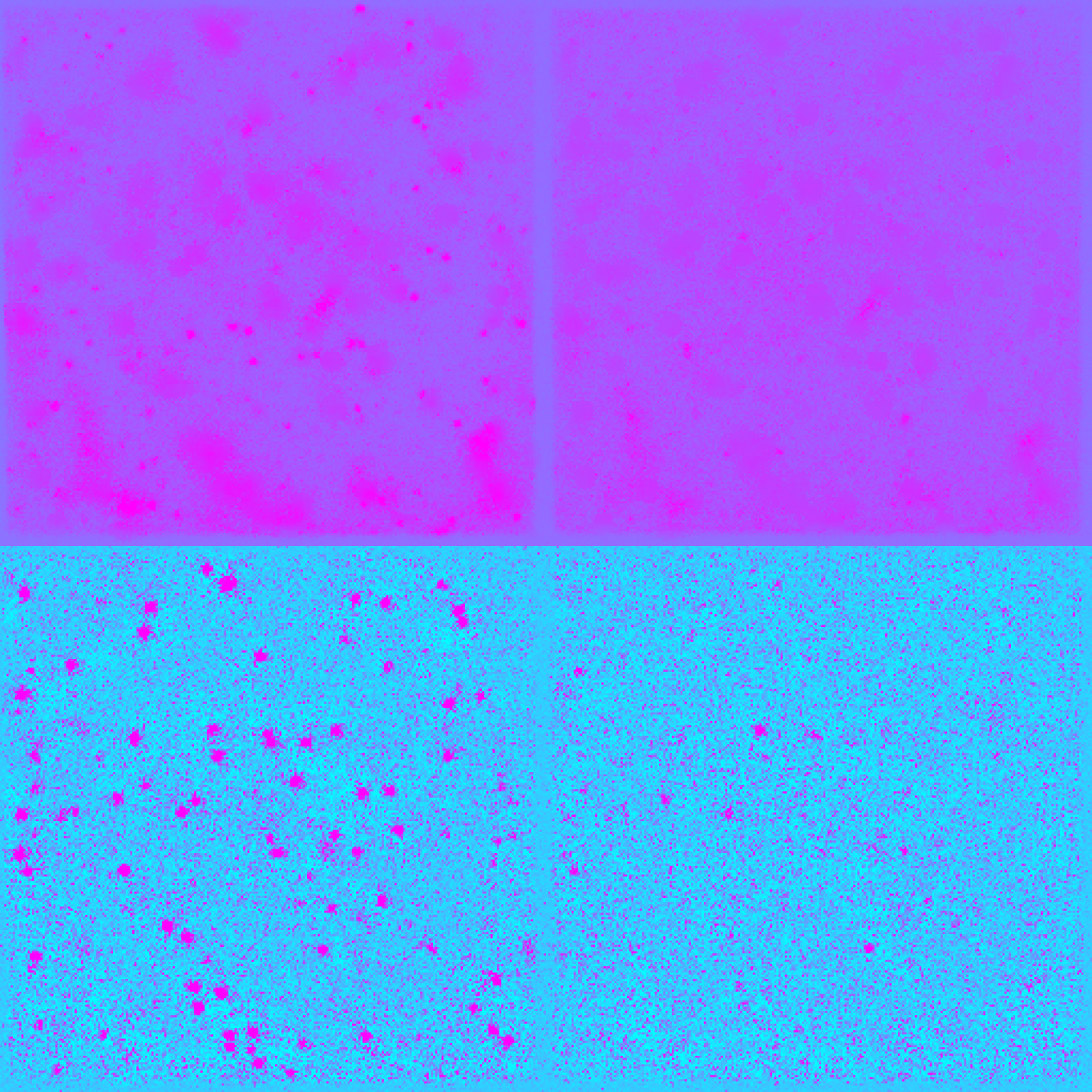

Supplement: Supplementary file 25 — Supplementary Software [file 41467_2024_49876_MOESM25_ESM.zip › FRET_efficiency_analysis/expected output/demo_cell_1/BG corrected frame.png]

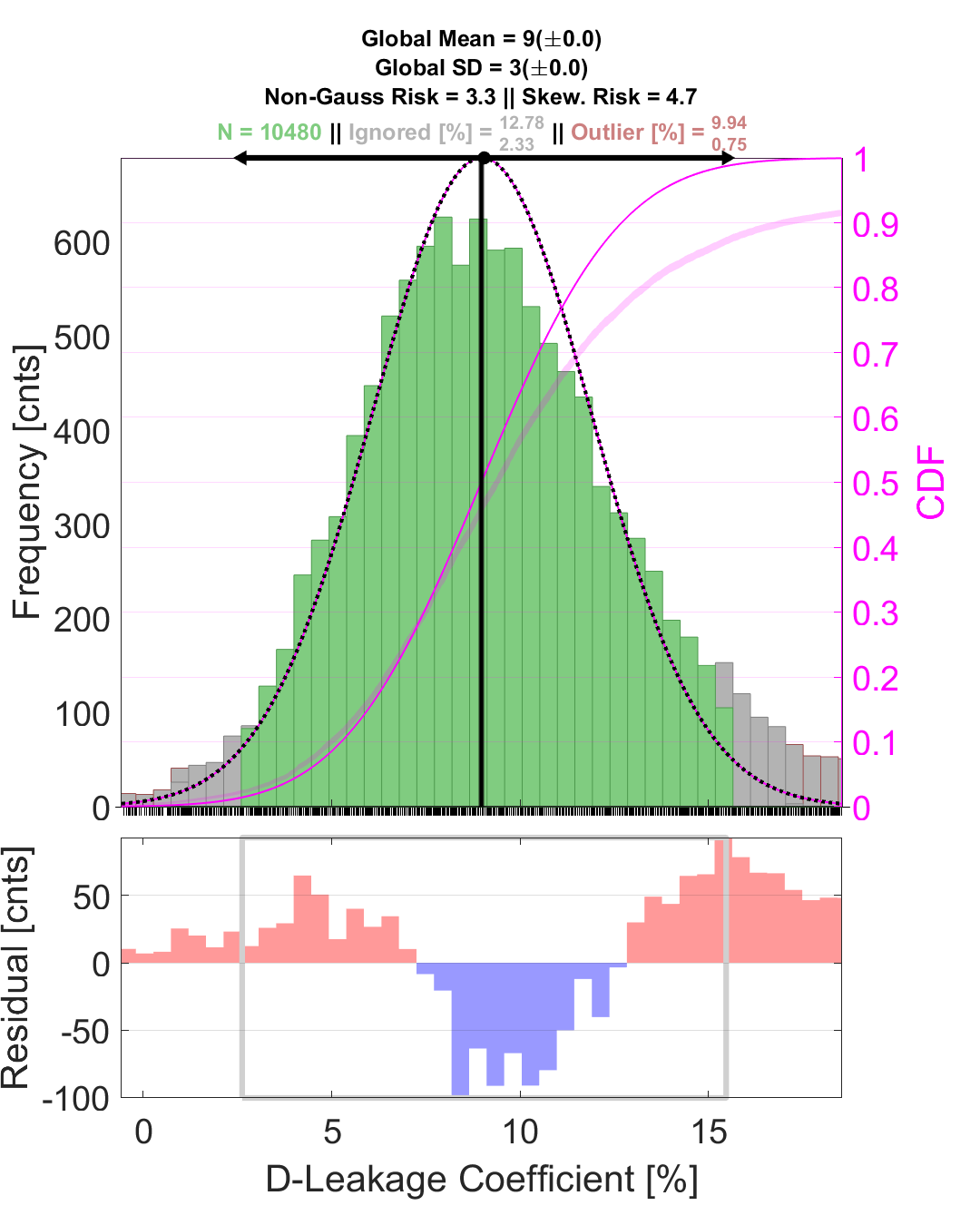

Supplement: Supplementary file 25 — Supplementary Software [file 41467_2024_49876_MOESM25_ESM.zip › FRET_efficiency_analysis/expected output/demo_cell_1/D_Leak_Coeff.png]

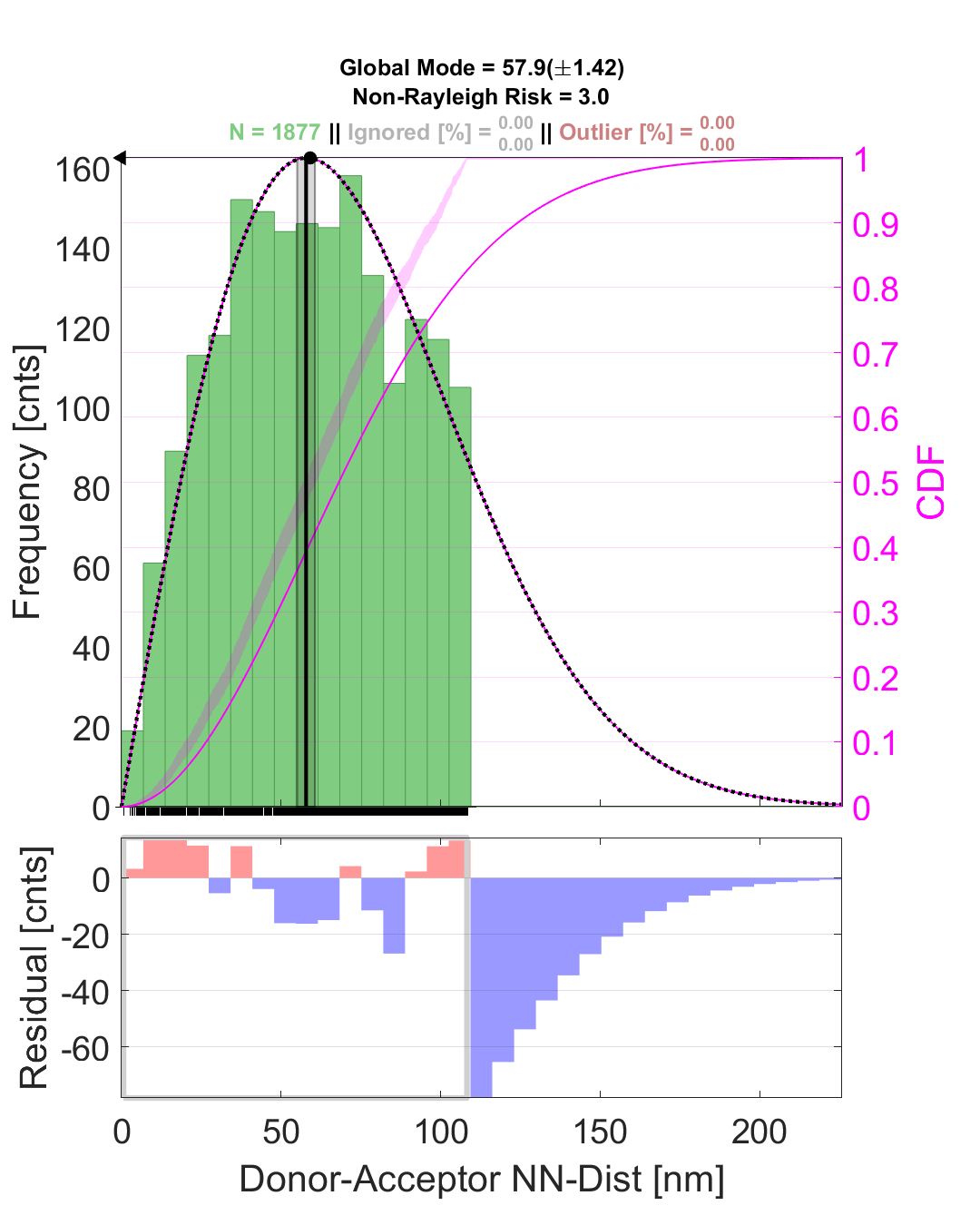

Supplement: Supplementary file 25 — Supplementary Software [file 41467_2024_49876_MOESM25_ESM.zip › FRET_efficiency_analysis/expected output/demo_cell_1/Donor_Acceptor_NN.png]

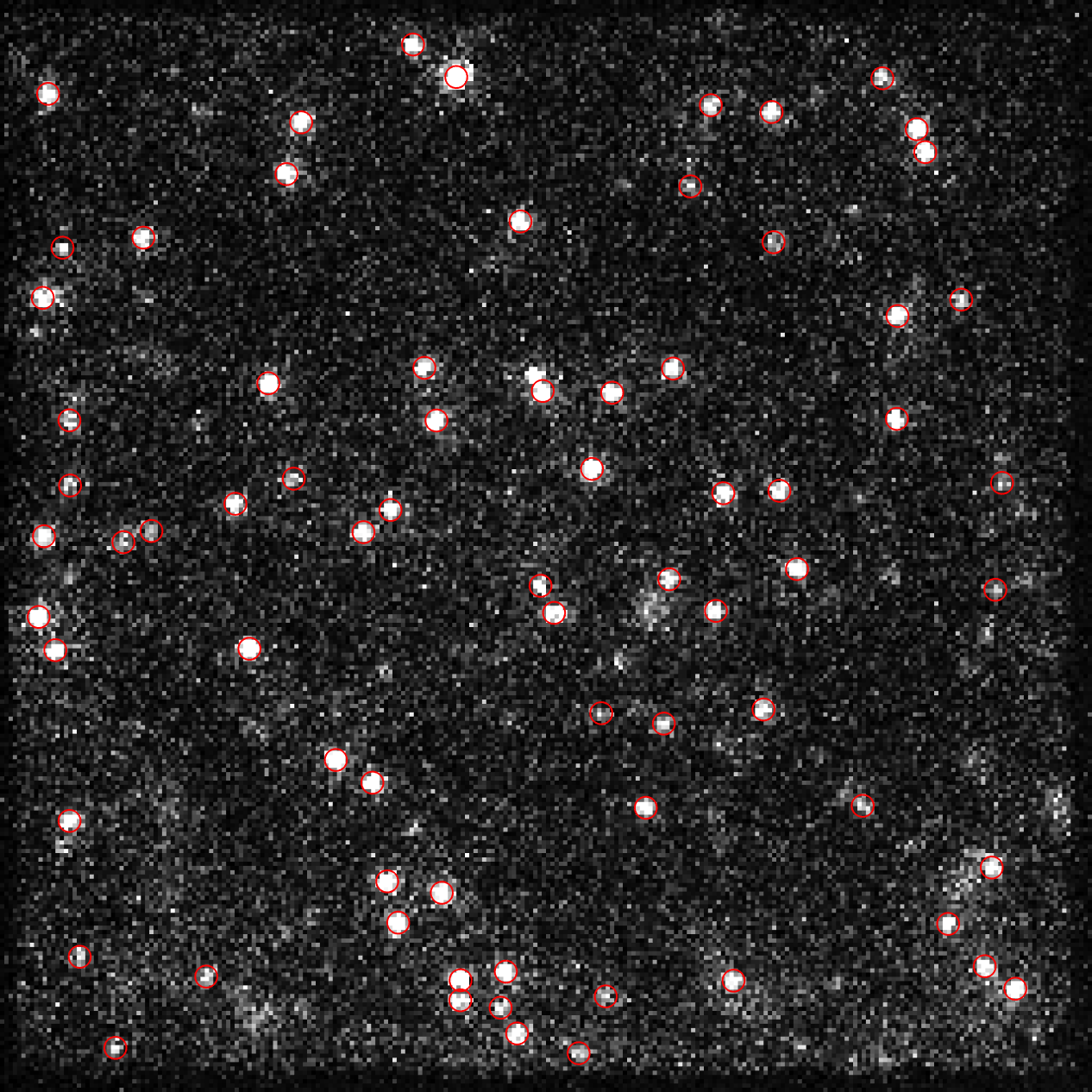

Supplement: Supplementary file 25 — Supplementary Software [file 41467_2024_49876_MOESM25_ESM.zip › FRET_efficiency_analysis/expected output/demo_cell_1/Donor_Localization.png]

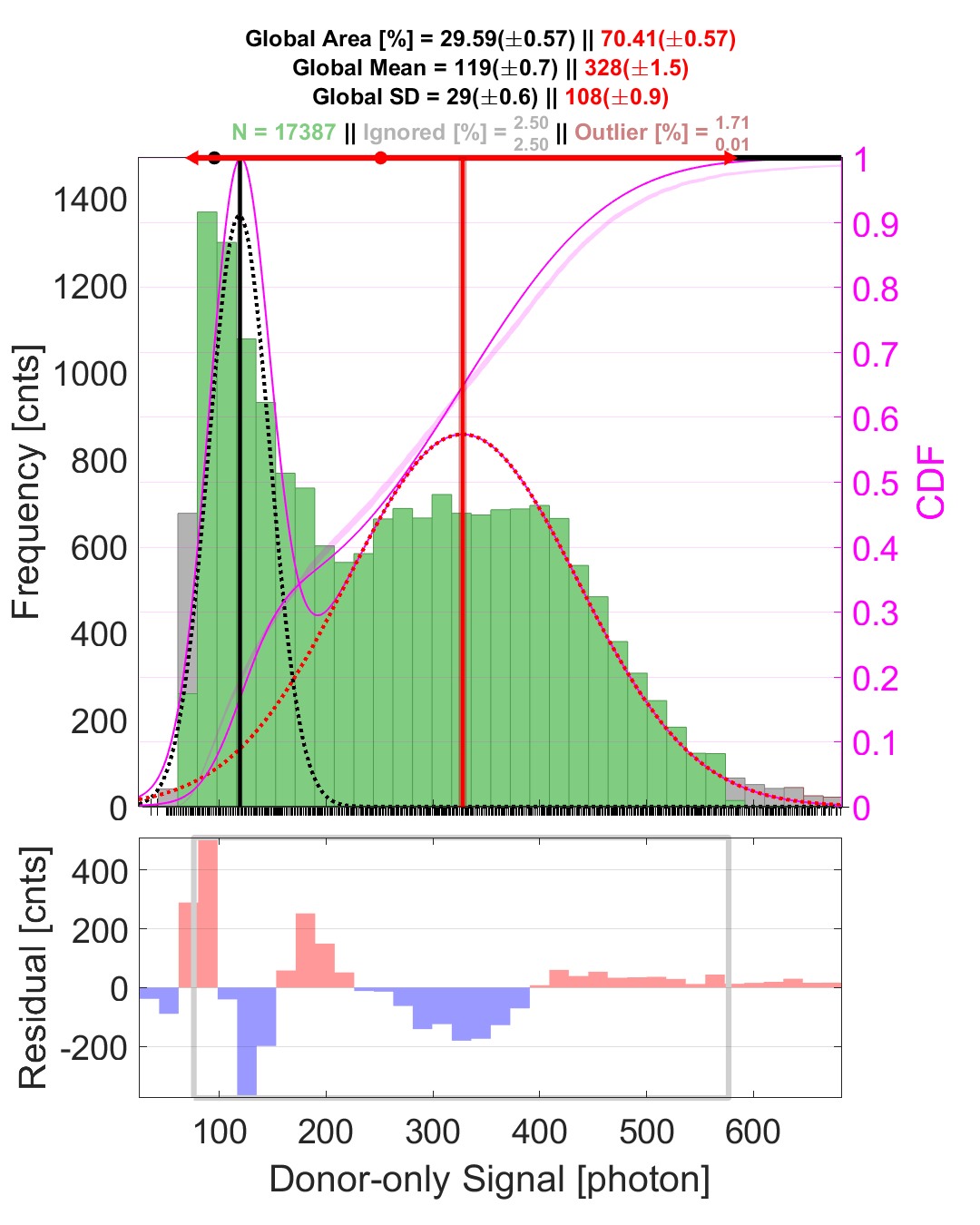

Supplement: Supplementary file 25 — Supplementary Software [file 41467_2024_49876_MOESM25_ESM.zip › FRET_efficiency_analysis/expected output/demo_cell_1/Donor_only_Signal.png]

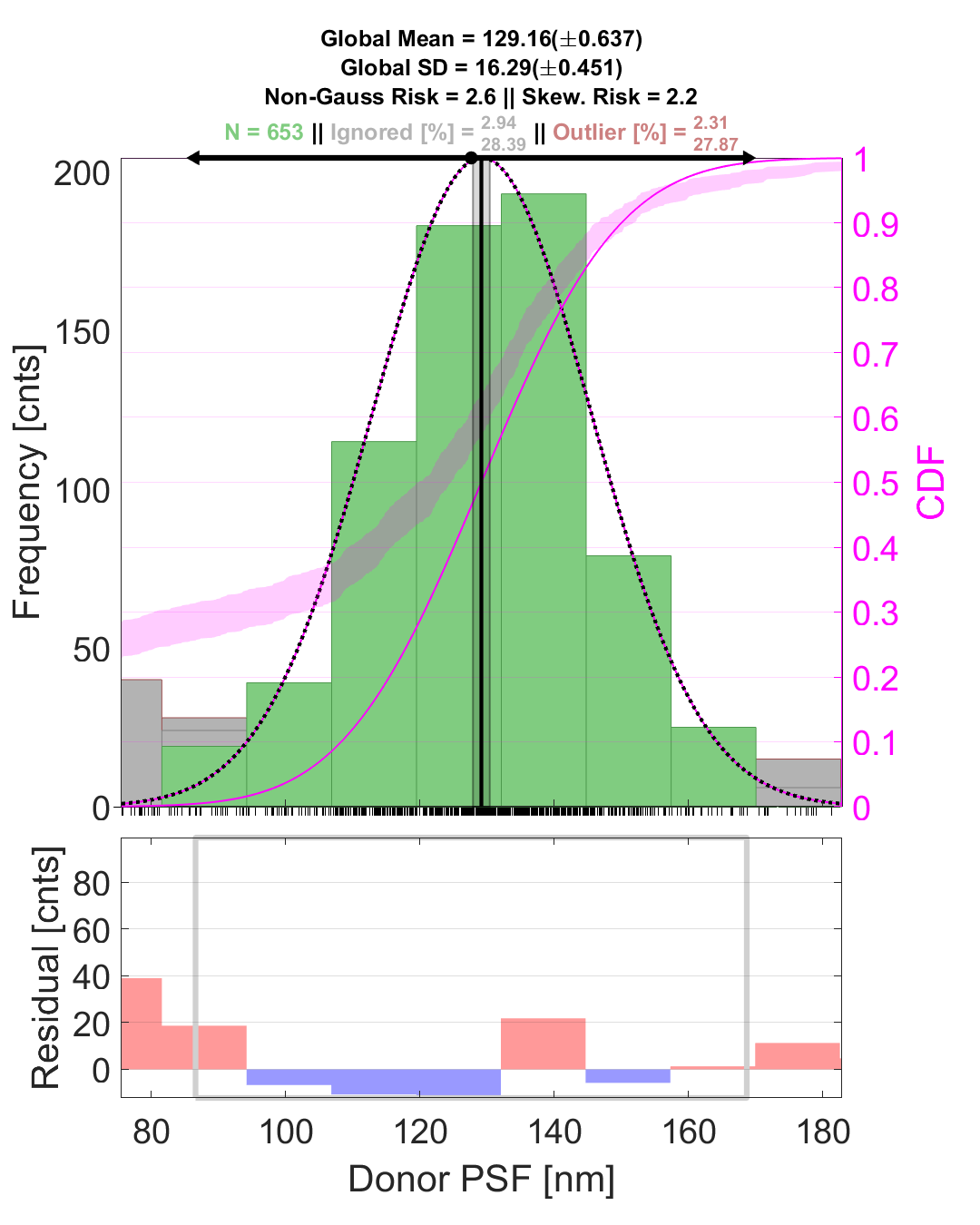

Supplement: Supplementary file 25 — Supplementary Software [file 41467_2024_49876_MOESM25_ESM.zip › FRET_efficiency_analysis/expected output/demo_cell_1/Donor_PSF.png]

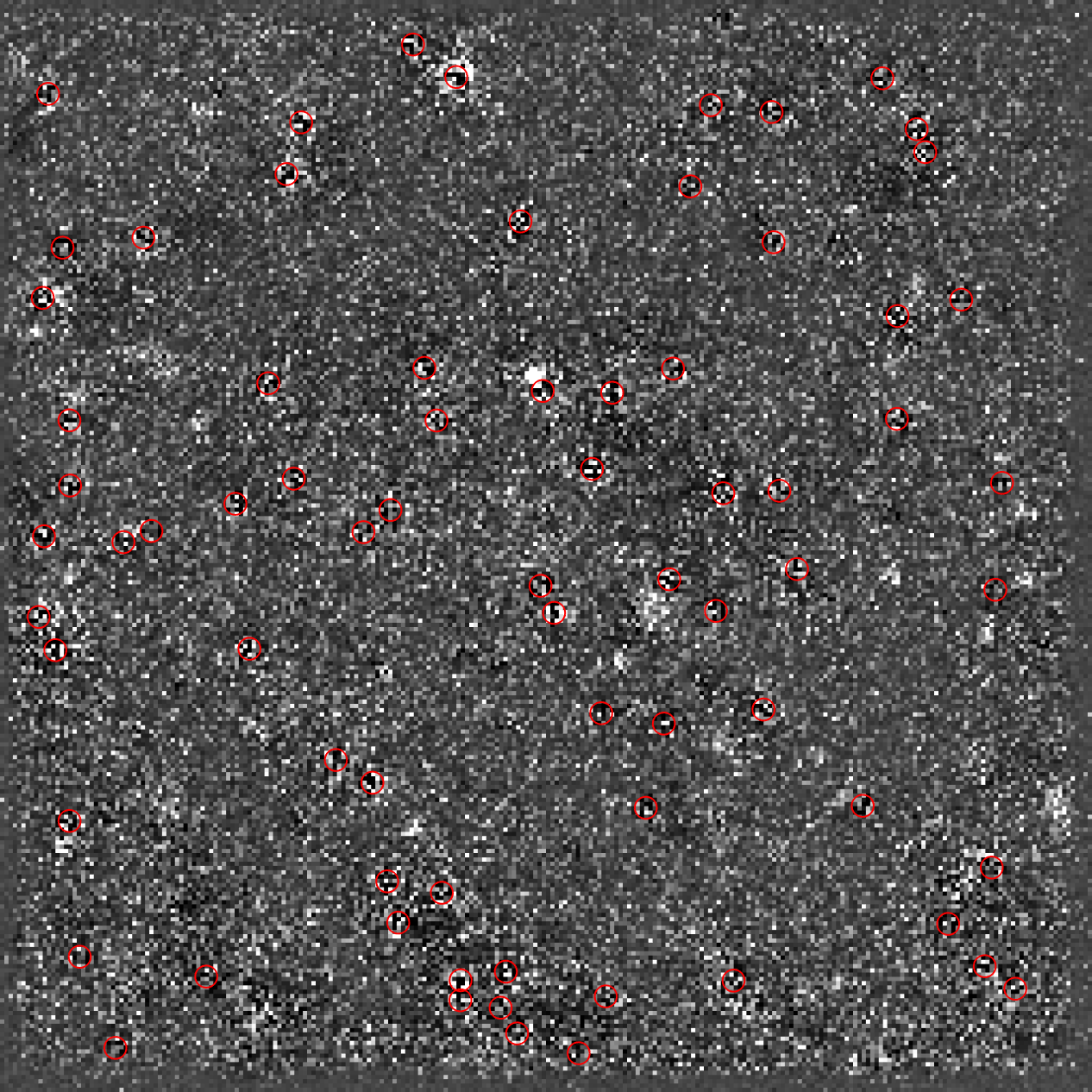

Supplement: Supplementary file 25 — Supplementary Software [file 41467_2024_49876_MOESM25_ESM.zip › FRET_efficiency_analysis/expected output/demo_cell_1/Donor_Res.png]

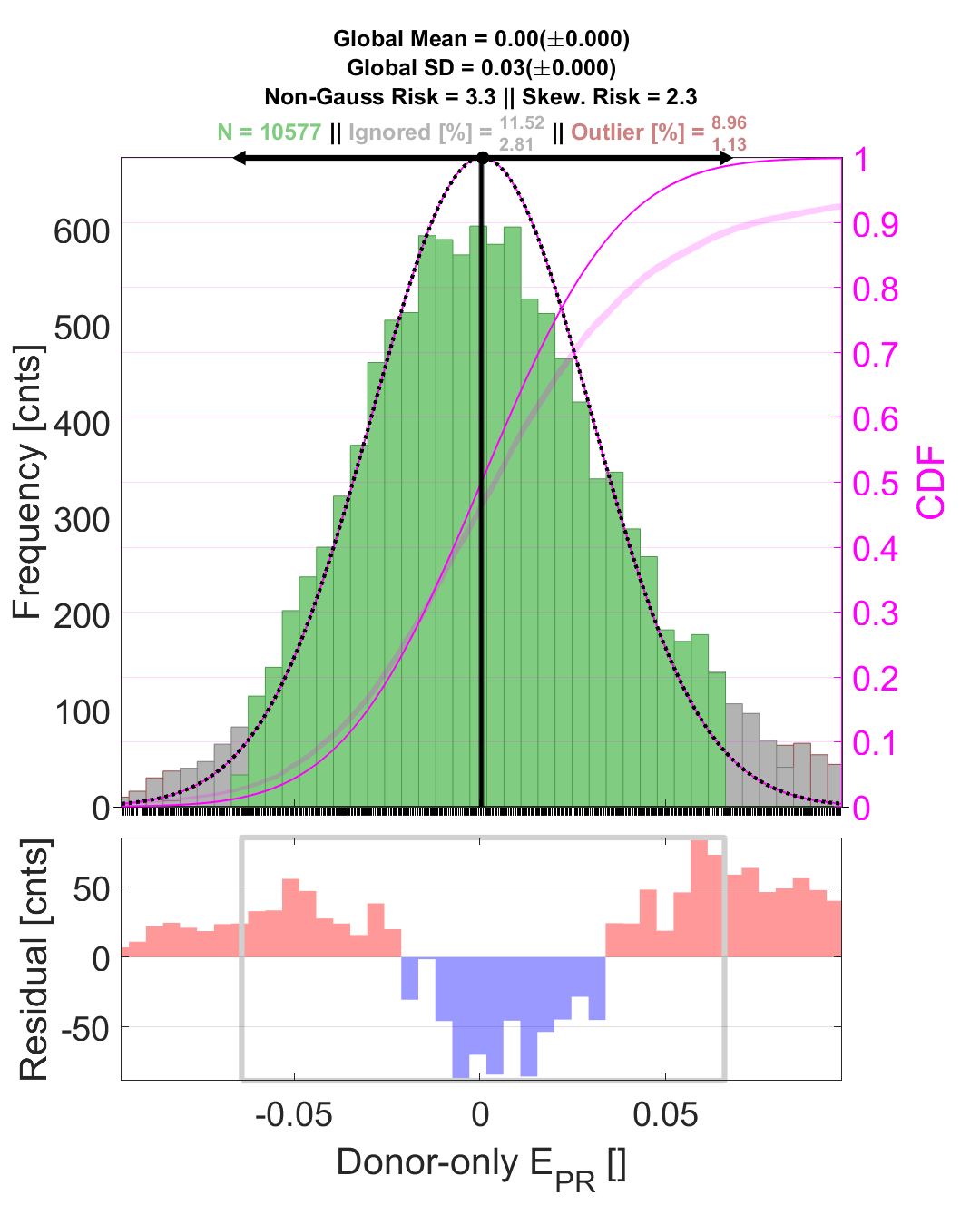

Supplement: Supplementary file 25 — Supplementary Software [file 41467_2024_49876_MOESM25_ESM.zip › FRET_efficiency_analysis/expected output/demo_cell_1/Donor-only EPR.png]

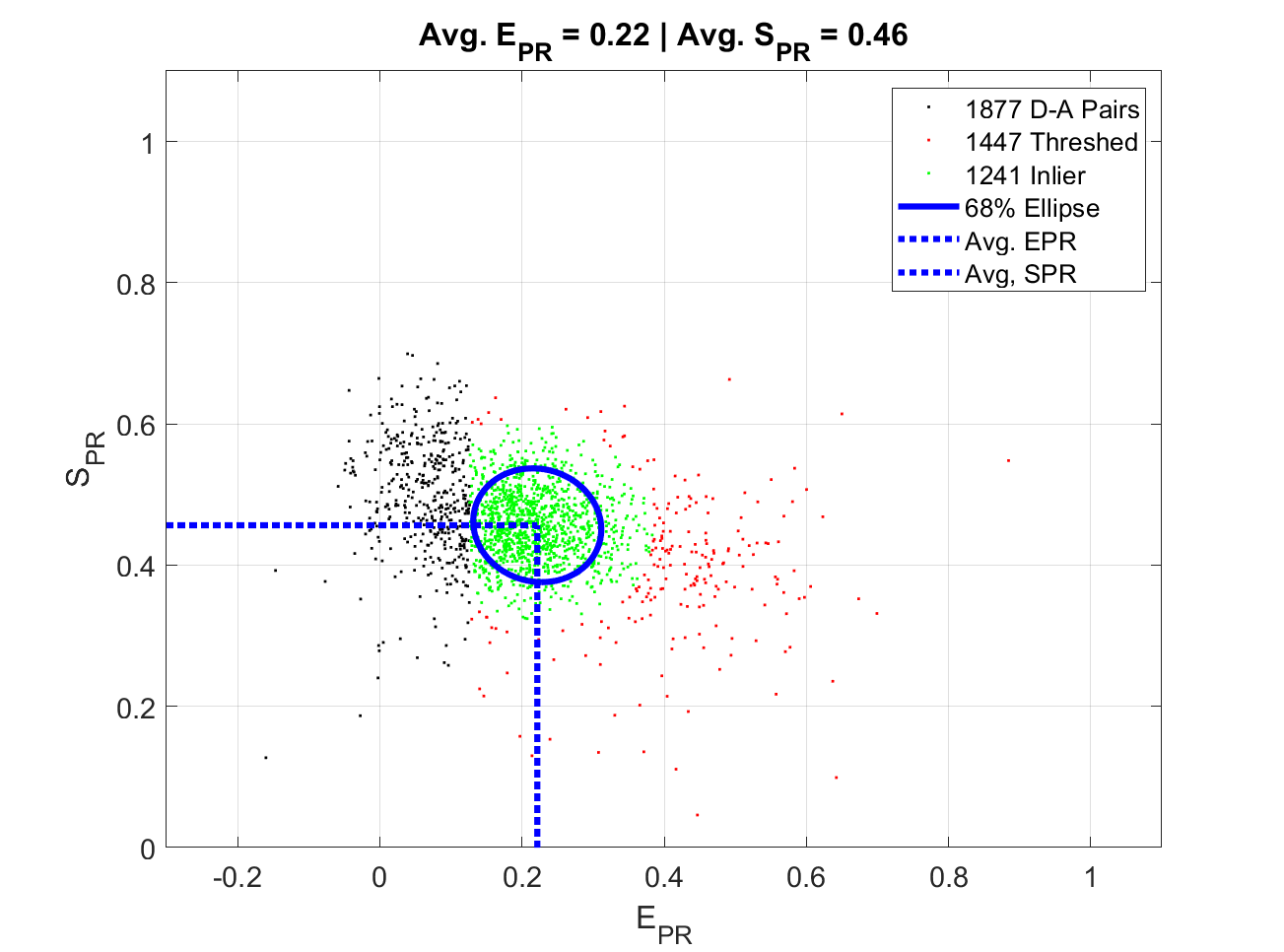

Supplement: Supplementary file 25 — Supplementary Software [file 41467_2024_49876_MOESM25_ESM.zip › FRET_efficiency_analysis/expected output/demo_cell_1/EPR_vs_SPR.png]

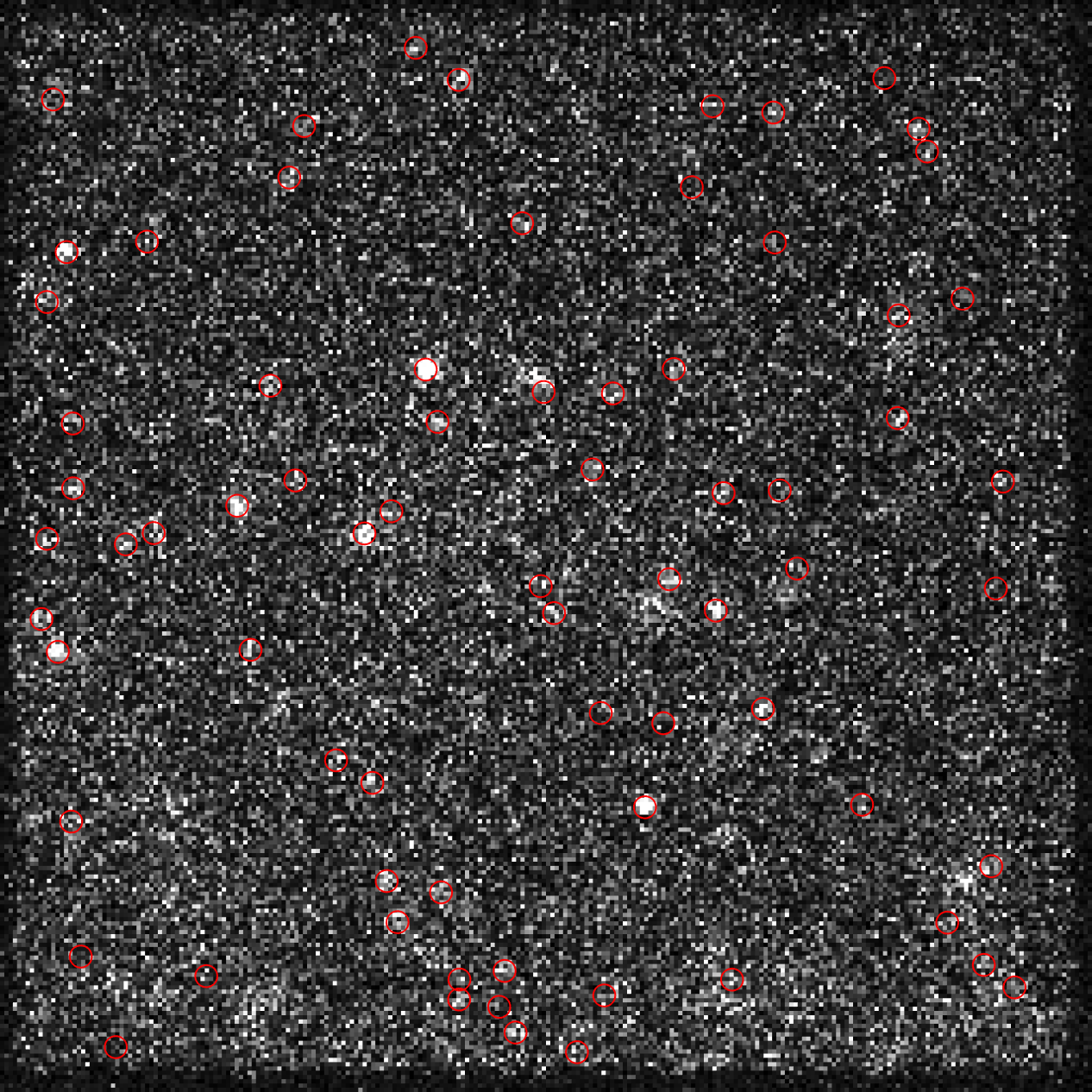

Supplement: Supplementary file 25 — Supplementary Software [file 41467_2024_49876_MOESM25_ESM.zip › FRET_efficiency_analysis/expected output/demo_cell_1/FRET_Localization.png]

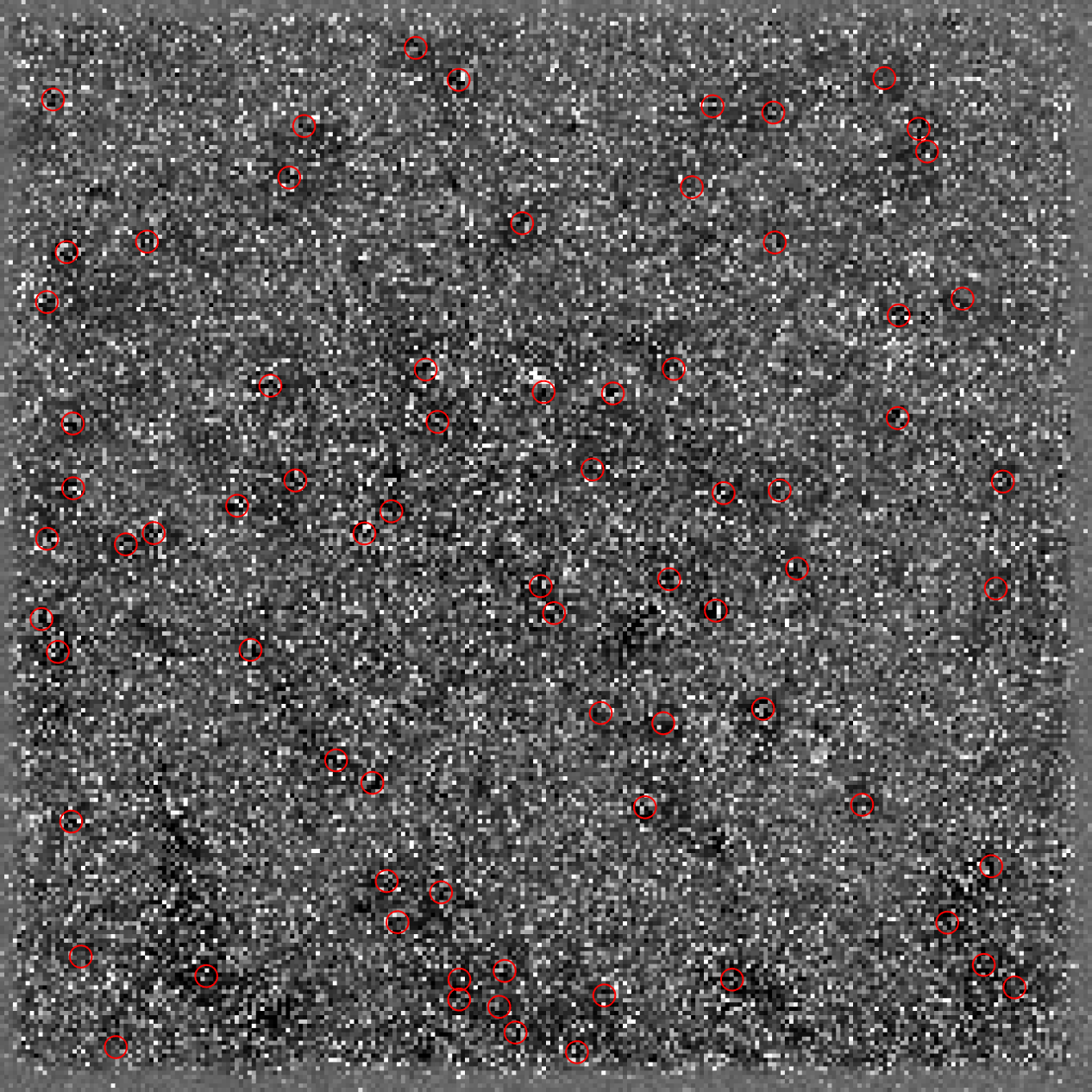

Supplement: Supplementary file 25 — Supplementary Software [file 41467_2024_49876_MOESM25_ESM.zip › FRET_efficiency_analysis/expected output/demo_cell_1/FRET_Res.png]

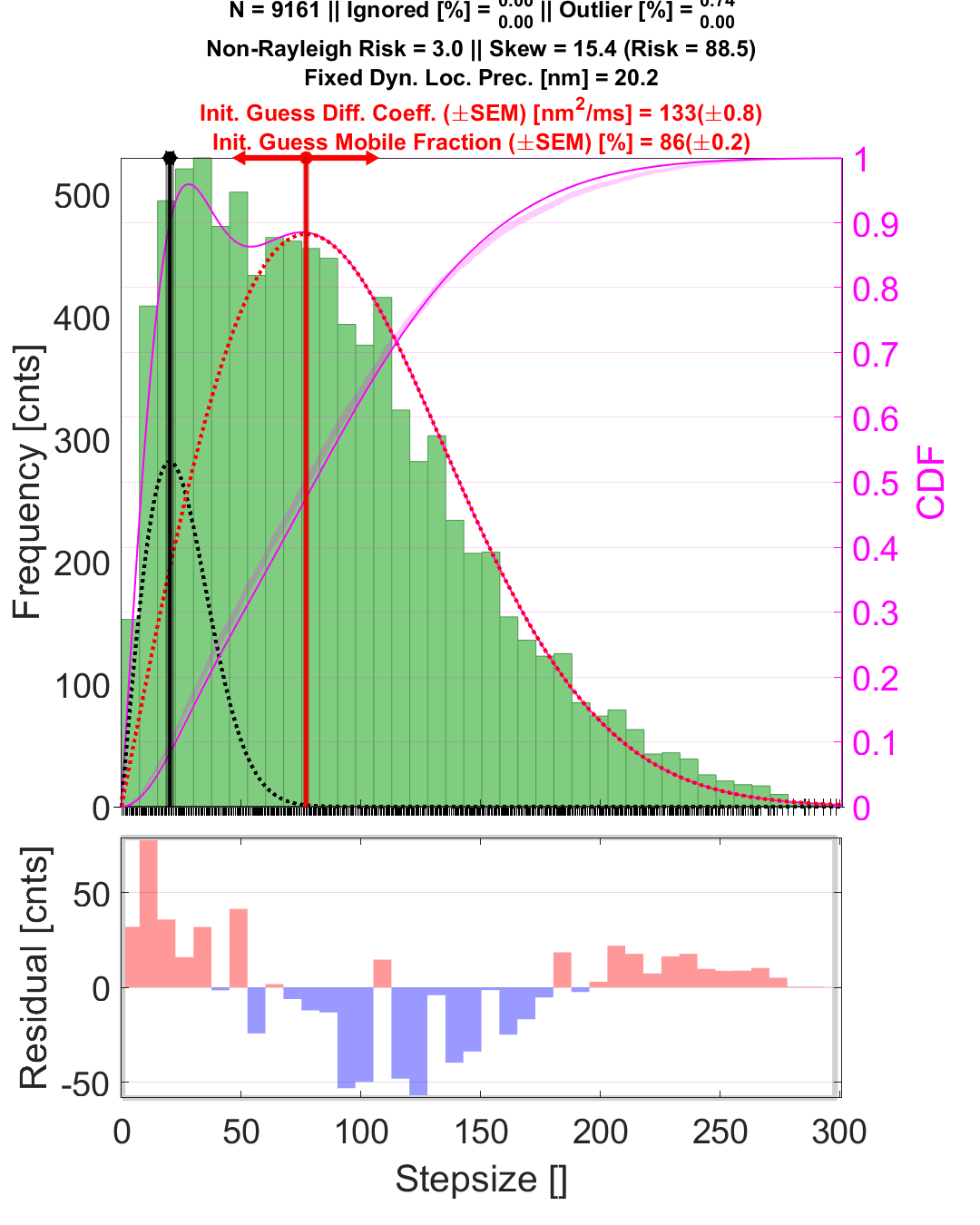

Supplement: Supplementary file 25 — Supplementary Software [file 41467_2024_49876_MOESM25_ESM.zip › FRET_efficiency_analysis/expected output/demo_cell_1/Stepsize.png]

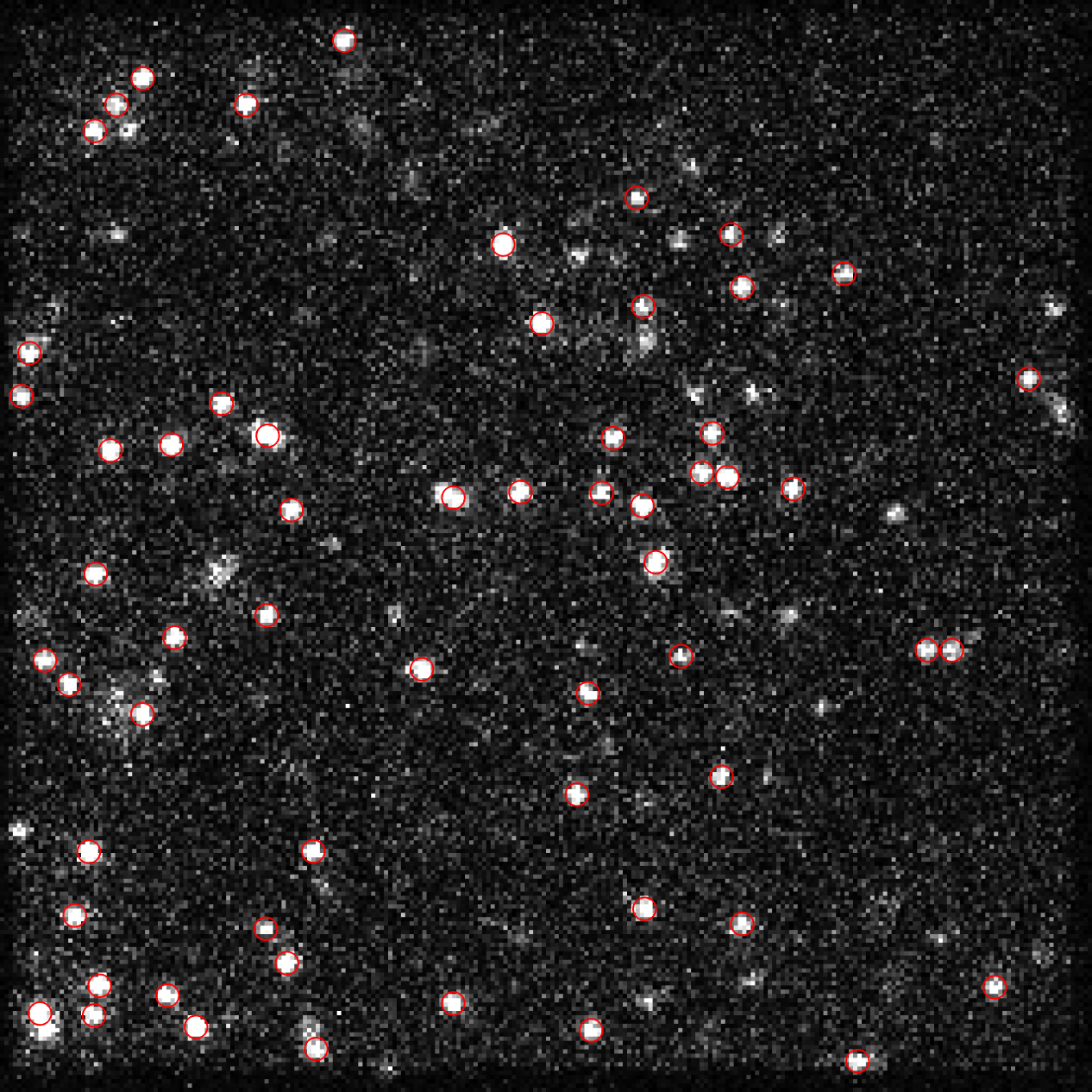

Supplement: Supplementary file 25 — Supplementary Software [file 41467_2024_49876_MOESM25_ESM.zip › FRET_efficiency_analysis/expected output/demo_cell_2/Acceptor_Localization.png]

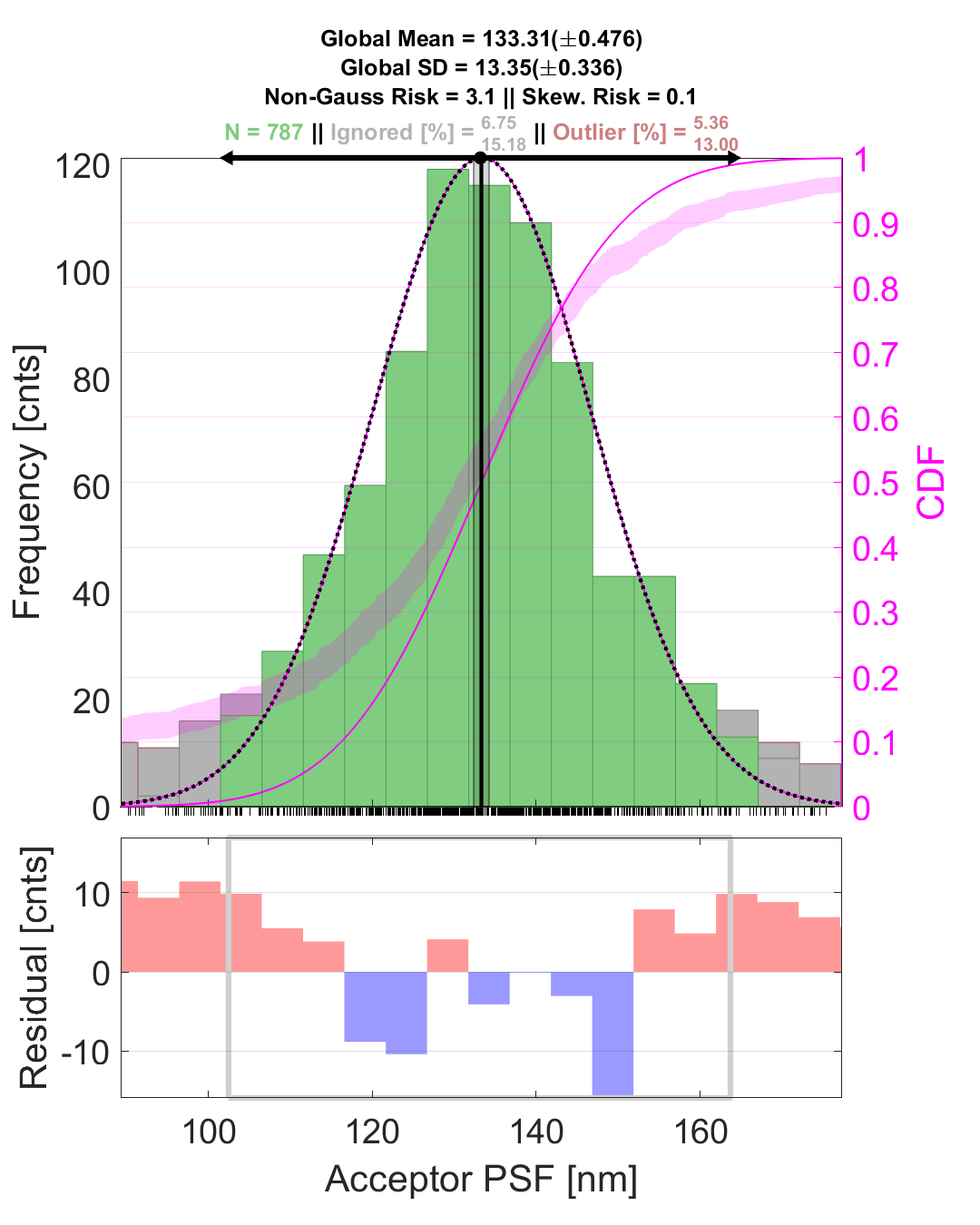

Supplement: Supplementary file 25 — Supplementary Software [file 41467_2024_49876_MOESM25_ESM.zip › FRET_efficiency_analysis/expected output/demo_cell_2/Acceptor_PSF.png]

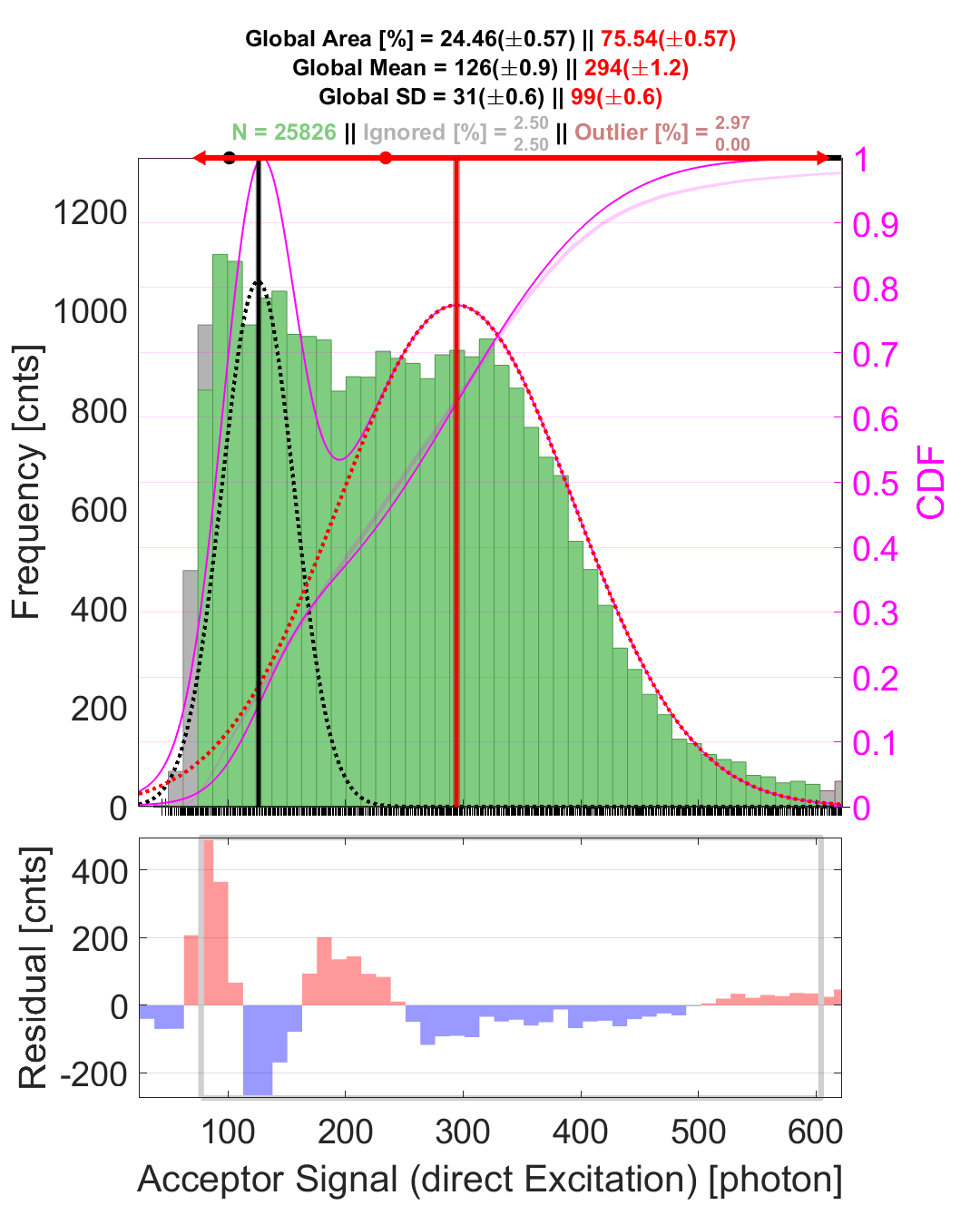

Supplement: Supplementary file 25 — Supplementary Software [file 41467_2024_49876_MOESM25_ESM.zip › FRET_efficiency_analysis/expected output/demo_cell_2/Acceptor_Signal.png]

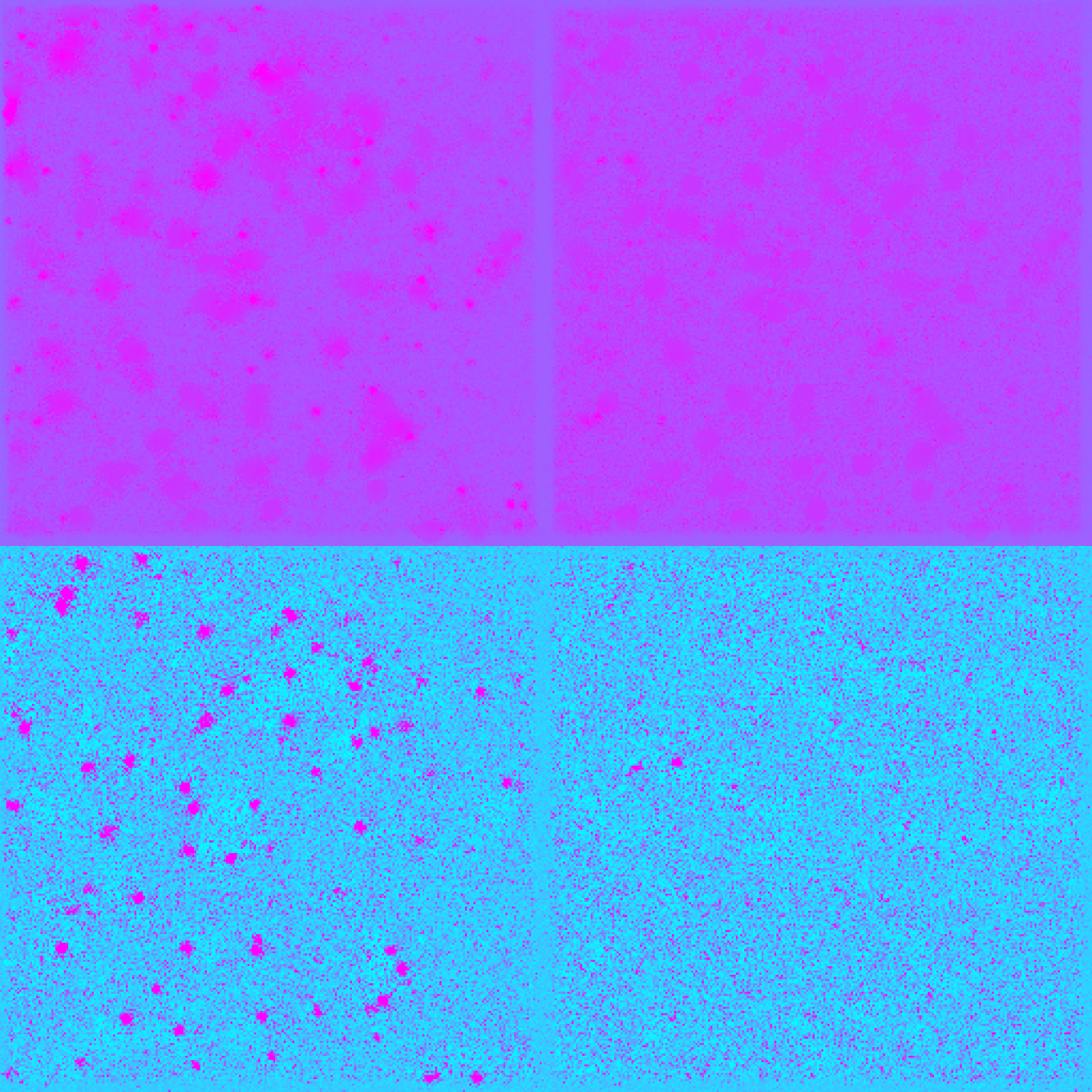

Supplement: Supplementary file 25 — Supplementary Software [file 41467_2024_49876_MOESM25_ESM.zip › FRET_efficiency_analysis/expected output/demo_cell_2/BG corrected frame.png]

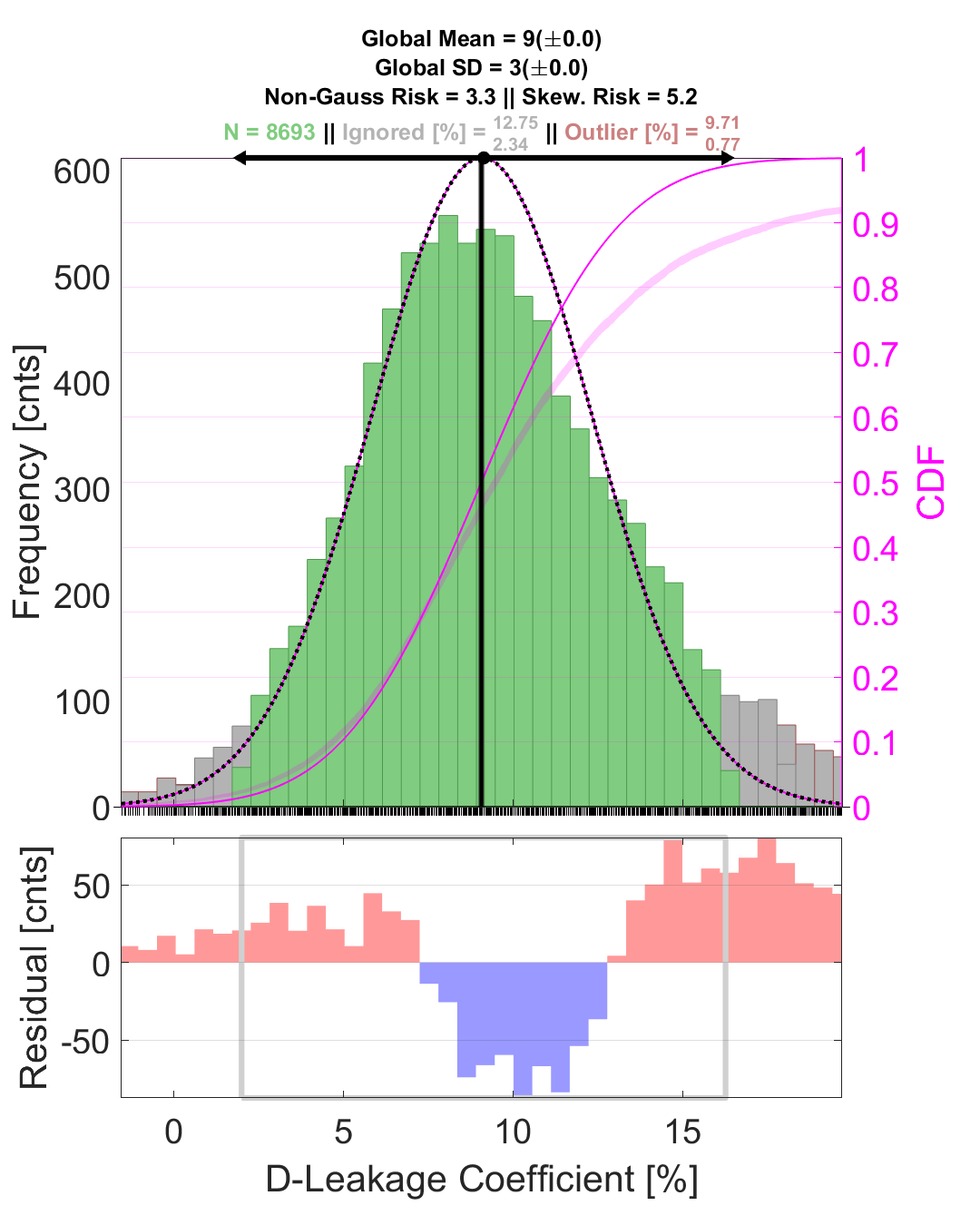

Supplement: Supplementary file 25 — Supplementary Software [file 41467_2024_49876_MOESM25_ESM.zip › FRET_efficiency_analysis/expected output/demo_cell_2/D_Leak_Coeff.png]

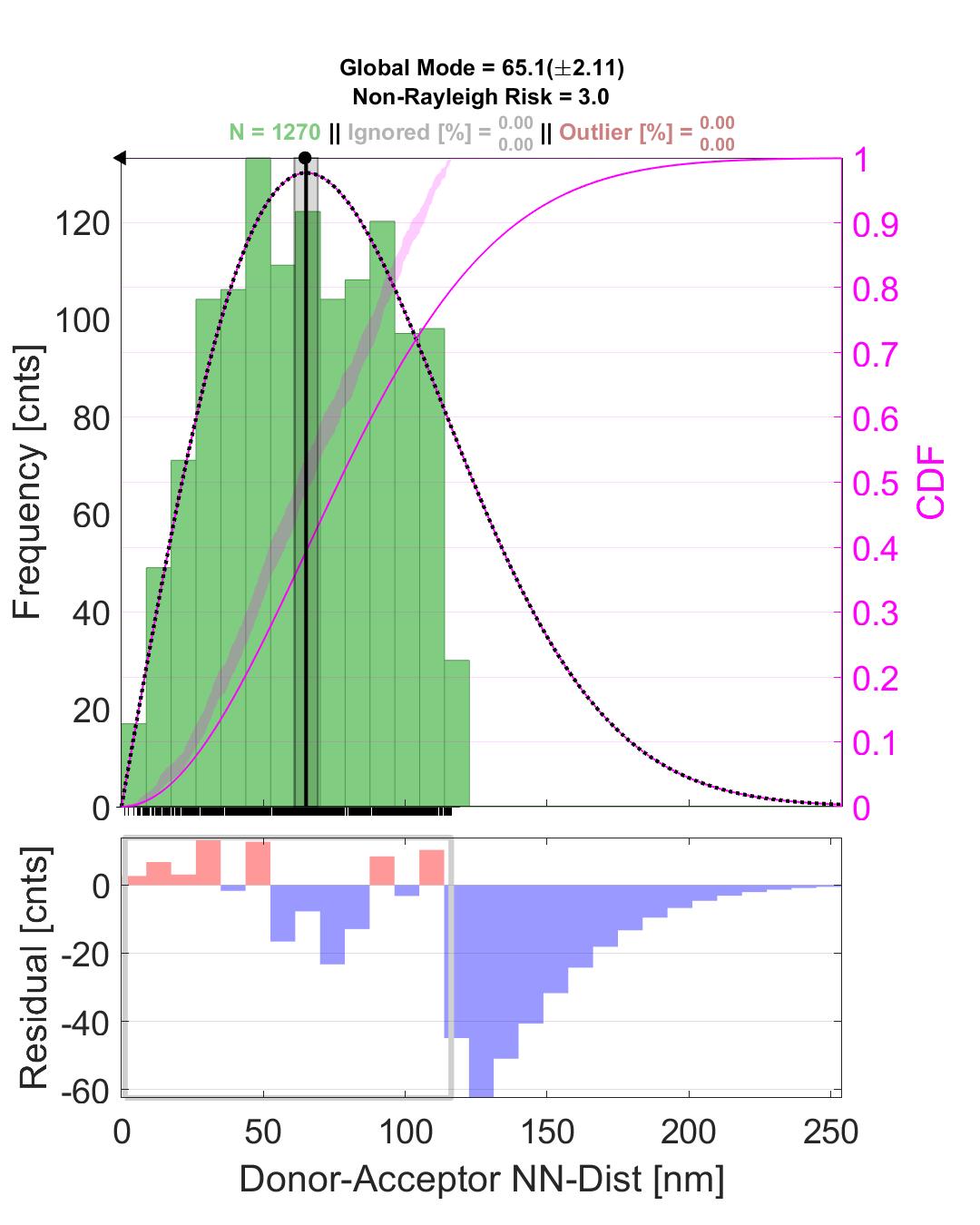

Supplement: Supplementary file 25 — Supplementary Software [file 41467_2024_49876_MOESM25_ESM.zip › FRET_efficiency_analysis/expected output/demo_cell_2/Donor_Acceptor_NN.png]

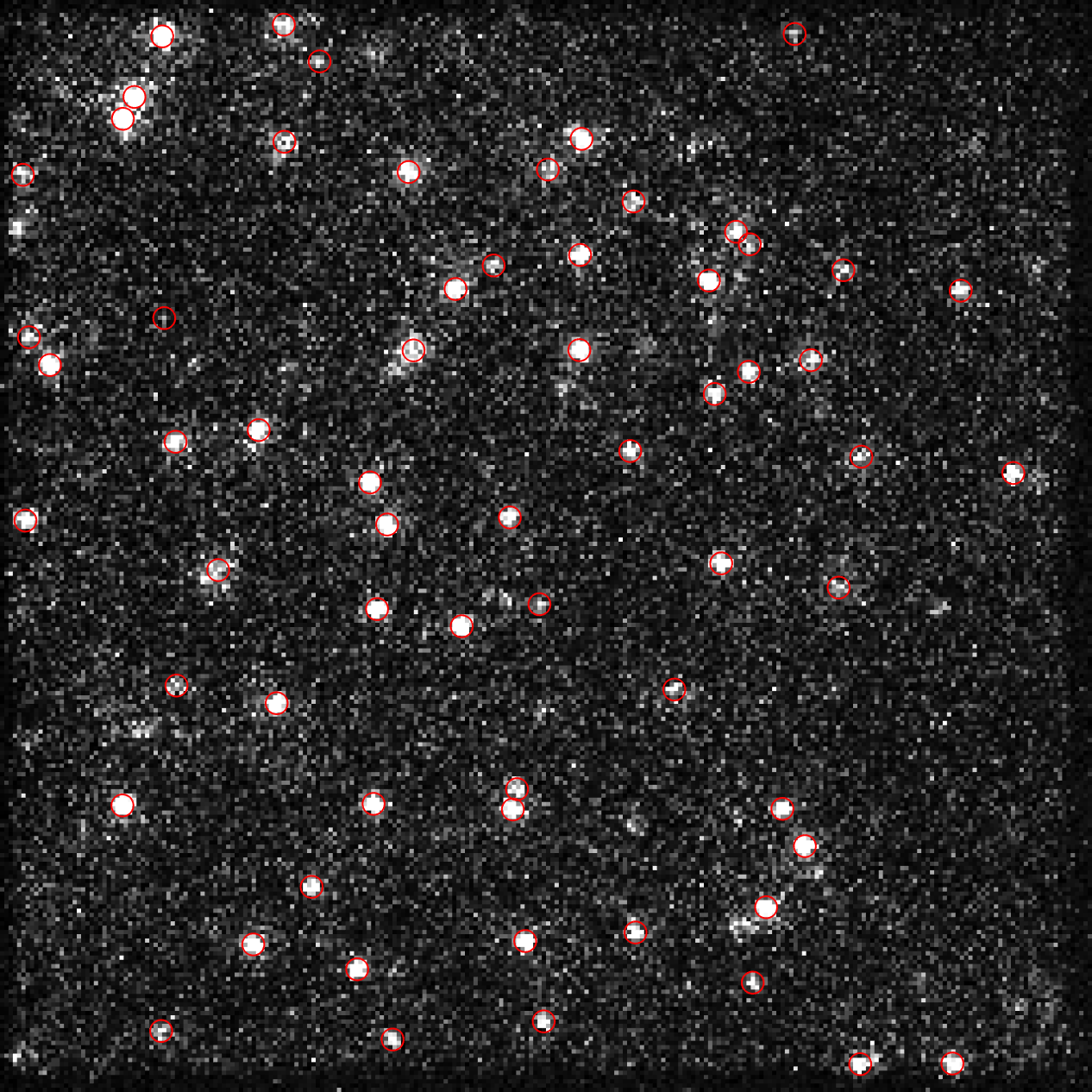

Supplement: Supplementary file 25 — Supplementary Software [file 41467_2024_49876_MOESM25_ESM.zip › FRET_efficiency_analysis/expected output/demo_cell_2/Donor_Localization.png]

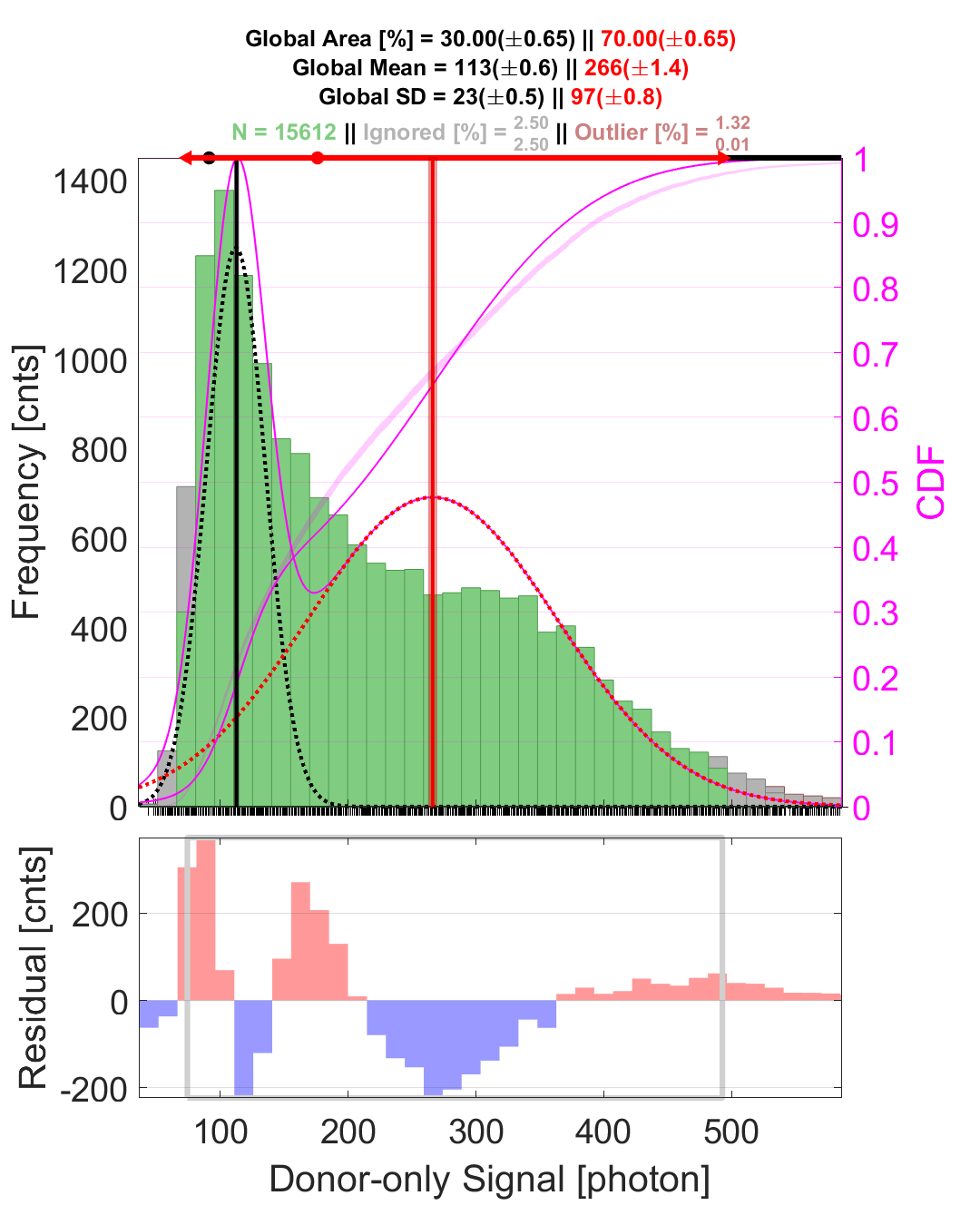

Supplement: Supplementary file 25 — Supplementary Software [file 41467_2024_49876_MOESM25_ESM.zip › FRET_efficiency_analysis/expected output/demo_cell_2/Donor_only_Signal.png]

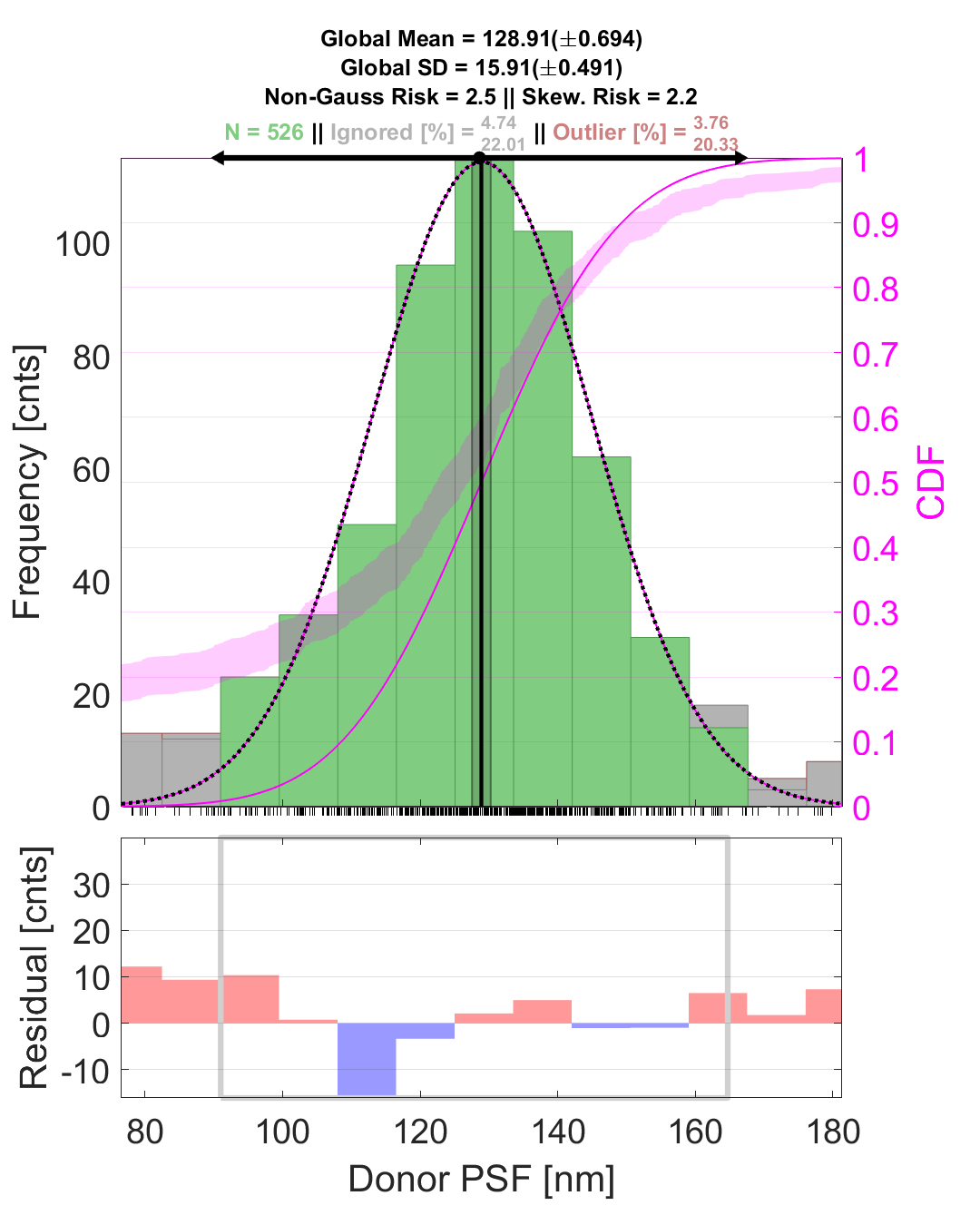

Supplement: Supplementary file 25 — Supplementary Software [file 41467_2024_49876_MOESM25_ESM.zip › FRET_efficiency_analysis/expected output/demo_cell_2/Donor_PSF.png]

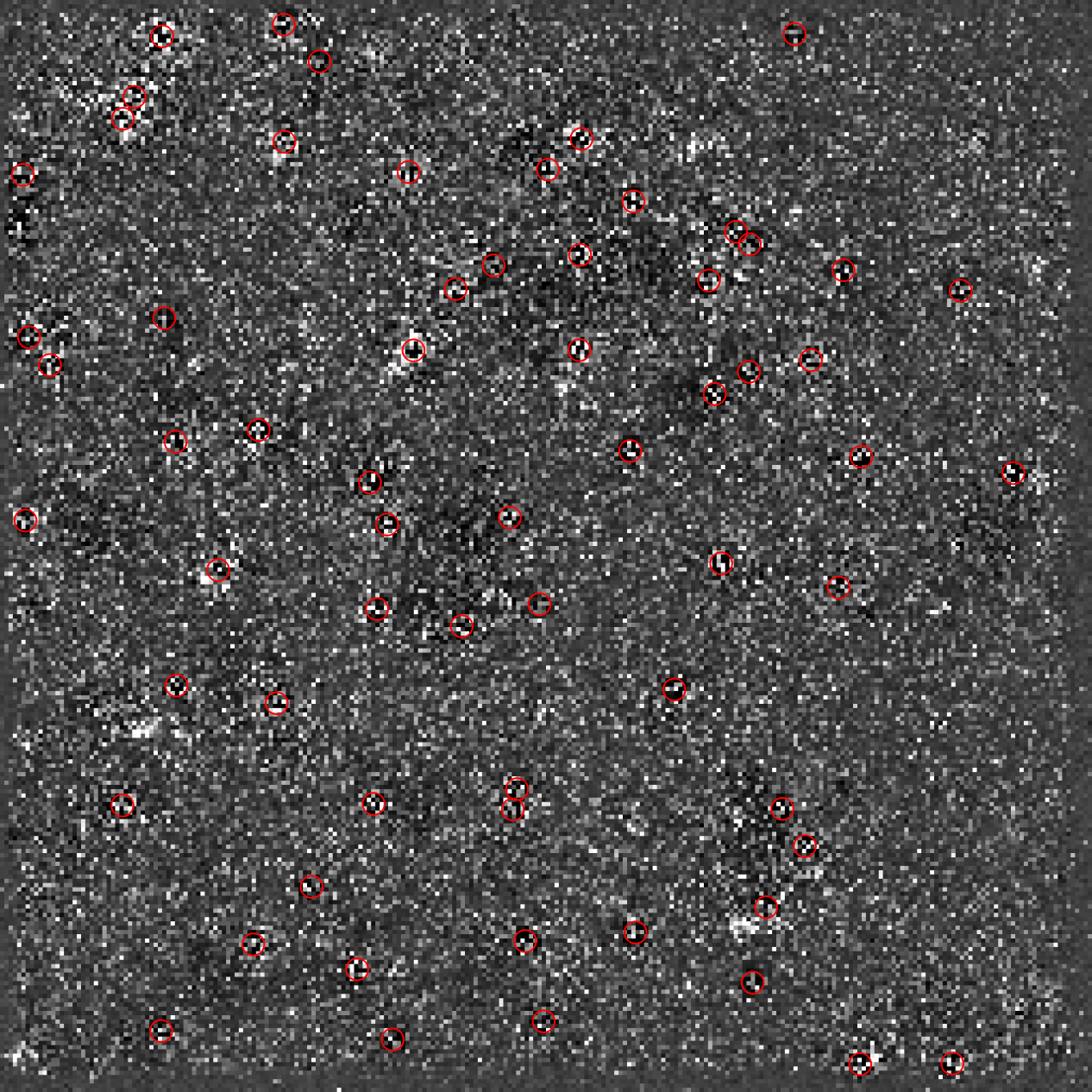

Supplement: Supplementary file 25 — Supplementary Software [file 41467_2024_49876_MOESM25_ESM.zip › FRET_efficiency_analysis/expected output/demo_cell_2/Donor_Res.png]

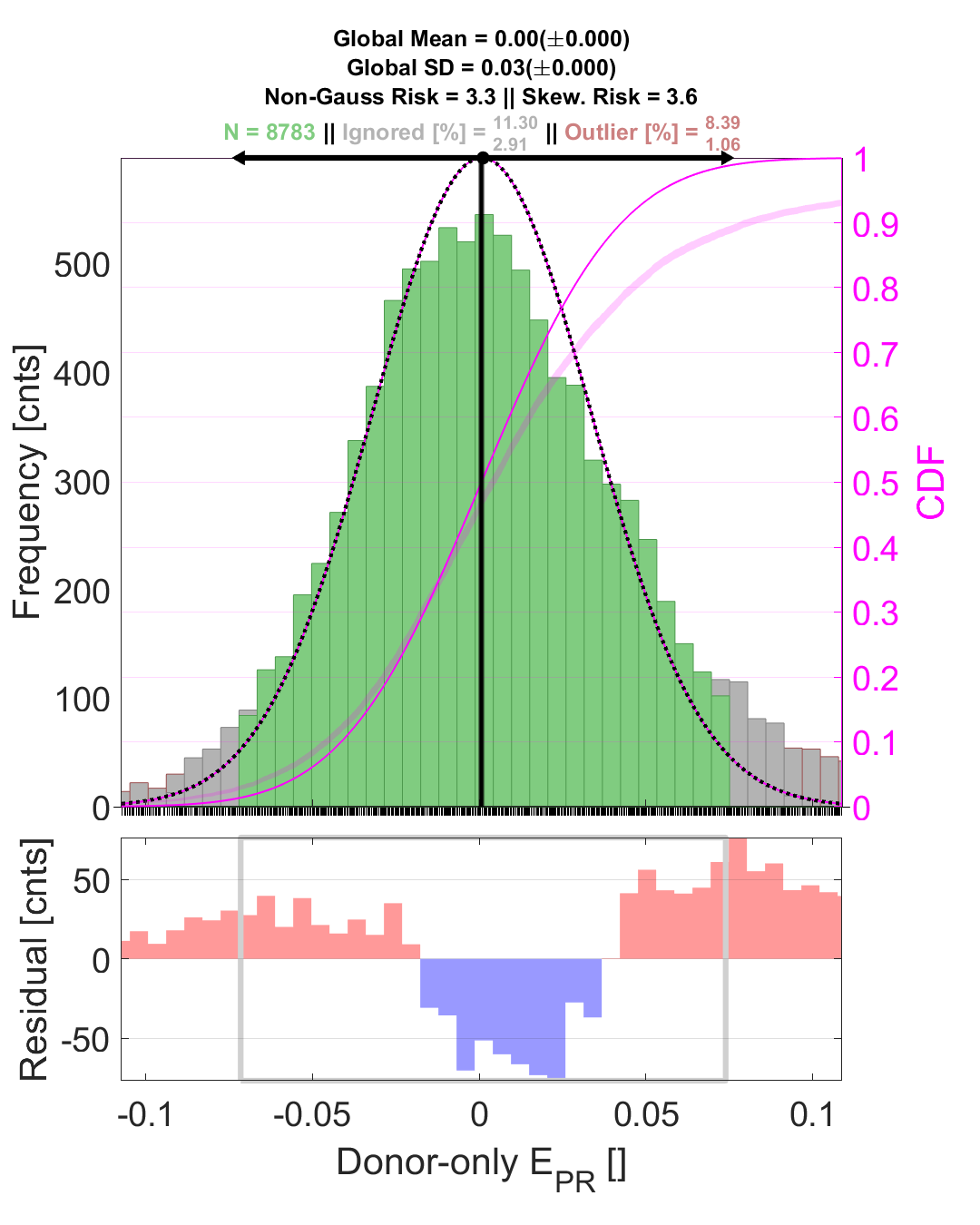

Supplement: Supplementary file 25 — Supplementary Software [file 41467_2024_49876_MOESM25_ESM.zip › FRET_efficiency_analysis/expected output/demo_cell_2/Donor-only EPR.png]

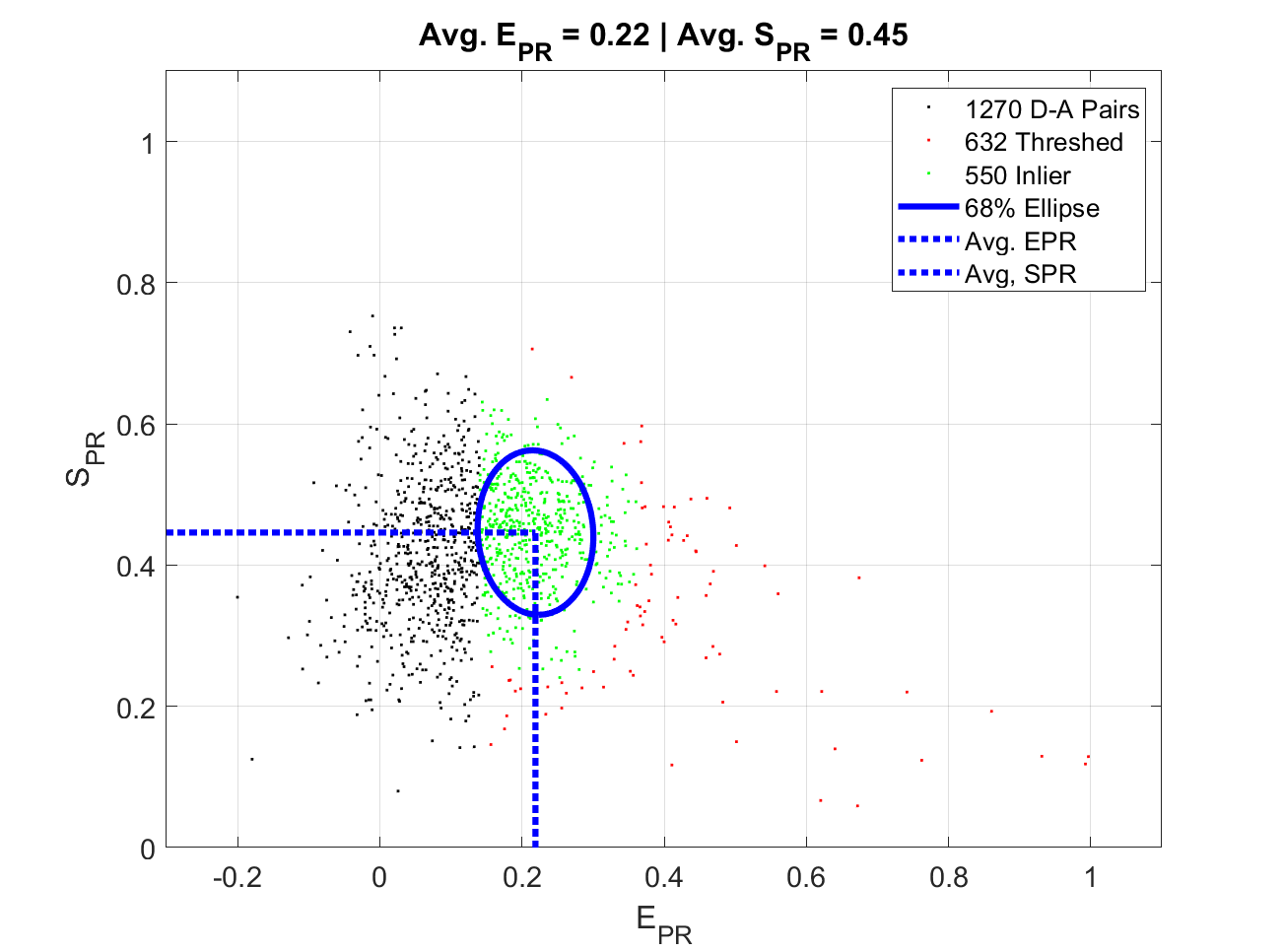

Supplement: Supplementary file 25 — Supplementary Software [file 41467_2024_49876_MOESM25_ESM.zip › FRET_efficiency_analysis/expected output/demo_cell_2/EPR_vs_SPR.png]

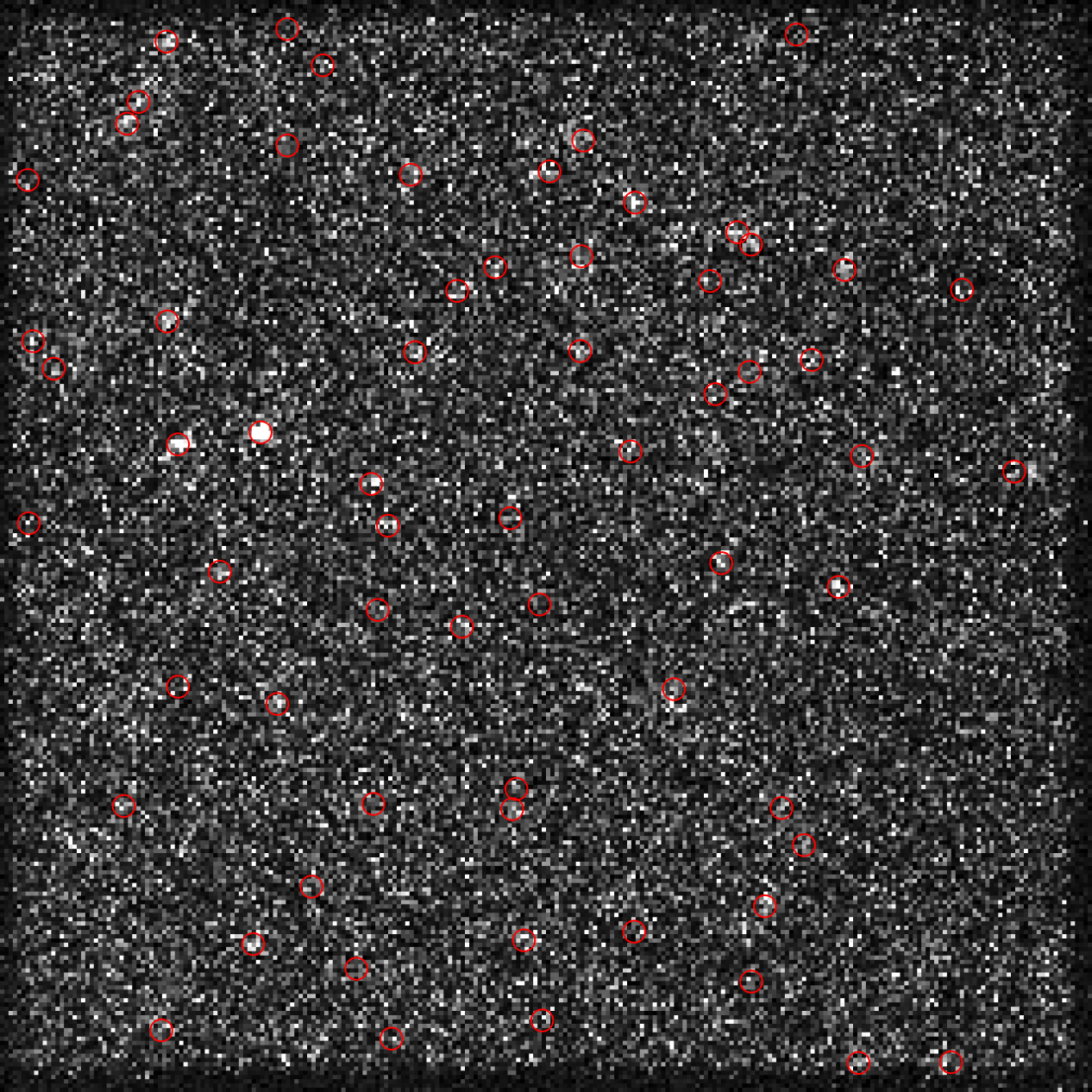

Supplement: Supplementary file 25 — Supplementary Software [file 41467_2024_49876_MOESM25_ESM.zip › FRET_efficiency_analysis/expected output/demo_cell_2/FRET_Localization.png]

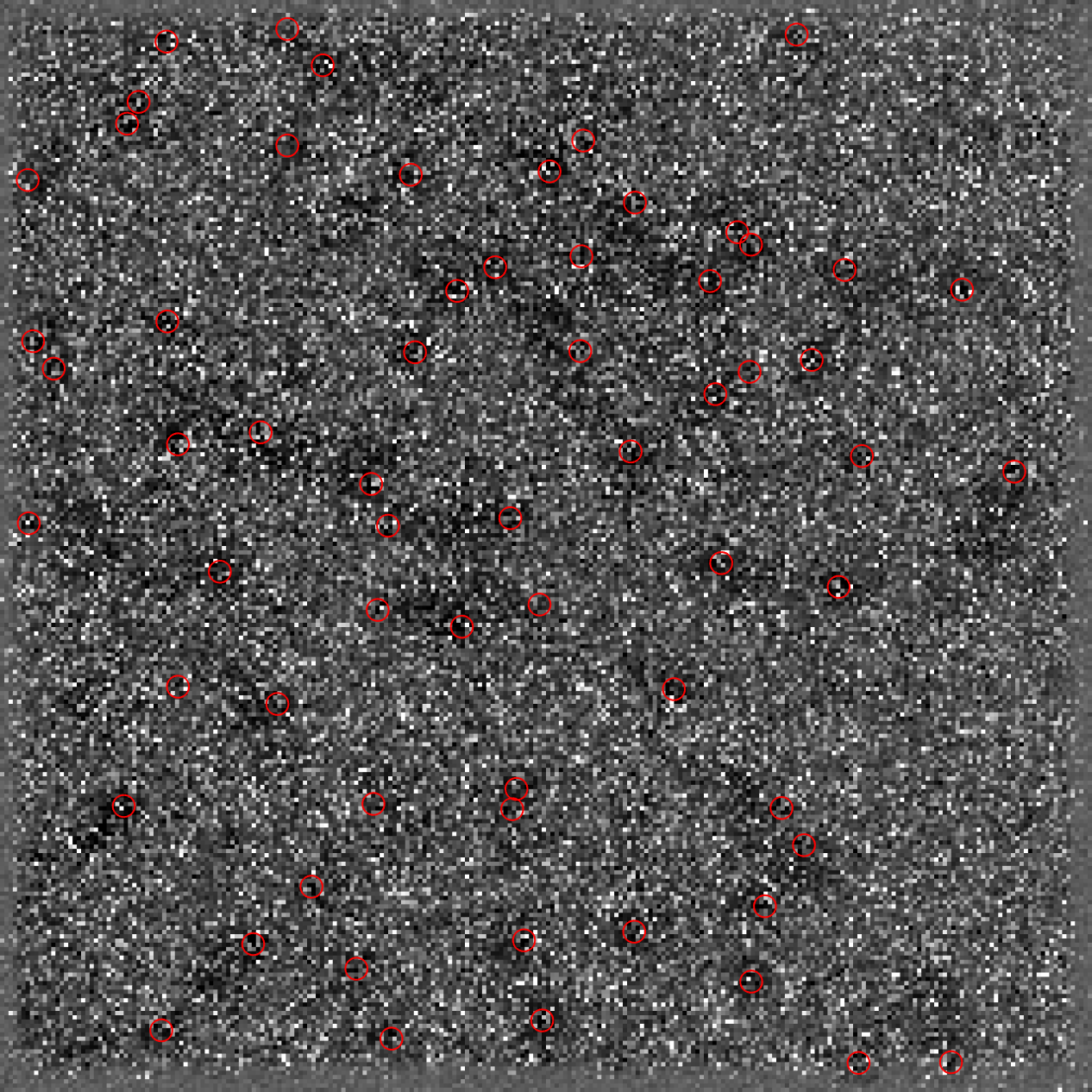

Supplement: Supplementary file 25 — Supplementary Software [file 41467_2024_49876_MOESM25_ESM.zip › FRET_efficiency_analysis/expected output/demo_cell_2/FRET_Res.png]

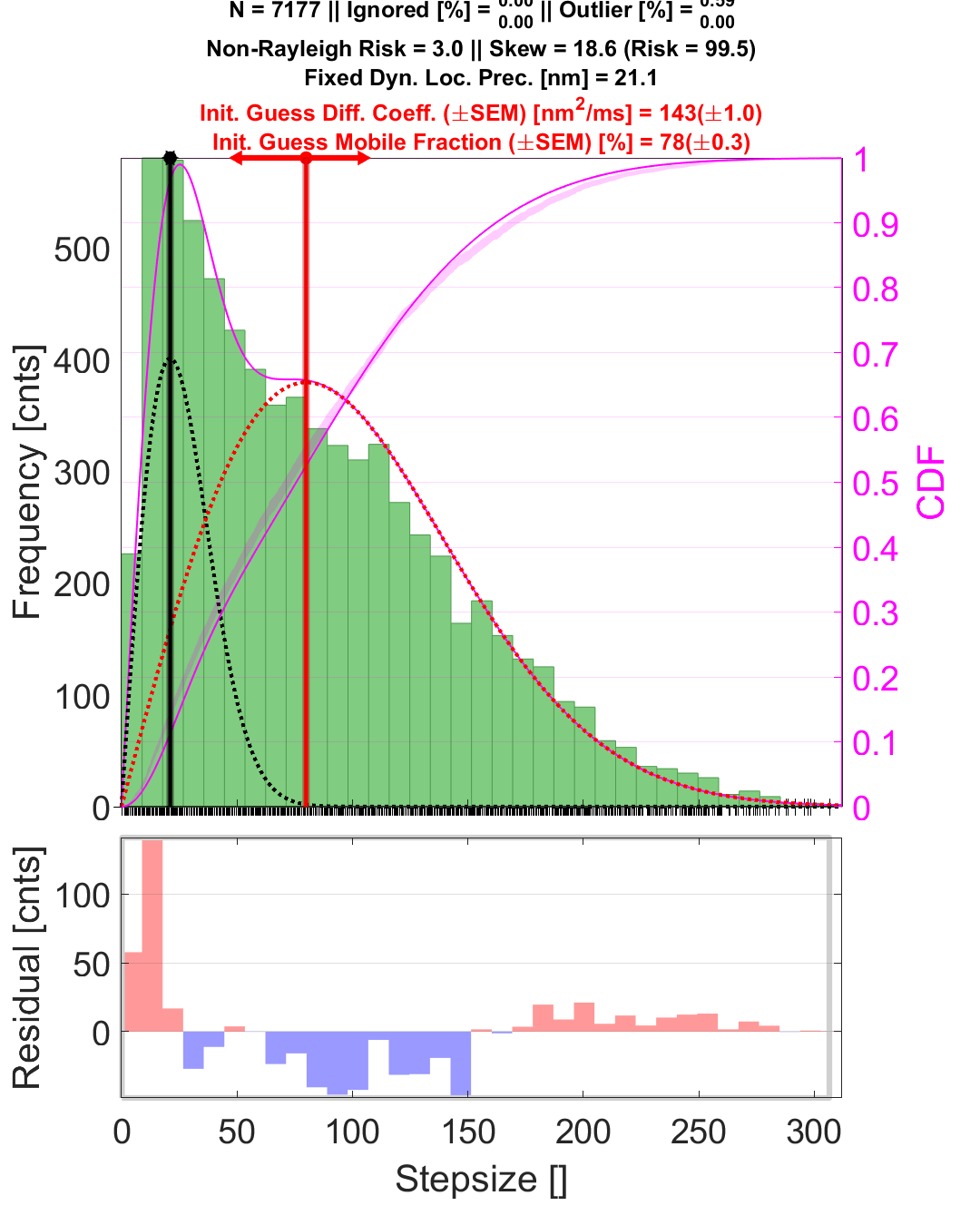

Supplement: Supplementary file 25 — Supplementary Software [file 41467_2024_49876_MOESM25_ESM.zip › FRET_efficiency_analysis/expected output/demo_cell_2/Stepsize.png]
